# Thiacycloalkynes for Copper-Free Click Chemistry**

**DOI:** 10.1002/anie.201106325

**Published:** 2012-01-26

**Authors:** Gabriela de Almeida, Ellen M Sletten, Hitomi Nakamura, Krishnan K Palaniappan, Carolyn R Bertozzi

**Affiliations:** Department of Chemistry, University of CaliforniaBerkeley, CA 94720 (USA); Department of Molecular and Cell Biology and Howard Hughes Medical Institute, University of CaliforniaBerkeley, CA 94720 (USA)

**Keywords:** bioorthogonal chemistry, click chemistry, protein modifications, strained alkynes, sulfur heterocycles

Bioorthogonal chemistry enables the interrogation of biomolecules and physiological processes that are inaccessible by using conventional research tools.^1^ A common experimental protocol starts by labeling a target biomolecule in cells or live organisms with a bioorthogonal functional group. Then, a probe molecule bearing complementary functionality is added to the system and the ensuing bioorthogonal chemical reaction delivers the probe specifically to the targets of interest. For many applications, rapid reaction kinetics are essential. This is particularly true for labeling experiments in live animals, in which reagent concentrations are limited (i.e., nm to low μm), or in which the process that is probed occurs on a fast time scale. Consequently, methodologists interested in the development of bioorthogonal reactions are increasingly focused on kinetic optimization.^2^

The strain-promoted cycloaddition reaction of azides and cyclooctynes, also called Cu-free “click” chemistry, is a bioorthogonal reaction that is well-tolerated by cells and the model organisms zebrafish, *Caenorhabditis elegans*, and mice (Scheme 1 a).^3^ The development of this reaction was inspired by the classic work of Wittig and Krebs in the 1960s.^4^ They observed that, in contrast to unactivated linear alkynes, which undergo 1,3-dipolar cycloaddition with azides only at elevated temperatures, cyclooctyne readily reacts with the same substrates at room temperature. The heightened reactivity of cyclooctyne was attributed to approximately 19 kcal mol^−1^ of ring strain resulting from deformation of the bond angles of the alkyne from 180° to 158°.^5^

**Scheme 1:**
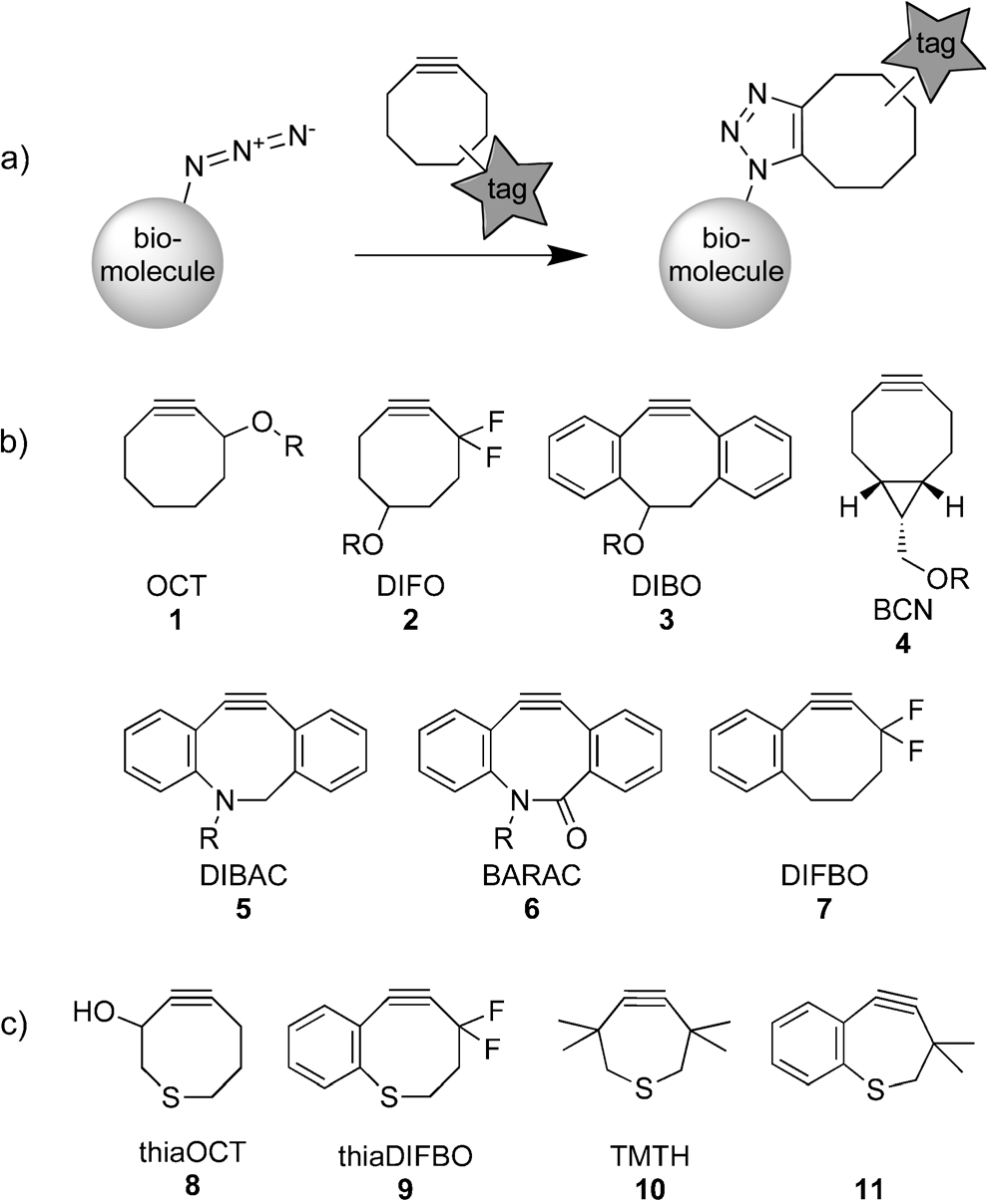
Copper-free click-chemistry probes. a) Azide-labeled biomolecules can be selectively labeled with cyclooctyne reagents; b) an array of cyclooctynes for copper-free click chemistry; c) thiacycloalkynes reported herein.

Capitalizing on this classic work, we and other research groups have reported numerous cyclooctyne analogues, which in comparison to linear alkynes display accelerated reaction rates with benzyl azide because of two major rate-enhancing modifications. Starting from the prototype OCT (**1**, Scheme 1 b),^6^ electronic activation was achieved through the addition of a *gem*-difluoro group as exemplified by DIFO (**2**). This compound reacts with benzyl azide with a second-order rate constant of *k*=7.6×10^−2^ m^−1^ s^−1^, which is 33 times higher than that exhibited by the parent compound OCT (**1**).^7^ The reactivity of cyclooctyne can also be enhanced by augmenting ring strain through the addition of fused phenyl or cyclopropyl rings as demonstrated by DIBO (**3**, *k*=5.7×10^−2^ m^−1^ s^−1^)^8^ and BCN (**4**, *k*=0.14 m^−1^ s^−1^), respectively.2i The further addition of sp^2^-like centers as in DIBAC (**5**, *k*=0.31 m^−1^ s^−1^)2d and BARAC (**6**, *k*=0.96 m^−1^ s^−1^)2c has led to reagents that are three orders of magnitude more reactive than OCT (**1**).

These experimental results, as well as a number of computational studies performed in parallel,^9^ have paved the way for the design of highly reactive cyclooctynes through a combination of activating factors. Unfortunately, unwanted side reactivity can result from such modifications. As an example, we sought to combine electronic activation with ring-strain enhancement by designing DIFBO (**7**).^10^ Although the compound reacted with azides quite rapidly (*k*=0.22 m^−1^ s^−1^), it also underwent spontaneous trimerization in solution. Notably, compounds such as BARAC react even more rapidly with azides, yet are otherwise quite stable. Goddard and coworkers proposed that the *ortho*-flagpole hydrogen atoms of biarylcyclooctynes such as BARAC can engage in unfavorable steric interactions with reaction partners to disfavor certain reaction pathways.9a Thus, the unusual combination of reactivity with azides and stability toward oligomerization may reflect a finely-tuned balance between the activating and stabilizing effects of the fused aryl rings of BARAC.

From this perspective, another approach to designing fast but stable cycloaddition substrates is to start with compounds such as DIFBO (**7**) and selectively eliminate their unwanted reactivities through the introduction of stabilizing modifications. One type of stabilizing perturbation that, unlike fused aryl rings, would not introduce steric bulk near the reaction center is an expansion of ring size. Indeed, the stabilities of cycloalkynes are stongly influenced by ring size, with cycloheptynes being unisolable at room temperature because of rapid oligomerization, and cyclononynes being several orders of magnitude less reactive with azides than cyclooctynes.^11^ We thought that a more subtle expansion of the cyclooctyne ring with a concomitant reduction in ring strain might be achieved by replacing a carbon atom with a larger sulfur atom. The length of a canonical C(sp^3^)–C(sp^3^) bond is 1.54 Å, whereas the length of a C(sp^3^)–S(sp^3^) bond is closer to 1.81 Å; this difference is significant, but more conservative than ring-size augmentation.

Herein, we explore the reactivities and stabilities of thiacycloalkynes. We found that incorporation of a sulfur atom into the cyclooctyne ring improves overall reagent stability but also reduces azide cycloaddition kinetics. We then combined this stabilizing modification with ring contraction, an activating feature previously unexplored in the context of Cu-free click chemistry, and identified a known thiacycloheptyne that is stable under ambient conditions but reacts with model azides faster than any reported cyclooctyne. These results suggest that thiacycloheptynes constitute a promising class of reagents for Cu-free click chemistry.

To assess the effects of an endocyclic sulfur atom on cyclooctyne reactivity, we first synthesized thiaOCT (**8**), the sulfur-containing analogue of OCT (**1**), from 1,6-dienyl-4-oxysilane **12** in eight steps (Scheme 2). Diene **12** was monoepoxidized by treatment with dimethyldioxirane^12^ and the resulting monoalkene **13** underwent a thiol-ene reaction with thioacetic acid to give **14**. Treatment of **14** with sodium hydride resulted in deprotection of the thiol and subsequent intramolecular cyclization to afford compound **15**. Protection of the free hydroxy group in **15** as a pivaloyl ester followed by removal of the TIPS group and oxidation with Dess–Martin periodinane gave ketone **17**.^13^ Compound **17** was transformed to the desired thiacyclooctyne **8** by *syn* elimination of vinyl triflate **18**.

**Scheme 2:**
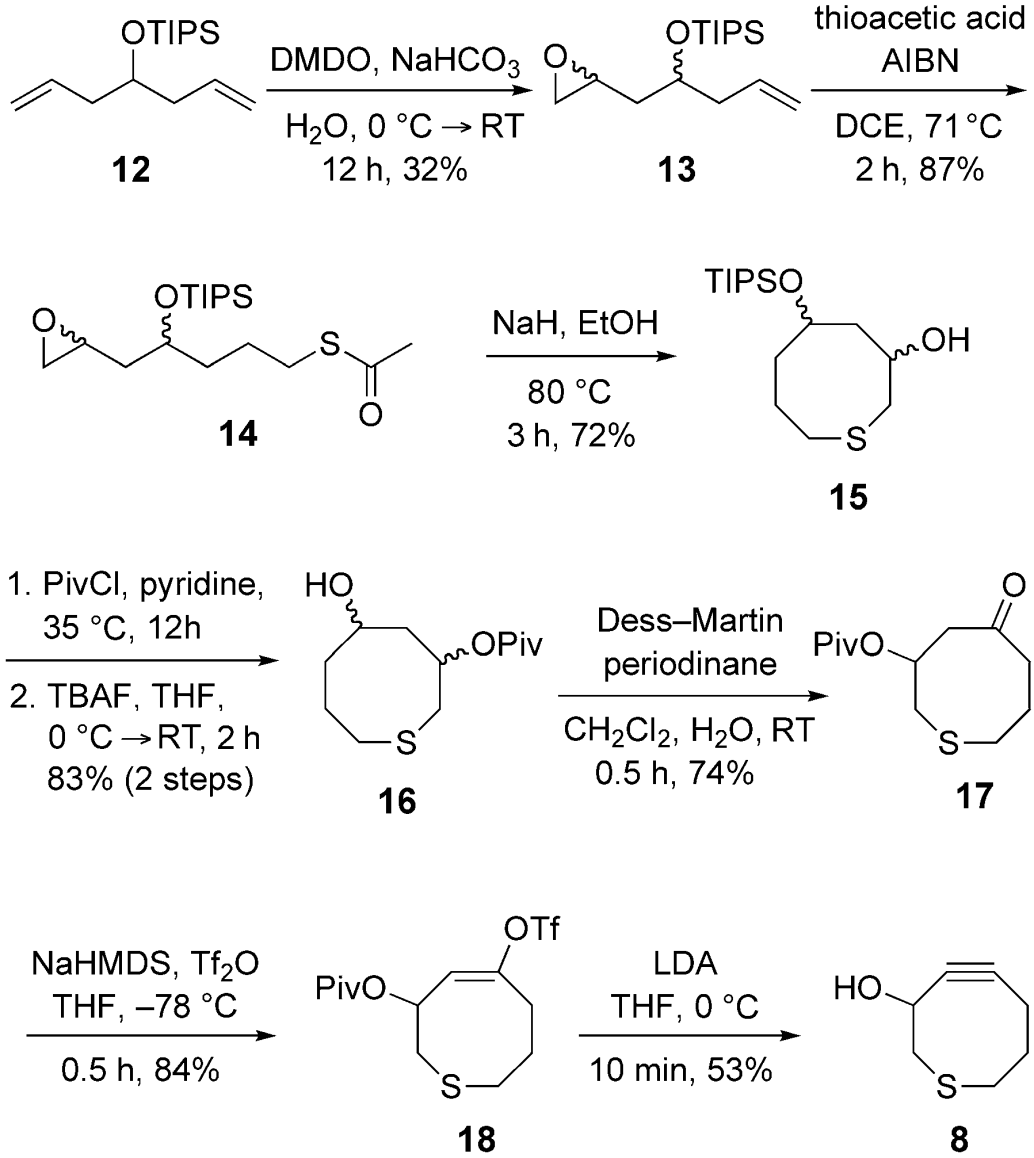
Synthesis of thiaOCT. AIBN=2,2′-azobisisobutyronitrile, DMDO=2,2-dimethyldioxirane, HMDS=1,1,1,3,3,3-hexamethyldisilazide, LDA=lithium diisopropylamide, Piv=pivaloyl, Tf=trifluoromethylsulfonyl, THF=tetrahydrofuran, TIPS=triisopropylsilyl.

We measured the second-order rate constant for the cycloaddition reaction of thiaOCT and benzyl azide in CD_3_CN (3.2×10^−4^ m^−1^ s^−1^, see Figure S1 in the Supporting Information), and it was almost one order of magnitude lower than that of the parent compound OCT. The small ring expansion achieved by the substitution with a sulfur atom caused a significant reduction in dipolar-cycloaddition reactivity.^14^

We next explored whether an endocyclic sulfur atom could improve the stability of DIFBO (**7**) by tempering the activating effects of the fused aryl ring and propargylic *gem*-difluoro group. ThiaDIFBO (**9**) was prepared similarly to DIFBO (**7**) and relied on a key ring expansion of **19** by using TMSCHN_2_ (see Scheme 3, and Scheme S1 in the Supporting Information). We were pleased to find that, unlike DIFBO, thiaDIFBO did not oligomerize during concentration and storage, thus confirming the dramatic stabilizing effect of the endocyclic sulfur atom. The second-order rate constant for cycloaddition with benzyl azide in CD_3_CN was found to be 1.4×10^−2^ m^−1^ s^−1^, almost twenty times lower than that of DIFBO (see Figure S2 in the Supporting Information).^10^ Notably, this rate constant is comparable to that of previously reported monobenzocyclooctyne MOBO,^10^ which is identical to DIFBO but lacks the fluorine atoms. The similar reactivities of these two compounds suggest that the substantial activating effect of the *gem*-difluoro group is directly balanced by the stabilizing influence of the sulfur atom.

**Scheme 3:**
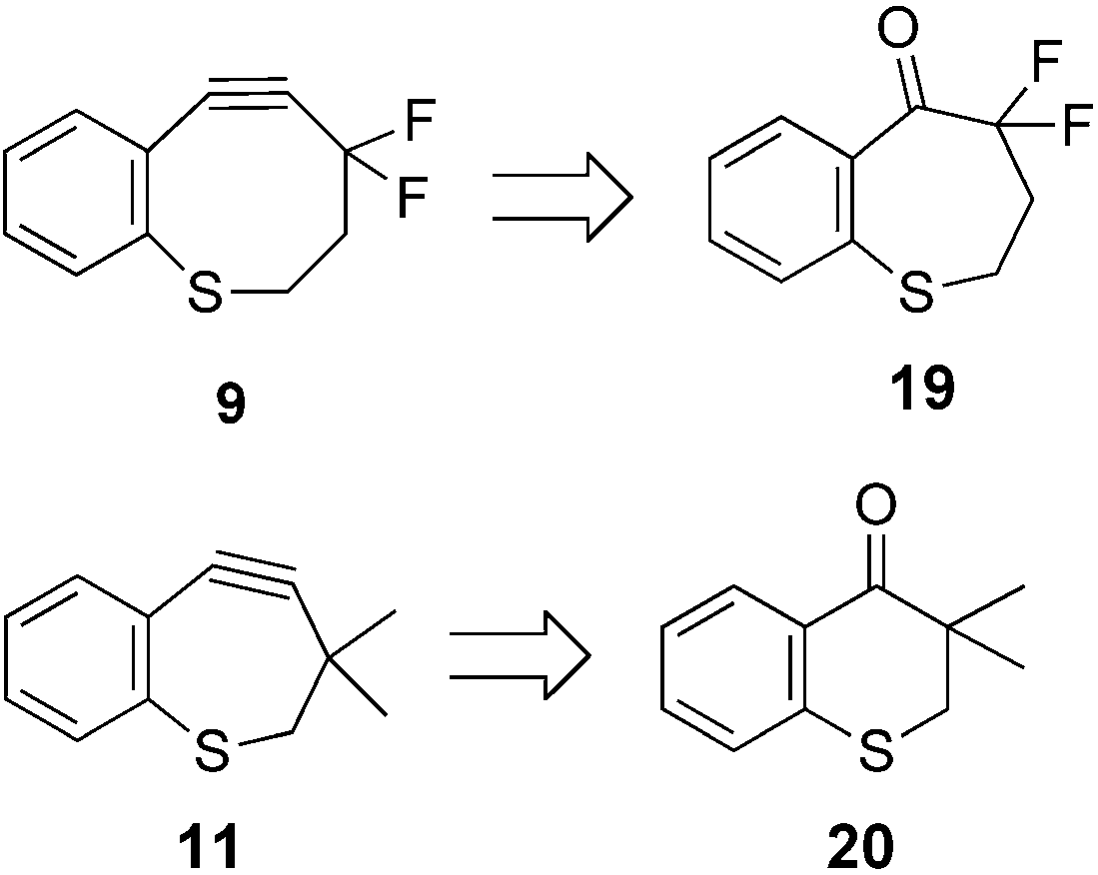
Retrosynthetic analysis of thiaalkynes **9** and **11**.

The stability of thiaDIFBO provided an opportunity to enhance reactivity through a more dramatic structural modification: the contraction to a seven-membered thiacycloheptyne ring, a motif that has not yet been explored in the context of bioorthogonality. Krebs and Kimling reported in the 1970s that 3,3,6,6-tetramethylthiacycloheptyne (TMTH, **10**) is indeed a stable cycloalkyne, and that it is known to react with phenyl azide and other 1,3 dipoles in a manner that correlates with its increased ring strain, although specific rate constants for the reaction with 1,3 dipoles were not reported.^15^ Furthermore, although acetic acid adds to **10** over 10 000 times faster than to cyclooctyne, the rate constant for this addition is 1.41×10^−5^ m^−1^ s^−1^,^5^ which makes the reaction significantly slower than the azide–alkyne cycloaddition reaction with the more temperate reagent OCT. Given this precedent, we presumed that the smaller ring size of TMTH would promote the cycloaddition with azides at faster rates than exhibited by cyclooctyne, and that the methyl groups at the propargylic positions would shield the alkyne from unwanted side reactions.

We synthesized TMTH in a manner similar to that reported previously (see Scheme S2 in the Supporting Information).^15^ Its reaction with benzyl azide in CD_3_CN proceeded cleanly with a second-order rate constant of (4.0±0.4) m^−1^ s^−1^, the fastest reported cycloalkyne–azide reaction to date (see Figures S3 and S4 in the Supporting Information). During the preparation of this manuscript, Banert and Plefka reported that TMTH was more reactive than a DIBO-like cyclooctyne in a 1,3-dipolar cycloaddition with nitrous oxide,^16^ a result that is consistent with our findings.

Motivated by our results, we sought to further enhance the reactivity of thiacycloheptynes through the addition of a fused aryl ring. We attempted to synthesize the seven-membered thiaDIFBO analogue **11** in which the activating propargylic *gem-*difluoro group was replaced with the stabilizing propargylic *gem*-dimethyl group, but we were unable to isolate **11** or any identifiable oligomerization or degradation products thereof (see Scheme S3 in the Supporting Information). Compound **11** could be formed transiently, as evidenced by the formation of triazole products when the final alkyne-generating reaction was performed in the presence of benzyl azide, but **11** was clearly too unstable for practical purposes.

We thus returned to TMTH, whose unique combination of azide reactivity and stability prompted us to explore its potential as a bioorthogonal reagent. Importantly, TMTH proved to be stable in water and phosphate-buffered saline (PBS) and far less reactive with biologically relevant thiols than with azides (see Figures S5 and S6 in the Supporting Information). TMTH is known to undergo oxidation in air to form the corresponding diketone,^17^ but we saw no evidence for this particular side reaction in our experiments under aqueous conditions. Some degradation of TMTH in water was observed, giving a complex product mixture, but the process was slow on the time scale of most biological labeling experiments (see Figure S5 in the Supporting Information).

We next tested the reactivity of TMTH with azide-functionalized biomolecules. Indirect evidence of the ability of TMTH to selectively label azide-modified glycoproteins was obtained in an azide-blocking experiment with TMTH and phosphine–FLAG, which reacts with azides through a Staudinger ligation.^18^ Jurkat cells were treated with peracetylated *N*-azidoacetylgalactosamine (Ac_4_GalNAz), which metabolically labels cell-surface glycoproteins with GalNAz residues and intracellular proteins with GlcNAz residues.^19^ Cell lysates were generated and incubated with TMTH at concentrations ranging from 1 nm to 1 mm for 1.5 hours at room temperature. The progress of the reaction was determined by the detection of unreacted azides with 500 μm phosphine–FLAG (Figure 1 a). Western-blot analysis indicated that pretreatment with TMTH diminished phosphine–FLAG labeling of cell lysates in a dose-dependent manner (Figure 1 b), thus implying that TMTH reacts with azide-bearing glycoproteins. Similar results were obtained through a direct competition experiment between TMTH and phosphine–FLAG (see Figures S8 and S9 in the Supporting Information).

**Figure 1:**
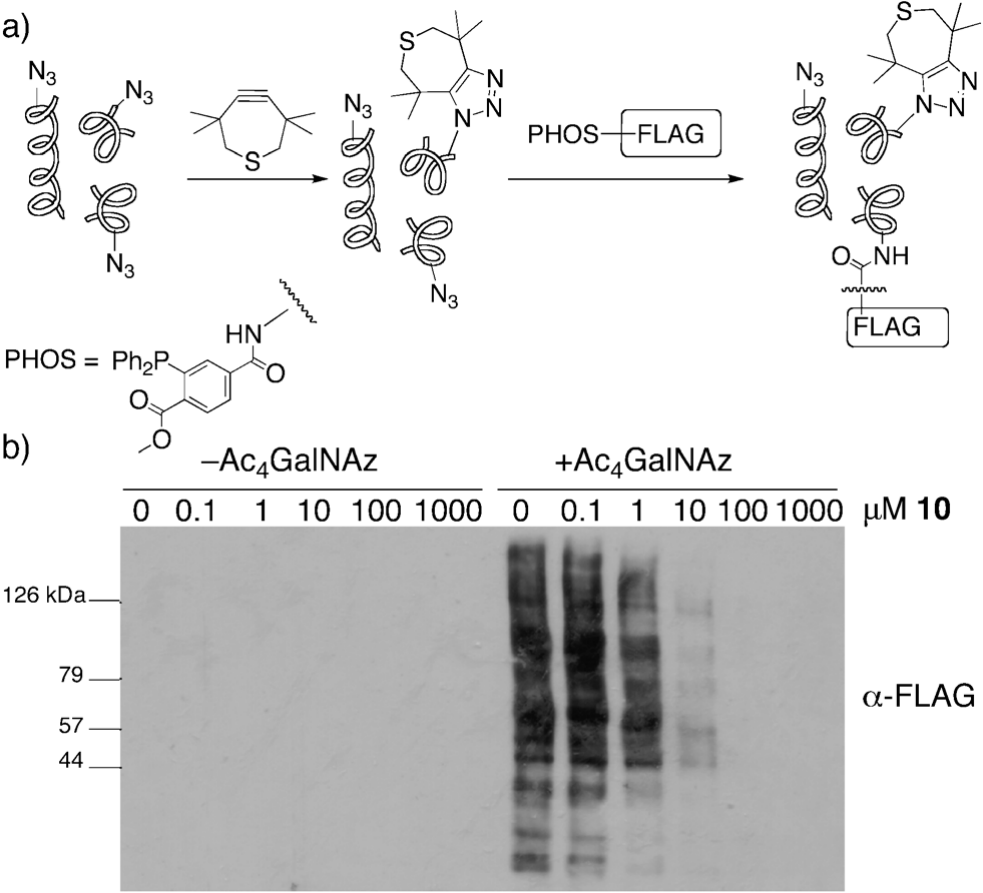
Pretreatment of Ac_4_GalNAz-labeled cell lysates with TMTH (**10**) results in inhibition of phosphine–FLAG (PHOS–FLAG) labeling. Jurkat cells were incubated with Ac_4_GalNAz (50 μm) or vehicle (DMSO) for 3 days, and then lysed. a) The lysates were treated with TMTH (concentration 1 nm–1 mm) for 1.5 h followed by 500 μm phosphine–FLAG overnight; b) the lysates were analyzed by Western blot by using a primary anti-FLAG antibody and a secondary antibody conjugated to horseradish peroxidase. India Ink staining confirmed equal protein loading (see Figure S7 in the Supporting Information). DMSO=dimethyl sulfoxide.

To obtain direct evidence of the reactivity of TMTH with azides, we sought to demonstrate selective labeling of an azide-functionalized protein. By expressing the small protein barstar from *B. cenocepacia* (11.7 kDa) in a methionine auxotrophic *E. coli* cell line, we generated a variant in which both methionine (MET) residues were replaced with azidohomoalanine (AHA) residues (barstar-AHA). The barstar-AHA protein and its methionine counterpart (barstar-MET) were treated with TMTH or vehicle in PBS at room temperature for 120 hours. The intact proteins were analyzed by LC-MS (see the Supporting Information). When reacted with TMTH, only the barstar-AHA sample showed the shift in mass (168 Da) expected for TMTH-modified barstar (Figure 2), thus confirming that the cycloaddition reaction occurred in a selective manner. The reaction yield was unexpectedly modest, considering the intrinsic kinetics of the TMTH–azide cycloaddition. This may indicate that steric hindrance of the azide (versus benzyl azide) slowed the reaction so that some TMTH, the half-life of which is approximately 48 hours in aqueous media (see Figure S5 in the Supporting Information), degraded before the reaction was complete.

**Figure 2:**
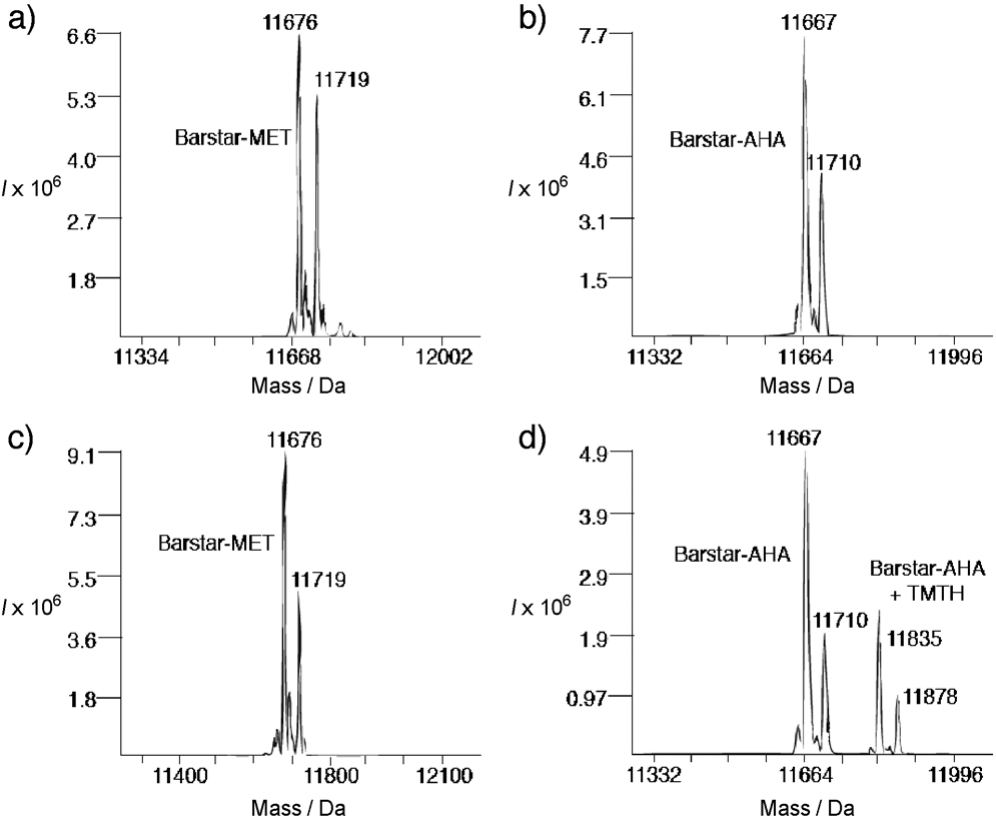
TMTH selectively labels barstar containing azidohomoalanine (barstar-AHA). Barstar-AHA (b, d) or wildtype barstar (barstar-MET; a, c) in PBS was treated with TMTH (c, d) or vehicle (DMSO; a, b) for 120 h and then analyzed by mass spectrometry. Only barstar-AHA showed the expected mass shift of 168 Da. The mass shifts of 43 Da correspond to the N-terminal acylated barstar species.

In conclusion, thiacycloheptynes are a promising new class of reagents for bioorthogonal Cu-free click chemistry. More broadly, the use of endocyclic heteroatoms to fine-tune ring strain offers a new mechanism to balance reactivity and stability when designing new bioorthogonal reagents. One of the greatest challenges in the design of these reagents is that, unlike for conventional chemistry, in which unwanted side reactivities can be mitigated by changing the reaction conditions, bioorthogonal reagents must be stable while reacting rapidly and selectively in a highly functionalized environment in which many reaction pathways are possible. An emerging theme in the design of bioorthogonal reagents for Cu-free click chemistry involves optimization for the desired reaction pathway through activating and deactivating modifications. Herein, we have identified the decreased ring strain that resulted from substitution with an endocyclic sulfur as a new tool for such precise reagent engineering. In the future, other large heteroatoms in various oxidation states could provide improved precision when tuning reactivity and stability.^20^

## Supplementary material

Detailed facts of importance to specialist readers are published as ”Supporting Information”. Such documents are peer-reviewed, but not copy-edited or typeset. They are made available as submitted by the authors.
