# Supplementary material for: Thiacycloalkynes for Copper-Free Click Chemistry
Source: Angew Chem Int Ed Engl. 2012 Jan 26;51(10):2443–7. doi: 10.1002/anie.201106325 (PMC3384729; doi:10.1002/anie.201106325)

Angewandte Chemie
Eine Zeitschrift der Gesellschaft Deutscher Chemiker

Supporting Information
© Wiley-VCH 2012
69451 Weinheim, Germany

Thiacycloalkynes for Copper-Free Click Chemistry**

*Gabriela de Almeida, Ellen M. Sletten, Hitomi Nakamura, Krishnan K. Palaniappan, and Carolyn R. Bertozzi**

anie_201106325_sm_miscellaneous_information.pdf

Table of Contents

General synthetic procedures……………………………………………………………S3

Synthesis of thiaOCT……………………………………………………………………S3

NMR kinetics of thiaOCT (Figure S1)…………………………………………………S7

Synthesis of thiaDIFBO (Scheme S1)……………………………………………………S8

NMR kinetics of thiaDIFBO (Figure S2)………………………………………………S11

Synthesis of TMTH (Scheme S2)………………………………………………………S11

NMR kinetics of TMTH (Figures S3-S4)………………………………………………S14

Synthesis of **11** (Scheme S3)……………………………………………………………S16

TMTH stability to D_2O and PBS (Figure S5)……………………………………………S19

TMTH reactivity with azides and glutathione in PBS (Figure S6)……………………S21

Western blot competition experiment (Figures S7-S9)…………………………………S22

Barstar expression and LC-MS analysis of protein samples……………………………S24

Synthesis of thiaOCT-biotin (Scheme S4)………………………………………………S25

Procedure for cell labeling………………………………………………………………S26

Cell labeling and toxicity of thaiOCT-Biotin (Figures S10-S14)………………S27

^{1}H, ^{13}C, ^{19}F NMR Spectra of Compounds……………………………………………S32

General Procedures

All chemical reagents were purchased from Sigma-Aldrich, Acros, or TCI chemicals and used without purification unless noted otherwise. Solvents were purified as described by Pangborn *et al.*[1] In all cases, magnesium sulfate or sodium sulfate were used as drying agents and solvent was removed by reduced pressure with a Buchi Rotovapor R-114 equipped with a Welch self-cleaning dry vacuum. Non-volatile products were further dried by reduced pressure with an Edwards RV5 high vacuum. Thin layer chromatography was performed with EMD 60 Å silica gel plates. Unless otherwise specified, R_f values are reported in the solvent system the reaction was monitored in. Flash chromatography was performed using Silicycle® 60 Å 230-400 mesh.
All ^{1}H, ^{13}C, and ^{19}F NMR spectra are reported in ppm and referenced to solvent peaks. Spectra were obtained on Bruker AV-300, AVB-400, AVQ-400, DRX-500, or AV-500, AV-600 instruments. High resolution electron ionization (EI) and electrospray ionization (ESI) mass spectra were obtained from the UC Berkeley Mass Spectrometry Facility.

Experimental Procedures

(Hepta-1,6-dien-4-yloxy)triisopropylsilane (12).

NaH (60 % w/w in mineral oil, 0.46 g, 12 mmol, 3.3 equiv) was dissolved in THF (5.0 mL, anhydrous) and cooled to 0 °C. To this mixture, hepta-1,6-dien-4-ol (0.50 mL, 3.5 mmol, 1.0 equiv) was added dropwise and the mixture was stirred at 0 °C. After 30 min, triisopropylsilyl chloride (1.5 mL, 7.0 mmol, 2.0 equiv) was added to the reaction mixture. The reaction was warmed to rt and stirred overnight. The following day, the reaction was quenched with an aqueous solution of saturated ammonium chloride (8 mL). The product was extracted into dichloromethane (3 x 10 mL) and the combined organics were dried with $MgSO_4$, decanted and evaporated to dryness. The crude product was purified by silica gel chromatography (hexane) to give desired product in quantitative yield (930 mg, 3.5 mmol). R_f = 0.95 in 3:1 hexanes/EtOAc. ^{1}H NMR (500 MHz, $CDCl_3$): δ 5.84 (ddd, *J* = 16.3, 13.9, 7.2 Hz, 2H), 5.06-5.03 (m, 4H), 3.91 (p, *J* = 5.8 Hz, 1H), 2.34-2.28 (m, 4H), 1.07-1.04 (m, 21H). This compound was previously reported by Livinghouse and coworkers.[2]

Triisopropyl((1-(oxiran-2-yl)pent-4-en-2-yl)oxy)silane (13).

(Hepta-1,6-dien-4-yloxy)triisopropylsilane **12** (2.4 g, 9.0 mmol, 1.0 equiv) was dissolved in dichloromethane (74 mL). To this solution, an aqueous solution of saturated sodium bicarbonate (124 mL) and acetone (7.4 mL, 0.10 mol, 11 equiv) was added and the reaction mixture was cooled to 0 °C. To the vigorously stirring reaction mixture, oxone (22 g, 36 mmol, 4.0 equiv) dissolved in water (87 mL) was added dropwise using an addition funnel. The reaction mixture was slowly warmed to rt and stirred overnight. The organic layer was separated from the aqueous layer. The aqueous layer was extracted with dichloromethane (2 x 100 mL). The combined organics were dried with $MgSO_4$, decanted and evaporated to dryness to give crude mixture of unreacted starting material, desired product, and diepoxidized byproduct. The crude product was purified by silica gel

chromatography (100:1 to 10:1 hexane/EtOAc). This procedure yielded desired product as a 1:0.8 mixture of two diastereomers (1.1 g, 2.9 mmol, 32 %) plus some recovered starting material and ((1,3-di(oxiran-2-yl)propan-2-yl)oxy)triisopropylsilane. R_f = 0.8 in 3:1 hexanes/EtOAc. ^{1}H NMR (500 MHz, $CDCl_3$): major diastereomer δ 5.86-5.73 (m, 1H), 5.08-5.02 (m, 2H), 4.11-4.08 (m, 1H), 3.07-3.03 (m, 1H), 2.77 (t, *J* = 4.9 Hz, 1H), 2.46-2.31 (m, 3H), 1.76-1.61 (m, 2H), 1.07-1.03 (m, 21H); minor diastereomer δ 5.86-5.73 (m, 1H), 5.08-5.02 (m, 2H), 4.11-4.08 (m, 1H), 3.07-3.03 (m, 1H), 2.73 (t, *J* = 4.9 Hz, 1H), 2.46-2.31 (m, 3H), 1.76-1.61 (m, 2H), 1.07-1.03 (m, 21H). ^{13}C NMR (125 MHz, $CDCl_3$): major diastereomer δ 134.8, 117.8, 70.5, 50.0, 47.3, 42.8, 40.1, 18.5, 13.0; minor diastereomer δ 135.0, 117.8, 70.6, 49.6, 48.1, 42.0, 39.7, 18.6, 12.9. HRMS (ESI): calcd for $C_{16}H_{32}O_2NaSi^+$ $[M + Na]^+$, 307.2064; found, 307.2061.

5-(oxiran-2-yl)-4-((triisopropylsilyl)oxy)pentyl ethanethioate (14).

Triisopropyl((1-(oxiran-2-yl)pent-4-en-2-yl)oxy)silane **13** (660 mg, 2.3 mmol, 1.0 equiv) was dissolved in dichloroethane (20 mL). To this solution, thioacetic acid (550 μL, 7.7 mmol, 3.3 equiv) and azobisisobutyronitrile (AIBN) (130 mg, 0.79 mmol, 0.34 equiv, recrystallized from methanol) were added. The reaction mixture was heated to reflux for 2 h at which point the mixture was cooled to rt. The organic layer was washed with an aqueous solution of saturated sodium bicarbonate (2 x 20 mL) and water (20 mL). The aqueous layer was extracted with dichloromethane (25 mL) and the combined organics were dried with $MgSO_4$, decanted and evaporated to dryness. The crude product was purified by silica gel chromatography (90:1 to 25:1 hexane/EtOAc) to yield the desired product as a 1:0.4 mixture of two diastereomers (720 mg, 2.0 mmol, 87 %). R_f = 0.6 in 4:1 hexanes/EtOAc. ^{1}H NMR (500 MHz, $CDCl_3$): major diastereomer δ 4.05-4.01 (m, 1H), 3.03-3.01 (m, 1H), 2.87-2.85 (m, 2H), 2.77 (t, *J* = 4.5, 1H), 2.48-2.43 (m, 2H), 2.32 (s, 3H), 1.65-1.58 (m, 6H), 1.07-1.03 (m, 21H); minor diastereomer δ 4.05-4.01 (m, 1H), 3.03-3.01 (m, 1H), 2.87-2.85 (m, 2H), 2.75 (t, *J* = 4.7, 1H), 2.48-2.43 (m, 2H), 2.32 (s, 3H), 1.83-1.65 (m, 6H), 1.07-1.03 (m, 21H). ^{13}C NMR (150 MHz, $CDCl_3$): major diastereomer δ 195.9, 70.1, 49.6, 47.4, 39.9, 36.6, 30.6, 29.3, 25.0, 18.1, 12.6; minor diastereomer δ 195.5, 70.0, 48.9, 46.8, 39.4, 35.7, 30.6, 29.3, 24.7, 18.1, 12.5. HRMS (ESI): calcd for $C_{18}H_{36}O_3NaSSi^+$ $[M + Na]^+$, 383.2047; found, 383.2048.

5-((triisopropylsilyl)oxy)thiocan-3-ol (15).

NaH (60 % w/w in mineral oil, 20 mg, 0.50 mmol, 8.9 equiv) was added to a round-bottom flask followed by slow addition of ethanol (10 mL). 5-(oxiran-2-yl)-4-((triisopropylsilyl)oxy)pentyl ethanethioate **14** (20 mg, 0.056 mmol, 1.0 equiv) dissolved in ethanol (5.0 mL) was added dropwise and the reaction mixture was heated to reflux. After 3 h, the reaction mixture was cooled to rt and the ethanol was removed by rotary evaporation. The viscous liquid was dissolved in dichloromethane (20 mL), and the organic layer was washed with an aqueous solution of saturated ammonium chloride (25 mL). The aqueous layer was extracted with dichloromethane (2 x 20 mL) and the combined organics were dried with $MgSO_4$, decanted and evaporated to dryness. The crude product was purified by silica gel chromatography (12:1 hexane/EtOAc). This procedure resulted in 13 mg of desired product as a 1:0.85 mixture of two diastereomers

(0.040 mmol, 72 %). R_f = 0.5 and 0.45 in 3:1 hexanes/EtOAc. ^{1}H NMR (500 MHz, $CDCl_3$): mixture of diastereomers δ 4.24-4.19 (m, 1H, 1H'), 4.17- 4.13 (m, 1H), 3.93 (bs, 1H'), 3.51 (bs, 1H'), 3.03 (dd, *J* = 15.1, 5.6 Hz, 1H'), 2.89 (qd, *J* = 15.2, 4.4 Hz, 2H), 2.73- 2.67 (m, 2H, 1H'), 2.64- 2.53 (m, 1H, 1H'), 2.36 (dt, *J* = 14.9, 2.6 Hz, 1H'), 2.25 (ddd, *J* = 14.4, 9.0, 2.2 Hz, 1H), 2.20-2.13 (m, 1H'), 2.08-1.98 (m, 1H, 2H'), 1.96-1.84 (m, 2H), 1.80-1.50 (m, 2H, 3H'), 1.03 (m, 21H, 21H'). ^{1}H NMR (500 MHz, $CDCl_3$): major diastereomer *δ* 4.21 (m, 1H), 4.15-4.16 (m, 1H), 2.89 (qd, *J* = 15.2, 4.4 Hz, 2H), 2.72-2.68 (m, 2H), 2.60-2.56 (m, 1H), 2.25 (dd, *J* = 12.0, 7.5 Hz, 1H), 2.03 (dd, *J* = 12.1, 3.2 Hz, 1H), 1.93-1.89 (m, 2H), 1.74-1.58 (m, 2H), 1.07-1.03 (m, 21H); ^{13}C NMR (150 MHz, $CDCl_3$): major diastereomer δ 68.5, 67.1, 40.7, 39.4, 34.8, 33.3, 24.6, 18.1, 12.3 HRMS (ESI): calcd for $C_{16}H_{34}O_2NaSSi^+$ [M + Na]$^+$, 341.1941; found, 341.1941.

5-((triisopropylsilyl)oxy)thiocan-3-yl pivalate.

5-((triisopropylsilyl)oxy)thiocan-3-ol **15** (231 mg, 0.726 mmol, 1.0 equiv) was dissolved in pyridine (11.5 mL, anhydrous). To this solution, pivaloyl chloride (350 *μ*L, 2.9 mmol, 3.9 equiv) was added. The reaction mixture was then warmed to 35 °C and stirred overnight. The reaction was cooled to rt and evaporated to dryness. The crude product was purified by silica gel chromatography (25:1 hexane/EtOAc) to afford the desired product in 80 % yield as a 1:1 mixture of diastereomers (243 mg, 0.581 mmol). R_f = 0.8 in 3:1 hexane/EtOAc. ^{1}H NMR (500 MHz, $CDCl_3$): δ 5.25-5.19 (m, 1H), 4.83-4.79 (m, 1H), 4.32-4.25 (m, 1H), 4.16-4.11 (m, 1H), 2.95 (dd, *J* = 15.1, 4.8 Hz, 1H), 2.79-2.63 (m, 6H), 2.61-2.55 (m, 1H), 2.30 (ddd, *J* = 14.6, 8.6, 2.8 Hz, 1H), 2.21 (dt, *J* = 14.3, 10.1 Hz, 1H), 2.15 (ddd, *J* = 14.7, 7.1, 1.9 Hz, 1H), 2.08-2.03 (m, 1H), 1.99-1.76 (m, 6H), 1.74-1.61 (m, 2H), 1.14 (s, 18H) 1.04-1.01 (s, 21H). ^{13}C NMR (125 MHz, $CDCl_3$): δ 177.9, 1778.8, 71.9, 70.5, 68.8, 68.7, 42.0, 39.1, 39.0, 37.6, 37.0, 36.4, 34.6, 34.1, 33.9, 33.3, 27.5, 27.4, 24.6, 23.2, 18.5, 18.6, 12.8, 12.7. HRMS (ESI): calcd for $C_{21}H_{42}O_3NaSSi^+$ [M + Na]$^+$, 425.2516; found, 425.2513.

5-hydroxythiocan-3-yl pivalate (16).

5-((triisopropylsilyl)oxy)thiocan-3-yl pivalate (243 mg, 0.581 mmol, 1.0 equiv) was dissolved in THF (5.6 mL, anhydrous). The solution was cooled to 0 °C and tetrabutyammonium fluoride (TBAF) (1.70 mL of 1.0M solution in THF, 1.70 mmol, 2.93 equiv) was added dropwise to this solution. The reaction was then warmed to rt and stirred for 2 h. The reaction was evaporated to dryness, and the crude product was purified by silica gel chromatography (3:1 hexane/EtOAc) to give 118 mg of the desired product as a 1:1 mixture of two diastereomers (0.48 mmol, 83%). R_f = 0.2 in 3:1 hexane/EtOAc. ^{1}H NMR (600 MHz, $CDCl_3$): δ 5.07 (tdd, *J* = 8.1, 4.1, 1.5 Hz, 1H), 4.97-4.89 (m, 1H), 4.22-4.18 (m, 1H), 4.04-4.00 (m, 1H), 2.91- 2.78 (m, 3H), 2.77-2.68 (m, 3H), 2.67-2.56 (m, 3H), 2.34-2.22 (m, 2H), 2.08 (dd, *J* = 15.2, 5.6 Hz, 1H), 2.05-1.92 (m, 4H), 1.92-1.67 (m, 6H), 1.17 (s, 18H). ^{13}C NMR (150 MHz, $CDCl_3$): δ 178.2, 177.5, 71.5, 70.5, 68.0, 68.1, 39.2, 39.0, 38.7, 38.6, 36.1, 36.0, 33.9, 33.1, 32.7, 32.6, 27.1, 27.0, 24.1, 23.4. HRMS (ESI): calcd for $C_{12}H_{22}O_3NaS^+$ [M + Na]$^+$, 269.1182; found, 269.1184.

5-oxothiocan-3-yl pivalate (17).

5-hydroxythiocan-3-yl pivalate **16** (75 mg, 0.31 mmol, 1.0 equiv) was dissolved in dichloromethane (1.8 mL). To this solution, Dess-Martin periodinane (195 mg, 0.460 mmol, 1.50 equiv) was added, and the reaction mixture was stirred vigorously. A small amount of water (6 μL) was solvated in dichloromethane (6 mL) through pipetting, and the resulting wet dichloromethane was slowly added to the reaction mixture. The addition of the wet dichloromethane resulted in precipitation to form a cloudy mixture. After 30 min, the reaction was complete and dichloromethane was evaporated off. The reaction mixture was diluted in ether (10 mL), and the organics were washed with 1:1 sodium bicarbonate/10 % sodium thiosulfate aqueous solution (12 mL). The organic layer was washed with water (10 mL) and saturated sodium chloride solution (10 mL), dried with $MgSO_4$, decanted and evaporated to dryness. This procedure resulted in 55 mg of desired product (0.23 mmol, 74 %). R_f = 0.25 in 3:1 hexane/EtOAc. ^{1}H NMR (500 MHz, $CDCl_3$): δ 5.19 (tt, *J* = 10.2, 3.9 Hz, 1H), 2.93 (dd, *J* = 14.4, 3.7 Hz, 1H), 2.95-2.76 (m, 2H), 2.60 (dd, *J* = 12.7, 3.7 Hz, 1H), 2.45-2.53 (m, 3H), 2.41-2.36 (m, 1H), 2.22-2.12 (m, 2H), 1.14 (s, 9H). ^{13}C NMR (150 MHz, $CDCl_3$) δ 208.7, 177.3, 72.4, 45.0, 42.9, 38.7, 34.7, 32.7, 27.0. HRMS (ESI): calcd for $C_{12}H_{20}O_3NaS^+$ [M + Na]$^+$, 267.1025; found, 267.1027.

(E)-5-(((trifluoromethyl)sulfonyl)oxy)-3,6,7,8-tetrahydro-2H-thiocin-3-yl pivalate (18).

5-oxothiocan-3-yl pivalate **17** (20 mg, 0.082 mmol, 1.0 equiv) was dissolved in THF (2.8 mL, anhydrous), and the reaction was cooled to -78 °C. To this solution, sodium bis(trimethylsilyl)amide (NaHMDS) (82 μL of 2M in THF, 0.16 mmol, 2.0 equiv) was added. After stirring at -78 °C for 30 min, *N*-phenyl-bis(trifluoromethanesulfonimide) (33 mg, 0.092 mmol, 1.1 equiv) was added. After 20 additional minutes, the reaction was quenched with methanol (1 mL) and evaporated to dryness. The crude product was purified by silica gel chromatography (10:1 hexane/EtOAc) to give the desired product (20 mg, 0.053 mmol, 64 %). R_f = 0.7 in 3:1 hexane/EtOAc. ^{1}H NMR (600 MHz, $CDCl_3$): δ 5.88 (t, *J* = 8.6 Hz, 1H), 5.15-5.18 (m, 1H), 3.05-3.07 (m, 1H), 2.90-2.96 (m, 2H), 2.74 (dd, *J* = 15.2, 7.7 Hz, 1H), 2.62-2.70 (m, 2H), 2.57-2.61 (m, 1H), 2.46-2.51 (m, 1H), 1.16 (s, 9H). ^{13}C NMR (150 MHz, $CDCl_3$) δ 177.5, 149.4, 122.4, 118.4 (q, *J* = 318 Hz), 73.5, 38.7, 34.0, 33.7, 32.2, 30.0, 26.9. ^{19}F NMR (376 MHz, $CDCl_3$): δ -73.12 (s, 3F). HRMS (ESI): calcd for $C_{13}H_{19}O_5F_3NaS_2^+$ [M + Na]$^+$, 399.0518; found, 399.0519.

2,6,7,8-tetrahydro-4,5-didehydro-2*H*-thiocin-3-ol (thiaOCT, 8).

(E)-5-(((trifluoromethyl)sulfonyl)oxy)-3,6,7,8-tetrahydro-2H-thiocin-3-yl pivalate **18** (75 mg, 0.20 mmol, 1.0 equiv) was dissolved in THF (6.0 mL, anhydrous). The reaction mixture was cooled to 0 °C and lithium diisopropylamide (LDA) (0.2 M in THF, 2.1 mL, 0.42 mmol, 2.1 equiv) was added dropwise. The addition of LDA resulted in a color change from pale yellow to brown. After 10 min, the reaction was quenched with methanol (2 mL) and evaporated to dryness. The crude product was purified by silica gel chromatography (10:1 to 3:1 hexane/EtOAc) to yield the desired cyclooctyne **6** (15 mg, 0.11 mmol, 53 %). R_f = 0.3 in 3:1 hexane/EtOAc. ^{1}H NMR (400 MHz, $CDCl_3$): δ 4.37-

4.34 (m, 1H), 3.34 (dd, J = 10.0, 4.8 Hz, 1H), 3.28 (d, J = 7.0 Hz, 1H), 3.14 (dt, J = 9.4, 2.8 Hz, 1H), 2.92-2.88 (m, 1H), 2.76 (d, J = 10.0 Hz, 1H), 2.52-2.49 (m, 1H), 2.43-2.38 (m, 3H). ^{13}C NMR (150 MHz, $CDCl_3$) δ 91.2, 90.8, 71.5, 42.4, 41.1, 29.2, 23.5. HRMS (EI): calcd for $C_7H_{10}OS^+$ [M]$^+$, 142.0452; found, 142.0449; [M-H_2O]$^+$, 124; [M-CHO]$^+$, 113; [M-C_2H_3O]$^+$, 99; [M-C_5H_5O]$^+$, 61.

Figure S1

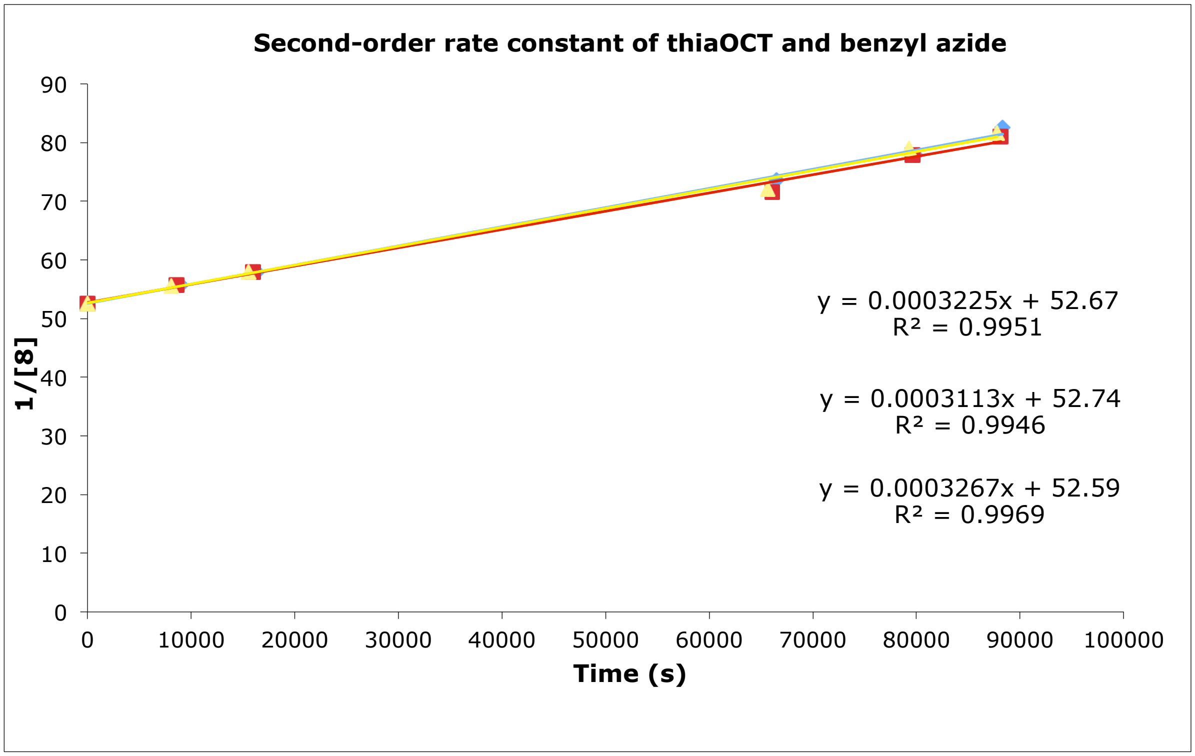

Figure S1.
The reaction of thiaOCT (**8**) and benzyl azide was monitored by ^{1}H NMR for 1500 min at rt. ThiaOCT and benzyl azide were separately dissolved in CD_3CN and mixed together in a 1: 1 ratio at a concentration of 19 mM. The percent conversion was calculated by the disappearance of thiaOCT and benzyl azide relative to the formation of product as determined by integration. The second-order rate constant was determined by plotting 1/[**8**] versus time. The plot was fit to a linear regression and the slope corresponds to the second-order rate constant. Shown are data from three replicate experiments. The three lines had an average slope of 0.00032 ± 7.7 x 10^{-6} $M^{-1}s^{-1}$.

Scheme S1. Synthesis of thiaDIFBO

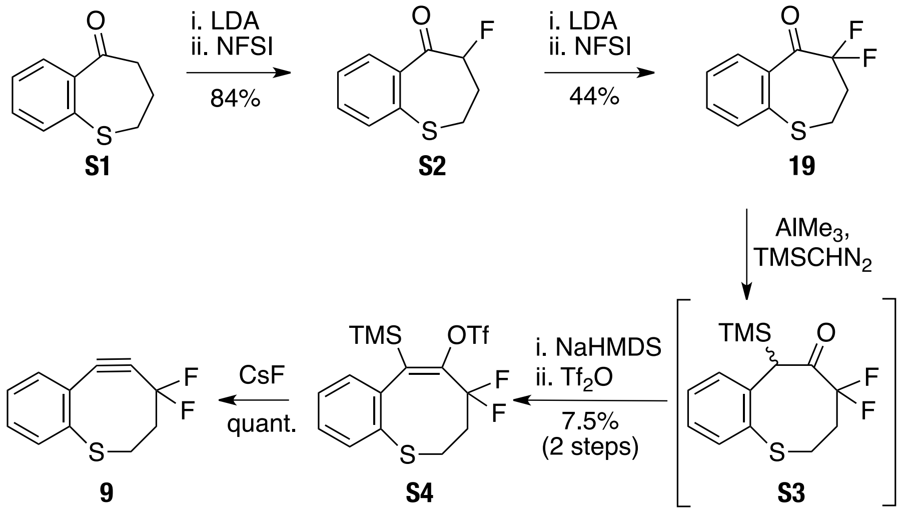

NFSI = *N*-fluorobenzenesulfonimide

S1 was sequentially difluorinated using LDA and *N*-fluorobenzenesulfonimide (NFSI) to yield **19**. This intermediate was homologated using $AlMe_3$ and $TMSCHN_2$ to yield α-silyl ketone **S3**. This step was far less efficient than achieved with the all-carbon cycloheptane analog, perhaps due to Lewis acid/base pairing of the sulfur atom and the trimethylaluminum. Due to the labile nature of the α-silyl ketone, crude **S3** was immediately converted to vinyl triflate **S4**, which was then quantitatively converted to thiaDIFBO (**9**) by treatment with CsF.

4-fluoro-3,4-dihydrobenzo[*b*]thiepin-5(2*H*)-one (S2).

A flame-dried flask was charged with 3,4-dihydrobenzo[*b*]thiepin-5(2*H*)-one **S1** (1.2 g, 6.6 mmol, 1 equiv). The flask was evacuated and backfilled with nitrogen twice. THF (33 mL, anhydrous) was added to the flask and the solution cooled to –78 ºC. LDA (4.0 mL of 2M solution in heptane/ THF/ ethylbenzene, 8.0 mmol, 1.2 equiv) was added and the solution warmed to 0 ºC and allowed to stir for 1 h. Separately, a dry flask was charged with *N*-fluorobenzenesulfonimide (NFSI) (2.7 g, 8.6 mmol, 1.3 equiv) and evacuated and backfilled with nitrogen twice. THF (30 mL, anhydrous) was added and the solution cooled to –78 ºC. The solution of base was slowly added to the NFSI solution over 12 min via syringe and the mixture was allowed to warm to rt over 30 min, at which point it was quenched with saturated ammonium chloride (50 mL). The organic layer was separated and the aqueous layer extracted with ethyl acetate (2 x 100 mL). The organic layers were combined, dried with sodium sulfate, filtered, and concentrated under reduced pressure. The crude oil was purified using silica gel chromatography (93:7 hexanes/EtOAc) to yield 1.1 g (5.6 mmol, 85%) of the desired product as a yellow oil. R_f = 0.48 in 9:1 hexane/EtOAc. ^{1}H NMR (300 MHz, $CDCl_3$): δ 8.02-7.98 (dd, *J* = 7.6,1.5 Hz, 1H), 7.47-7.44 (d, *J* = 7.8 Hz, 1H), 7.41-7.7.36 (td, *J* = 7.5, 1.5 Hz, 1H), 7.33-7.28 (t, *J* = 7.1 Hz, 1H), 5.81-5.76 (dd, *J* = 7.2, 9 Hz, 0.5 H), 5.65-5.60 (dd, *J*= 7.2, 9 Hz, 0.5H),

3.24-3.16 (m, 1H), 2.89-2.70 (m, 2H), 2.52-2.37 (m, 1H). ^{13}C NMR (130 MHz, $CDCl_3$): δ 197.5-197.4 (d, *J* = 16.6), 141.7, 134.9, 131.8, 131.1, 130.1, 126.2, 93.8-92.5 (d, *J* = 185), 36.4-36.3 (d, *J* = 24), 29.7 (d, *J* = 12). ^{19}F NMR (375 MHz, $CDCl_3$): δ -189.4 (dt, J_1 = 4, 45 Hz, 1F). HRMS (EI): calc for $C_{10}H_9FOS^+$ [M]$^+$, 196.0358; found, 196.0357; [M-CO]$^+$, 168; [M-CH_3S]$^+$, 149.

4,4-difluoro-3,4-dihydrobenzo[b]thiepin-5(2H)-one (19).

A flame-dried flask was charged with 4-fluoro-3,4-dihydrobenzo[*b*]thiepin-5(2*H*)-one **S2** (800 mg, 4.1 mmol, 1 equiv). The flask was evacuated and backfilled with nitrogen twice. THF (20 mL, anhydrous) was added to the flask and the solution cooled to –78 ºC. LDA (2.5 mL of 2 M solution in heptane/ THF/ ethylbenzene, 4.9 mmol, 1.2 equiv) was added and the solution warmed to 0 ºC and allowed to stir for 1 h. Separately, a dry flask was charged with NFSI (1.7 g, 5.3 mmol, 1.3 equiv) and evacuated and backfilled with nitrogen twice. THF (20 mL, anhydrous) was added and the solution cooled to –78 ºC. The solution of base was slowly added to the NFSI solution over 10 min via syringe and the reaction then allowed to warm to rt. Saturated ammonium chloride (40 mL) was added to the reaction followed by ethyl acetate (50 mL). The organic layer was separated and the aqueous layer extracted with ethyl acetate (2 x 50 mL). The organic layers were combined, dried over sodium sulfate, filtered, and concentrated under reduced pressure. The crude oil was purified using silica gel chromatography (93:7 hexanes/$_{EtOAc}$) to yield 390 mg (1.8 mmol, 45%) of a light yellow oil that solidified upon cooling. R_f = 0.55 in 9:1 hexane/EtOAc.^1H NMR (500 MHz, $CDCl_3$): δ 7.65-7.63 (d, *J* = 7.8, 1H), 7.39-7.30 (m, 2H), 7.26-7.24 (t, *J* = 8 Hz, 1H), 3.05-3.03 (t, *J* = 6 Hz, 2H), 2.76-2.68 (m, 2H). ^{13}C NMR (150 MHz, $CDCl_3$): δ 193, 138.5, 135, 132, 131.2, 130, 126.4, 118.6 (t, *J* = 243), 39.6 (t, *J* = 25), 28. ^{19}F NMR (375 MHz, $CDCl_3$): δ -99 (t, *J* = 19 Hz, 1F). HRMS (EI): calc for $C_{10}H_8F_2OS^+$ [M]$^+$, 214.0264; found, 214.0270; [M-CO]$^+$, 186; [M-CH_2S]$^+$, 168.

(*Z*)-4,4-difluoro-6-(trimethylsilyl)-3,4-dihydro-2*H*-benzo[*b*]thiocin-5-yl trifluoromethanesulfonate (S4).

A flame-dried flask was charged with 4,4-difluoro-3,4-dihydrobenzo[b]thiepin-5(2H)-one **19** (214 mg, 1 mmol, 1 equiv) and evacuated and backfilled with nitrogen twice. Dry dichloromethane (12.5 mL) was added and the solution was cooled to 0 ºC. Trimethylsilyl diazomethane ($TMSCHN_2$) (600 μL of 2M solution in dichloromethane, 1.2 mmol, 1.2 equiv) was added via syringe immediately followed by trimethylaluminum ($AlMe_3$) (600 μL of 2M solution in toluene, 1.2 mmol, 1.2 equiv). The reaction was stirred at 0 ºC for 10 min, at which point the reaction was quenched with saturated ammonium chloride (5 mL) followed by saturated Rochelle's salt (5 mL). Three times, dichloromethane (3 x 25 mL) was added and the organic layer was separated. The organic layers were combined, dried over sodium sulfate, filtered, and concentrated to an oil that still contained 4,4-difluoro-6-(trimethylsilyl)-3,4-dihydro-2*H*-benzo[*b*]thiocin-5(6*H*)-one (**S3**) and toluene was obtained.

The oil was transferred to a dry flask that was evacuated and backfilled with nitrogen twice. Dry THF (10 mL) was added and the reaction cooled to –78 ºC. NaHMDS (600 μL of 2M solution in THF, 1.2 mmol, 1.2 equiv) was added and the reaction stirred for 2 h at –78 ºC. Trifluoromethane sulfonic anhydride (200 μL, 1.2 mmol, 1.2 equiv) was then added and the reaction stirred for 1 h at –78 ºC. Methanol (1 mL) was added and the reaction was then allowed to warm to rt and concentrated. The oily solid was taken up in dichloromethane and filtered. The filtrate was concentrated and purified via HPLC on a 100 Å C_{18} column, (70% to 100% acetonitrile in water over 30 minutes). The desired product eluted at 17 minutes. Concentration of the desired fraction yielded 16 mg (0.037 mmol, 3.8%) of (*Z*)-4,4-difluoro-6-(trimethylsilyl)-3,4-dihydro-2*H*-benzo[*b*]thiocin-5-yl trifluoromethanesulfonate **S4** as a clear oil. R_f = 0.82 in 9:1 hexane/EtOAc. ^{1}H NMR (500 MHz, $CDCl_3$): δ 7.67-7.65 (d, *J* = 8, 1H), 7.40-7.37 (t, *J* = 7.5, 1H), 7.29-7.26 (m, 1H), 7.16-7.14 (d, *J* = 7.5 Hz, 1H), 3.00-2.96 (m, 1H), 2.83-2.76 (m, 1H), 2.35-2.23 (m, 1H), 2.12-2.05 (m, 1H), 0.019 (s, 9H). ^{13}C NMR (125 MHz, $CDCl_3$): δ 144.1(t, *J* = 30), 143 (d, *J* = 24), 136.6, 130.3, 129.3, 128.2, 126.5, 122.6, 120, 119.9, 117.5, 116, 34.2 (t, *J* = 26.3 Hz), 29.8 (t, *J* = 5 Hz), -1.3. ^{19}F NMR (375 MHz, $CDCl_3$): δ -70 (t, *J* = 19 Hz, 3F), -90 (dp, J_1 = 262, 22.5 Hz, 1H), -92 (bd, *J* = 274 Hz, 1H) . HRMS (EI): calc for $C_{15}H_{17}F_5O_3S_2Si^+$ [M]$^+$, 432.0309; found, 432.0316, [M-C O_3F_3S]$^+$, 283; [M-$C_7H_4O_3F_3S$]$^+$, 207.

4,4-difluoro-3,4-dihydro-5,6-didehydro-2*H*-benzo[*b*]thiocine (ThiaDIFBO, 9).

(*Z*)-4,4-difluoro-6-(trimethylsilyl)-3,4-dihydro-2*H*-benzo[*b*]thiocin-5-yl trifluoromethanesulfonate **S4** (16 mg, 0.037 mmol, 1 equiv) was dissolved in deuterated acetonitrile (1 mL) and cesium fluoride (34 mg, 0.22 mmol, 6 equiv) was added. The reaction was stirred for 1 h at rt. It was then filtered, and the filtrate concentrated and purified by silica gel chromatography (99:1 hexanes/ EtOAc) to yield 7.7 mg (0.036 mmol, 99%) of the desired product as a clear oil. R_f = 0.86 in 9:1 hexane/EtOAc.^{1}H NMR (600 MHz, CD_3CN): δ 7.56-7.54 (d, *J* = 9 Hz, 1H), 7.47-7.45 (d, *J* = 8 Hz, 1H), 7.42-7.39 (t, *J* = 7.2 Hz, 1H), 7.35-7.33 (t, *J* = 7.5 Hz, 1H), 3.17-3.16 (m, 2H), 2.86- 2.79 (m, 2H). ^{13}C NMR (125 MHz, CD_3CN): δ 148, 131.4, 130.9, 128.8, 127.4, 122.4, 120.6, 119.9, 106.3 (t, *J* = 9 Hz), 46.1 (t, *J* = 27.5 Hz), 28.8 (t, *J* = 3.8 Hz). ^{19}F NMR (375 MHz, CD_3CN): -86 (bt, 2F). HRMS (EI): calc for $C_{11}H_8F_2^+$ [M]$^+$, 210.0315; found, 210.0321, [M-C_2H_3]$^+$, 183; [M-CF_2]$^+$, 160.

Figure S2

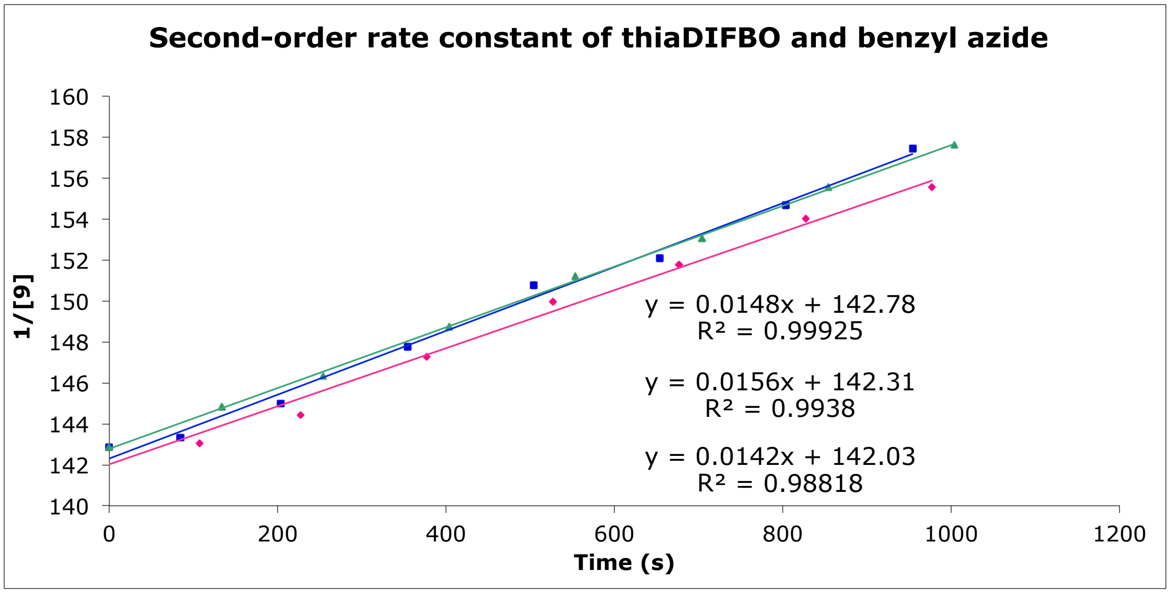

Figure S2.
The reaction of thiaDIFBO **9** and benzyl azide was monitored by ^{1}H NMR for 20 min at rt. ThiaDIFBO and benzyl azide were separately dissolved in CD_3CN and mixed together in a 1: 1 ratio at a concentration of 7 mM. The percent conversion was calculated by the disappearance of thiaDIFBO and benzyl azide relative to the formation of product as determined by integration. The triazole isomers were formed in approximately a 3:1 ratio and could be separated by HPLC (1.86:1 H_2O/ MeCN to 1:3 H_2O/ MeCN, 34.5 min, C_{18} column). The second-order rate constant was determined by plotting 1/[**9**] versus time. The plot was fit to a linear regression and the slope corresponds to the second-order rate constant. Shown are data from three replicate experiments. The three lines had an average slope of 0.015 ± 0.001 $M^{-1}s^{-1}$.

Scheme S2. Synthesis of TMTH

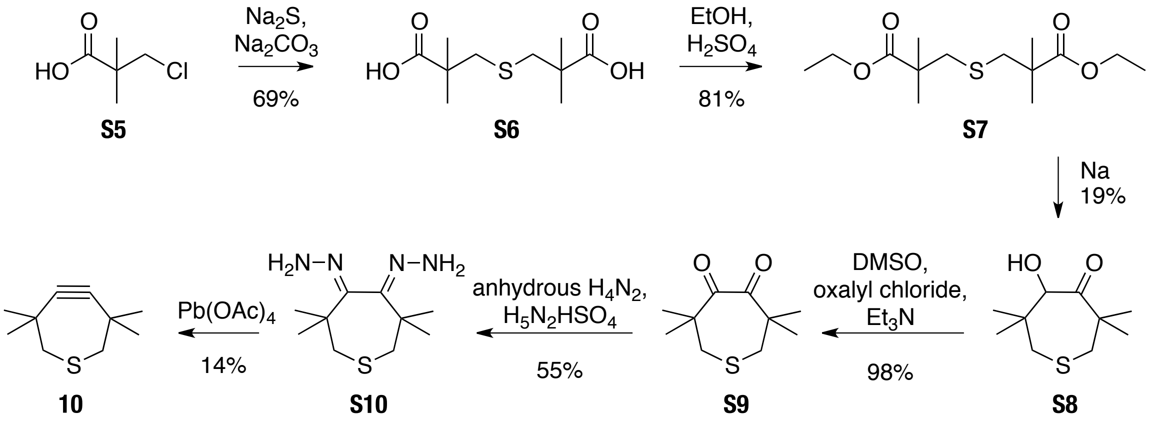

3,3'-thiobis(2,2-dimethylpropanoic acid) (S6).

Sodium carbonate (4.0 g, 37 mmol, 0.5 equiv) was dissolved in water (13 mL) and the solution was poured into a vessel containing chloropivalic acid **S5** (10 g, 74 mmol, 1 equiv). Once bubbling subsided, sodium sulfide nonahydrate (17.8 g, 185 mmol, 2.5 equiv) dissolved in water (25 mL) was added. The reaction was stirred for 72 h at rt. The pH was adjusted to 1 by addition of 50% sulfuric acid. The suspension was filtered and the solid dissolved in hot ethanol and filtered. The filtrate was concentrated and recrystallized out of hot water with about 10% ethanol. The solid was dried under high vacuum to yield 6.0 g (26 g, 69%) of a white powder. ^{1}H NMR (400 MHz, $CDCl_3$): δ 2.81 (s, 4H), 1.27 (s, 12H). HRMS (ESI): calc for $C_{10}H_{17}O_4S^-$ [M-H]$^-$, 233.0853; found, 233.0855. In agreement with reported spectral data.[3]

diethyl 3,3'-thiobis(2,2-dimethylpropanoate) (S7).

3,3'-thiobis(2,2-dimethylpropanoic acid) **S6** (4.0 g, 17 mmol, 1 equiv) was dissolved in ethanol (45 mL, 770 mmol, 45 equiv) and toluene (45 mL). Concentrated sulfuric acid (300 μL) was added and the flask was equipped with a Dean-Stark apparatus and heated at 90 °C overnight. The next day the reaction was cooled to rt and poured into a separatory funnel containing water (100 mL). The organic layer was separated and washed with saturated sodium bicarbonate (1 x 100 mL). The aqueous layers were combined and back extracted with ethyl acetate (1 x 300 mL). The organic layers were combined, dried over sodium sulfate, filtered, and concentrated. The crude oil was purified via silica gel chromatography (7:1 hexanes/EtOAc) to yield 4.0 g (13 mmol, 81%) of a clear oil. R_f = 0.65 in 9:1 hexane/EtOAc. ^{1}H NMR (500 MHz, $CDCl_3$): δ 4.16-4.10 (q, *J* = 7 Hz, 4H), 2.78 (s, 4H), 1.27-1.24 (t, *J* = 7 Hz, 6H), 1.23 (s, 12H). ^{13}C NMR (125 MHz, $CDCl_3$): δ 177, 61.1, 45.6, 44.6, 25.1, 14.6. FTMS (ESI): calc for $C_{14}H_{26}O_4NaS^+$ [M+Na]$^+$, 313.1444; found, 313.1442. In agreement with reported spectral data.[3]

5-hydroxy-3,3,6,6-tetramethylthiepan-4-one (S8).

In a flame-dried flask, sodium metal (390 mg, 17.2 mmol, 10 equiv) was added portionwise to boiling *m*-xylene (3.5 mL, anhydrous) while stirring vigorously. The reaction was then capped and positive nitrogen pressure was applied. Diethyl 3,3'-thiobis(2,2-dimethylpropanoate) **S7** (500 mg, 1.72 mmol, 1 equiv) in *m*-xylene (3.5 mL, anhydrous) was added via syringe over 2 h and the reaction was allowed to reflux for 0.5 h after addition. The reaction was then cooled to rt, filtered through celite and washed five times with toluene. The celite layer was carefully quenched with isopropanol (10 mL) and ethanol (2 mL). The filtrate was concentrated and filtered through a silica plug (8:1 EtOAc/MeOH). The combined eluent was concentrated. The resulting oil was then purified via silica gel chromatography (9:1 to 3:1 hexanes/EtOAc) to yield 65 mg (0.32 mmol, 19%) of light yellow crystals. R_f = 0.59 in 3:1 hexane/EtOAc. ^{1}H NMR (600 MHz,

CDCl$_3$): δ 4.17-4.16 (d, J = 6.6 Hz, 1H), 3.43-3.41 (d, J = 7.8 Hz, 1H), 2.73-2.71 (d, J = 12 Hz, 1H) 2.65- 2.58 (m, 2H), 2.44-2.41 (d, J = 14.4, 1H), 1.27 (s, 3H), 1.00 (s, 3H), 0.98 (s, 3H), 0.74 (s, 3H). ^{13}C NMR (150 MHz, CDCl$_3$): δ 211.4, 47.2, 44.4, 23.6. HRMS (EI): calc for $C_{10}H_{18}O_2S^+$ [M]$^+$, 202.1028; found, 202.1030. In agreement with reported spectral data.[3]

3,3,6,6-tetramethylthiepane-4,5-dione (S9).

A flame-dried flask was charged with oxalyl chloride (78 μL, 0.89 mmol, 2.5 equiv) and dichloromethane (1.8 mL, anhydrous). The solution was cooled to –78 ºC. Dimethylsulfoxide (110 μL, 1.60 mmol, 4.5 equiv, anhydrous) was added and the reaction stirred for 15 min at –78 ºC. 5-hydroxy-3,3,6,6-tetramethylthiepan-4-one **S8** (72 mg, 0.36 mmol, 1 equiv) in dichloromethane (1.8 mL, anhydrous) was added and the reaction was stirred for another 15 min at –78 ºC. Triethylamine (220 μL, 1.6 mmol, 4.5 equiv.) was added and the reaction stirred for 15 min at –78 ºC and warmed to 0 ºC for 25 min. Water (1 mL) was added and the organic layer separated. The aqueous layer was washed with dichloromethane (2 x 10 mL) and the organic layers were combined, dried over sodium sulfate, filtered, and concentrated under reduced pressure. The oil was purified on silica gel chromatography (95:5 hexanes/EtOAc) to yield 70 mg (0.35 mmol, 98%) of a light yellow oil. R_f = 0.58 in 9:1 hexane/EtOAc. ^{1}H NMR (400 MHz, CDCl$_3$): δ 2.58 (s, 4H), 1.27 (s, 12H). ^{13}C NMR (100 MHz, CDCl$_3$): δ 216.7, 72, 50.4, 47.4, 42.6, 42.4, 27.7, 27.3, 23.5, 19.2.HRMS (EI): calc for $C_{10}H_{16}O_2S^+$ [M]$^+$, 200.0871; found, 200.0876, [M-CO]$^+$, 172, [M-C_2O_2]$^+$, 144, [M-C_5H_7O]$^+$, 117. In agreement with reported spectral data.[4]

(1*Z*,1'*E*)-(3,3,6,6-tetramethylthiepane-4,5-diylidene)bis(hydrazine) (S10).

3,3,6,6-tetramethylthiepane-4,5-dione **S9** (150 mg, 0.75 mmol, 1 equiv) was combined with hydrazine sulfate (390 mg, 3.0 mmol, 4 equiv), anhydrous hydrazine (20 drops), and ethanol (15 drops) in ethylene glycol (1.5 mL) and stirred overnight at 150 ºC in a sealed vial. The following day, the reaction was cooled to rt, quenched with water (1 mL), and extracted three times with diethyl ether (3 x 2 mL). The organic layers were combined and concentrated to a white solid that was recrystallized out of hexanes to yield 100 mg (0.44 mmol, 59 %) of white crystals. R_f = 0.46 in 1:1 hexane/EtOAc. ^{1}H NMR (400 MHz, CDCl$_3$): δ 5.24 (s, 4H), 2.55-2.45 (q, J = 14.4 Hz, 4H), 1.33 (s, 6H), 1.20 (s, 6H). ^{13}C NMR (150 MHz, CDCl$_3$): δ 151.7, 45.3, 42, 44.6, 27.9, 26.8. FTMS (ESI): calc for $C_{14}H_{21}N_4S^+$ [M + H]$^+$, 229.1481; found, 229.1481. In agreement with reported spectral data.[4]

3, 3, 6, 6- tetramethylthiacycloheptyne (10).

A dry flask was charged with (1*Z*,1'*E*)-(3,3,6,6-tetramethylthiepane-4,5-diylidene)bis(hydrazine) **S10** (100 mg, 0.44 mmol, 1 equiv) and then evacuated and backfilled with nitrogen twice. Dichloromethane (1 mL, anhydrous) was then added, and the solution was cooled to 0 ºC. Lead tetraacetate (418 mg, 0.940 mmol, 2.15 equiv) was dissolved in dichloromethane (2.14 mL, anhydrous) and added dropwise over 1.5 h at 0

ºC. The reaction was quenched with aqueous sodium bicarbonate (1 mL) and extracted with dichloromethane (3 x 3 mL). The organic layers were combined, dried over sodium sulfate, filtered, and concentrated under reduced pressure. The crude product was used for kinetics experiments. Purification for analytical purposes was performed by fractional distillation using a Kugelrohr apparatus. 10 mg (0.059 mmol, 14 %) of the desired product were collected at 50 ºC , 8 mmHg as a light yellow oil. R_f = 0.75 in 19:1 hexane/diethyl ether. ^{1}H NMR (600 MHz, $CDCl_3$): δ 2.80 (s, 4H),1.22 (s, 12H). ^{13}C NMR (150 MHz, $CDCl_3$): δ 108.6, 52.7, 35, 26.2. HRMS (EI): calc for $C_{10}H_{16}S^+$ $[M]^+$, 168.0973; found, 168.0973, $[M-CH_3]^+$, 153, $[M-C_3H_7]^+$, 125, $[M-C_2H_5S]^+$, 107. In agreement with reported spectral data.[4]

Figure S3

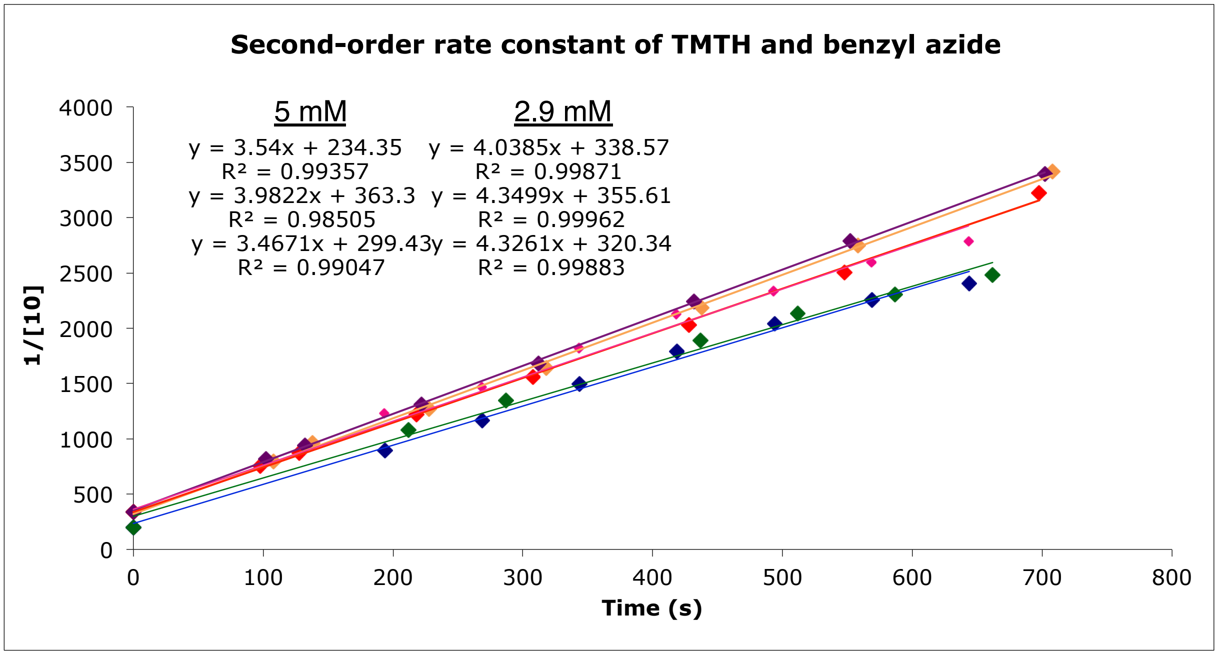

Figure S3.
The reaction of TMTH **10** and benzyl azide was monitored by ^{1}H NMR for 12 min at rt. TMTH and benzyl azide were separately dissolved in CD_3CN and mixed together in a 1: 1 ratio at concentration of 5 mM (blue, green, pink) or 2.9 mM (purple, orange, red) . The percent conversion was calculated by the disappearance of TMTH and benzyl azide relative to the formation of product as determined by integration. The second-order rate constant was determined by plotting 1/[**10**] versus time. The plot was fit to a linear regression and the slope corresponds to the second-order rate constant. Shown are data from three replicate experiments at each conentration. The six lines had an average slope of 4.0 ± 0.4 $M^{-1}s^{-1}$.

Figure S4

a)

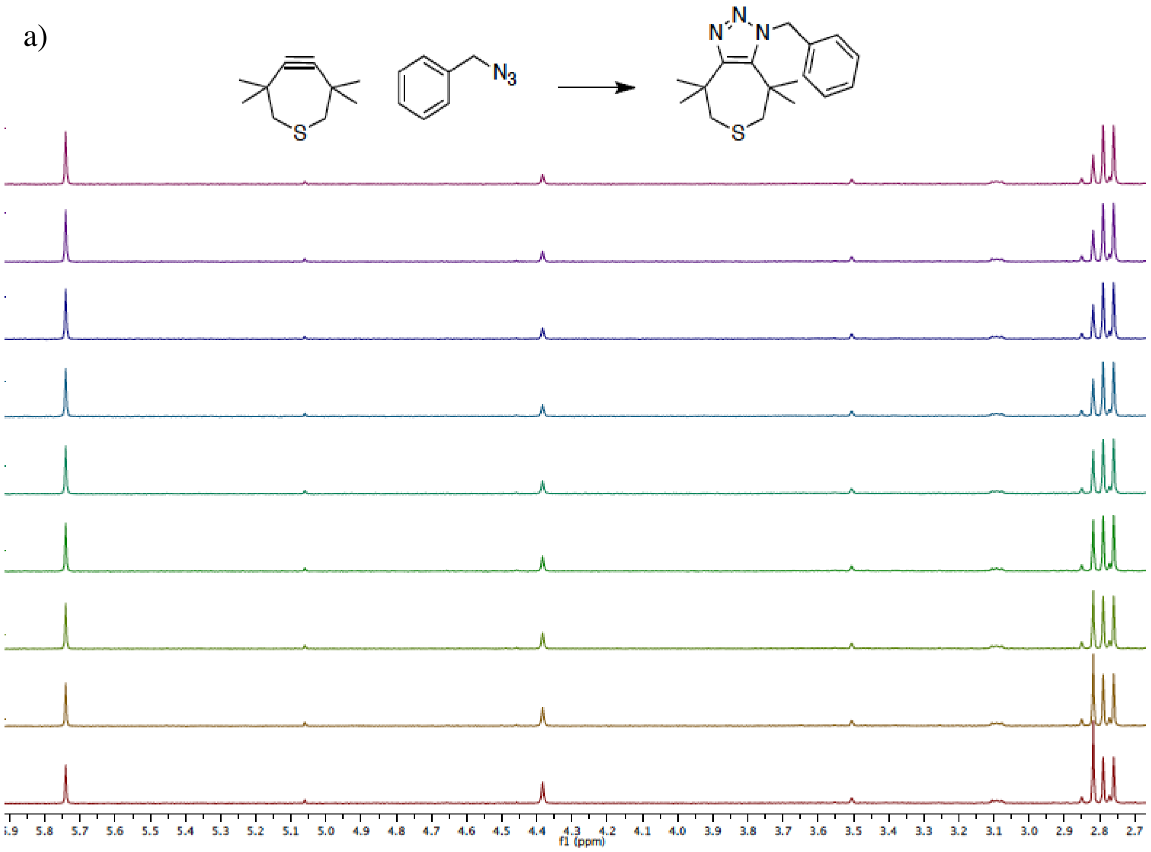
N
N
N
N3
S
S
5.9
5.8
5.7
5.6
5.5
5.4
5.3
5.2
5.1
5.0
4.9
4.8
4.7
4.6
4.5
4.4
4.3
4.2
4.1
4.0
3.9
3.8
3.7
3.6
3.5
3.4
3.3
3.2
3.1
3.0
2.9
2.8
2.7
f1 (ppm)

b)

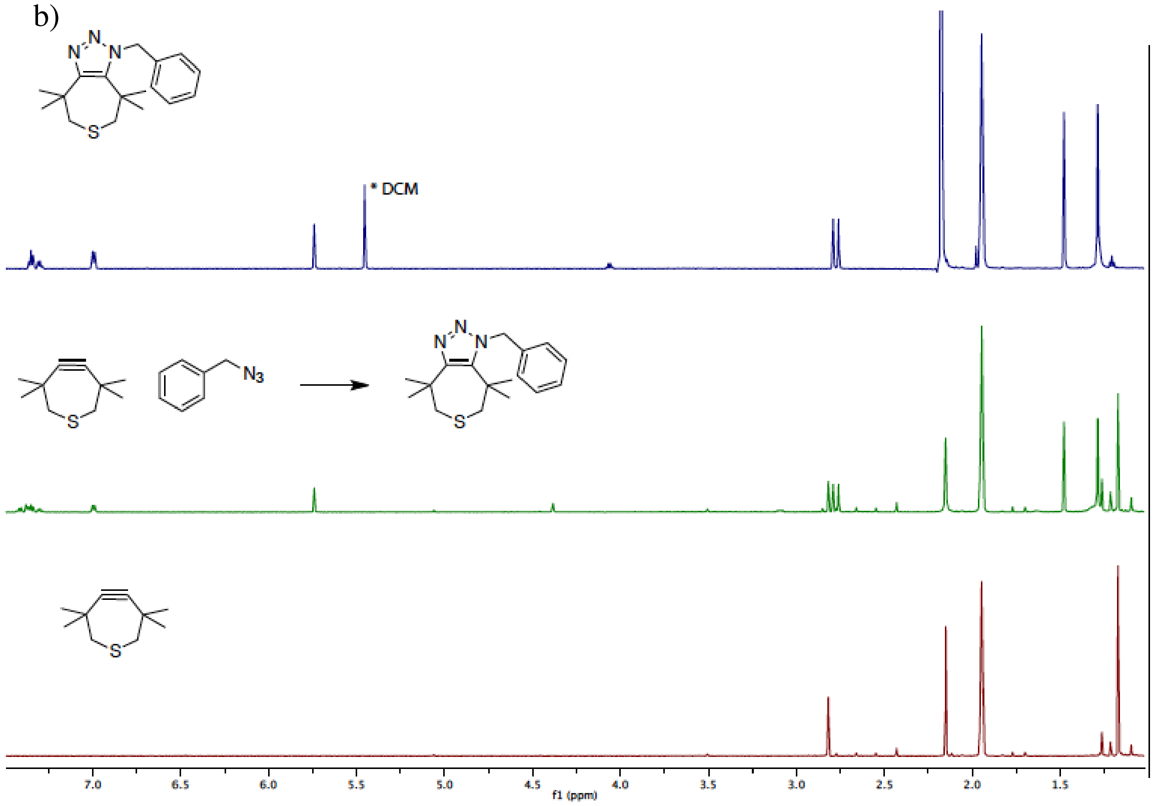
N
N
N
S
* DCM
N3
S
7.0
6.5
6.0
5.5
5.0
4.5
4.0
3.5
3.0
2.5
2.0
1.5
f1 (ppm)

Figure S4.
a) 30 s interval timepoints of NMR kinetics measurements of the reaction between 2.9 mM azide and 2.9 mM **10** in CD_3CN starting at 1.63 min through 5.63 min. (Measurements were taken until 15 min from mixing of starting materials.) The singlet at 5.75 ppm corresponds to the triazole benzylic protons, the singlet at 4.39 ppm corresponds to the azide benzylic protons, the singlet at 2.81ppm corresponds to the methylene protons on TMTH, and the doublet at 2.78 ppm corresponds to the methylene protons on the product. b) Direct comparisons of **10** (bottom), the reaction in progress at 2.63 min (middle) and a purified sample of triazole product (top). Peak at 1.9 ppm is CD_3CN and at 2.15 ppm is H_2O. At 16.13 min, the NMR spectrum shows 84% conversion to product. To obtain pure triazole sample, the kinetics samples were combined after each had been allowed to react for ~16 min. After concentration, the crude mixture was purified by silica gel chromatography (hexanes to 3:1 hexanes/EtOAc) resulting in pure triazole product in 38% isolated yield.

Scheme S3. Synthesis of **11**

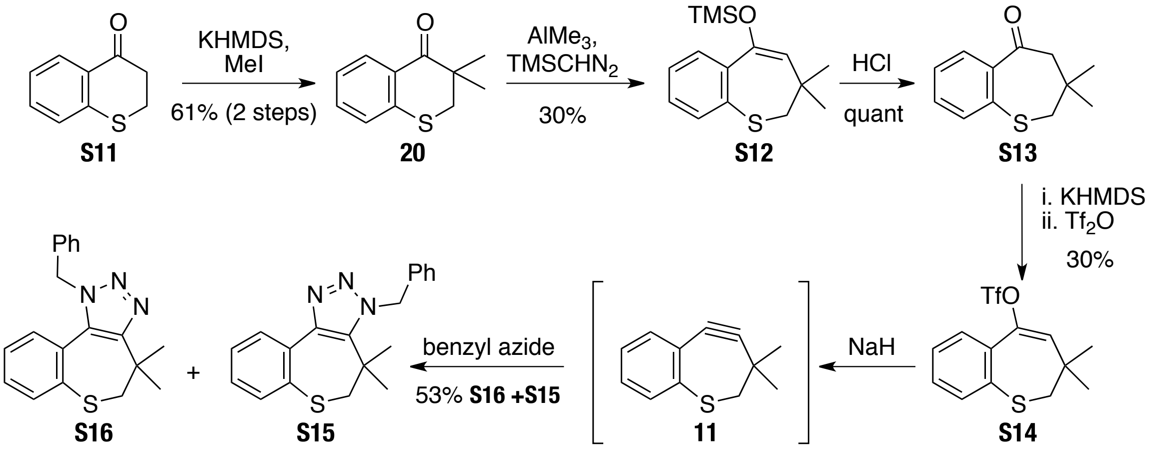

As with DIFBO and thiaDIFBO, the synthesis of **11** proceeded through a key ring expansion step (Scheme 3). Compound **S11** was dimethylated by treatment with KHMDS and methyl iodide to produce **20**. Homologation of **20** was performed using $AlMe_3$ and $TMSCHN_2$, producing the unexpected silyl enol ether **S12**, which was readily converted to ketone **S13** upon treatment with acid. Compound **S13** was then treated with KHMDS and trifluoromethane sulfonic anhydride to form vinyl triflate **S14**. Attempts to eliminate the triflate using LDA or hexamethyldisilylamide bases gave no reaction, perhaps due to unfavorable steric interactions between these large bases and the *gem*-dimethyl group. However, treatment of **S14** with NaH in the presence of benzyl azide gave triazole cycloadducts **S15** and **S16**, suggesting that **11** was formed *in situ*.

3-methylthiochroman-4-one.

THF (5.5 mL, anhydrous) and LiHMDS (3.61 mL of 1 M solution in THF, 3.61 mmol, 1.2 equiv) were combined and cooled to -78 ºC. Thiochromanone **S11** (502 mg, 3.05 mmol, 1.0 equiv) was dissolved in THF (2.0 mL, anhydrous) and added to the solution of base over 1 h at -78 ºC. After stirring for an additional hour at -78 ºC, MeI (0.93 mL, 15 mmol, 4.9 equiv) was added and the mixture was warmed to rt over 3 h at which point, the reaction was quenched with MeOH (2 mL) and evaporated to dryness. Silica gel chromatography (35:1 hexanes/ EtOAc) resulted in pure desired compound (400 mg, 2.24 mmol, 73%). R_f = 0.45 in 6:1 hexane/ethyl acetate. ^{1}H NMR (600 MHz, $CDCl_3$): δ 8.05 (dd, *J* = 7.2, 1.0, 1H), 7.33-7.30 (m, 1H), 7.19 (dd, *J* = 7.9, 0.7 Hz, 1H), 7.13-7.10 (m, 1H), 3.09 (s, 1H), 3.08 (d, *J* = 3.1 Hz, 1H), 2.88 (dp, *J* = 8.7, 6.8 Hz, 1H), 1.30 (d, *J* = 6.8 Hz, 3H). ^{13}C NMR (150 MHz, $CDCl_3$): δ 196.5, 141.9, 133.0, 130.5, 129.6, 127.4, 124.9, 42.2, 33.1, 15.1. HRMS (EI): Calcd. for $C_{10}H_{10}OS^+$ $[M]^+$ 178.0452, found 178.0452; $[M-C_2H_2O]^+$, 136; $[M-C_4H_6O]^+$, 108.

3,3-dimethylthiochroman-4-one (20).

THF (1.0 mL, anhydrous) and LiHMDS (0.96 mL of 1 M solution in THF, 0.96 mmol, 1.2 equiv) were combined and cooled to -78 ºC. 3-methylthiochroman-4-one (144 mg, 0.809 mmol, 1.0 equiv) was dissolved in THF (1.0 mL, anhydrous) and added to the solution of base over 1 h at -78 ºC. After stirring for an additional hour at -78 ºC, MeI (0.25 mL, 4.0 mmol, 4.9 equiv) was added and the mixture was warmed to rt over 3 h, at which point the reaction was quenched with MeOH (1 mL) and evaporated to dryness. Silica gel chromatography (40:1 hexanes/ EtOAc) resulted in pure **20** (129 mg, 0.672 mmol, 83%). R_f = 0.55 in 6:1 hexane/ethyl acetate. ^{1}H NMR (500 MHz, $CDCl_3$): δ 8.08 (dd, *J* = 8.0, 1.2 Hz, 1H), 7.33 (ddd, *J* = 8.0, 7.2, 1.5 Hz, 1H), 7.20 (dd, *J* = 8.0, 0.8 Hz, 1H), 7.14 (ddd, *J* = 8.2, 7.2, 1.2 Hz, 1H), 3.07 (s, 2H), 1.32 (s, 6H). ^{13}C NMR (125 MHz, $CDCl_3$): δ 198.5, 141.6, 132.9, 130.3, 129.7, 127.3, 124.9, 41.1, 39.3, 23.7. HRMS (EI): Calcd. for $C_{11}H_{12}OS^+$ $[M]^+$ 192.0609, found 192.0604; $[M-C_3H_4O]^+$, 136; $[M-C_5H_8O]^+$, 108.

(3,3-dimethyl-2,3-dihydrobenzo[*b*]thiepin-5-yloxy)trimethylsilane (S12).

3,3-dimethylthiochroman-4-one **20** (365 mg, 1.90 mmol, 1.0 equiv) was dissolved in CH_2Cl_2 (10 mL, anhydrous) and cooled to -78 ºC. $AlMe_3$ (1.14 mL of 2M solution in toluene, 2.28 mmol, 1.2 equiv) was added and the solution was stirred for 15 min at which point, $TMSCHN_2$ (1.14 mL of 2M solution in CH_2Cl_2, 2.28 mmol, 1.2 equiv) was added. The solution was warmed to rt overnight. The mixture was quenched with aqueous Rochelle's salt (5 mL) and stirred until two layers formed. The quenched solution was extracted with dichloromethane (3 x 10 mL). The organic layers were combined, dried with $MgSO_4$, decanted, and evaporated to dryness. Silica gel chromatography (150:1 hexanes/ EtOAc) resulted in pure **S12** (160 mg, 0.576 mmol, 30%). R_f = 0.85 in 6:1 hexane/ethyl acetate. ^{1}H NMR (600 MHz, $CDCl_3$): δ 7.38 (d, *J* =

7.6 Hz, 1H), 7.14-7.11 (m, 2H), 7.00 (t, *J* = 7.1 Hz, 1H), 5.81 (s, 1H), 2.77 (s, 2H), 1.28 (s, 6H), 0.30 (s, 9H). ^{13}C NMR (125 MHz, $CDCl_3$): δ 162.3, 137.4, 137.1, 131.5, 131.0, 126.6, 124.8, 110.1, 46.1, 44.2, 28.0, 0.8. HRMS (EI): Calcd. for $C_{15}H_{22}OSSi^+$ $[M]^+$ 278.1161, found 278.1163; $[M\text{-}C_4H_8]^+$, 222; $[M\text{-}C_6H_{12}Si]^+$, 165; $[M\text{-}C_7H_{14}OSi]^+$, 136; $[M\text{-}C_9H_{18}OSi]^+$, 108.

3,3-dimethyl-3,4-dihydrobenzo[*b*]thiepin-5(*2H*)-one (S13).

Silyl enol ether **S12** (90 mg, 0.32 mmol, 1 equiv) was dissolved in MeOH and 12M HCl (1 drop) was added. The mixture was stirred for 30 min at rt and then quenched with saturated sodium bicarbonate (until bubbling ceased). The MeOH was removed by rotary evaporation and the resulting aqueous solution was extracted with dichloromethane (3 x 10 mL). The organic layers were combined, dried with $MgSO_4$, decanted, and evaporated to dryness. This procedure resulted in pure ketone **S13** (65 mg, 0.31 mol, 97% yield). R_f = 0.5 in 4:1 hexane/ethyl acetate. ^{1}H NMR (400 MHz, $CDCl_3$): δ 7.46 (dd, *J* = 7.5, 1.1 Hz, 1H), 7.29 (d, *J* = 7.3 Hz, 1H), 7.20 (td, *J* = 7.5, 1.4 Hz, 1H), 7.14 (td, *J* = 7.5, 1.4 Hz, 1H), 4.03 (s, 2H), 2.83 (s, 2H), 1.32 (s, 6H). ^{13}C NMR (150 MHz, $CDCl_3$): δ 209.3, 138.2, 136.8, 132.7, 130.6, 128.6, 127.5, 49.3, 47.2, 46.2, 24.3. HRMS (EI): Calcd. for $C_{12}H_{14}OS^+$ $[M]^+$ 206.0765, found 206.0764

3,3-dimethyl-2,3-dihydrobenzo[*b*]thiepin-5-yl trifluoromethanesulfonate (S14).

Ketone **S13** (62 mg, 0.30 mol, 1 equiv.) was dissolved in THF (4.5 mL, anhydrous) and cooled to -78 °C. KHMDS (0.72 mL of 0.5 M solution in toluene, 0.36 mmol, 1.2 equiv) was added and the solution was stirred for 2 h at -78 °C at which point trifluoromethane sulfonic anhydride (70 μL, 0.42 mmol, 1.4 equiv) was added and the reaction was warmed to 0 °C over 2 h. The reaction mixture was quenched with MeOH (1 mL) and evaporated to dryness. Silica gel chromatography with hexanes/ethyl acetate (150:1) resulted in pure **S14** (66 mg, 0.20 mmol, 66%). R_f = 0.85 in 4:1 hexane/ethyl acetate. ^{1}H NMR (500 MHz, $CDCl_3$): δ 7.47 (dd, *J* = 7.6, 1.4 Hz, 1H), 7.30-7.24 (m, 2H), 7.20 (td, *J* = 7.4, 1.7 Hz, 1H), 6.62 (s, 1H), 2.86 (s, 2H), 1.42 (s, 6H). ^{13}C NMR (150 MHz, $CDCl_3$): δ 158.3, 139.2, 133.9, 132.9, 131.5, 128.2, 127.2, 122.0, 118.7 (q, *J* = 320 Hz), 45.5, 44.5, 27.3. ^{19}F NMR (565 MHz, $CDCl_3$): δ -75.63. HRMS (EI): Calcd. for $C_{13}H_{13}O_3S_2F_3^+$ $[M]^+$ 338.0258, found 338.0262; $[M\text{-}C_4H_8]^+$, 150; $[M\text{-}C_5H_8O]^+$, 122.

Triazole products (S15 and **S16**).

Vinyl triflate **S14** (10 mg, 0.030 mmol, 1.0 equiv.) was dissolved in THF (1.0 mL, anhydrous). NaH (10 mg, 60% in mineral oil, 0.26 mmol, 8.7 equiv.) was added to this solution followed by benzyl azide (15 μL, 0.12 mmol, 4.0 equiv.). The mixture was allowed to stir overnight. The following day the reaction was quenched with MeOH (0.25 mL) and evaporated to dryness. The crude reaction mixture was purified by silica gel chromatography (6:1, 4:1, 1:1 hexanes/ethyl acetate). This procedure resulted in 5 mg of pure **S15** and **S16** (0.016 mmol, 53%) in a 1:0.6 ratio. R_f = 0.25 in 4:1 hexane/ethyl acetate. ^{1}H NMR (600 MHz, MeOD): δ 8.21 (d, *J* = 7.8 Hz, 1H), 7.60 (d, *J* = 7.8 Hz,

1H’), 7.56 (d, J = 7.8 Hz, 1H’), 7.52 (d, J = 7.7 Hz, 1H), 7.39-7.35 (m, 3H, 2H’), 7.31-7.22 (m, 2H, 3H’), 7.08 (d, J = 7.7 Hz, 2H), 6.96 (d, J = 6.8 Hz, 2H’), 5.92 (s, 2H), 5.69 (s, 2H’), 2.96 (s, 2H), 2.90 (s, 2H’), 1.50 (s, 6H’), 1.46 (s, 6H). ^{13}C NMR (150 MHz, MeOD): δ 154.0, 143.5, 143.0, 142.1, 139.5, 138.2, 137.4, 134.8, 134.5, 133.1, 132.4, 131.5, 131.1, 130.2, 130.0, 129.9, 129.9, 129.1, 129.0, 128.8, 128.6, 128.6, 127.8, 127.54, 127.52, 55.0, 54.1, 51.6, 51.2, 49.7, 39.5, 39.0, 30.4, 28.4. HRMS (ESI): Calcd. for $C_{19}H_{20}N_3S^+$ [M+H]$^+$ 322.1372, found 322.1374.

Figure S5

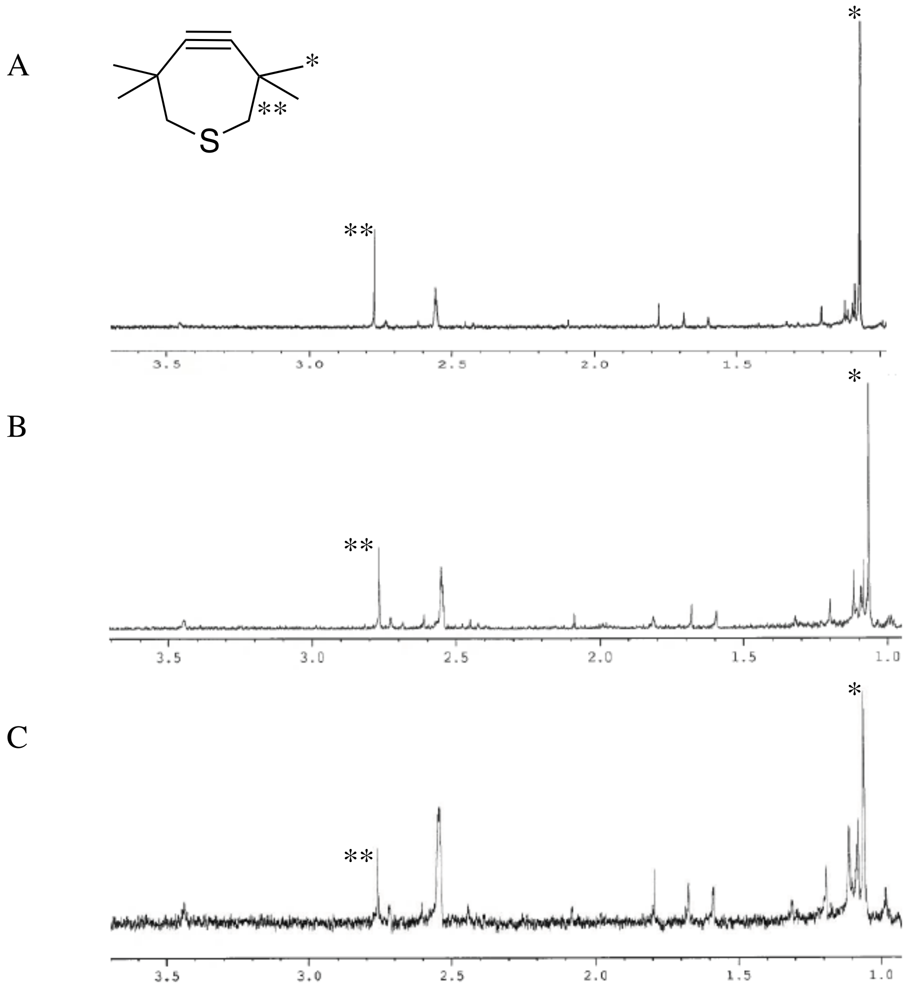

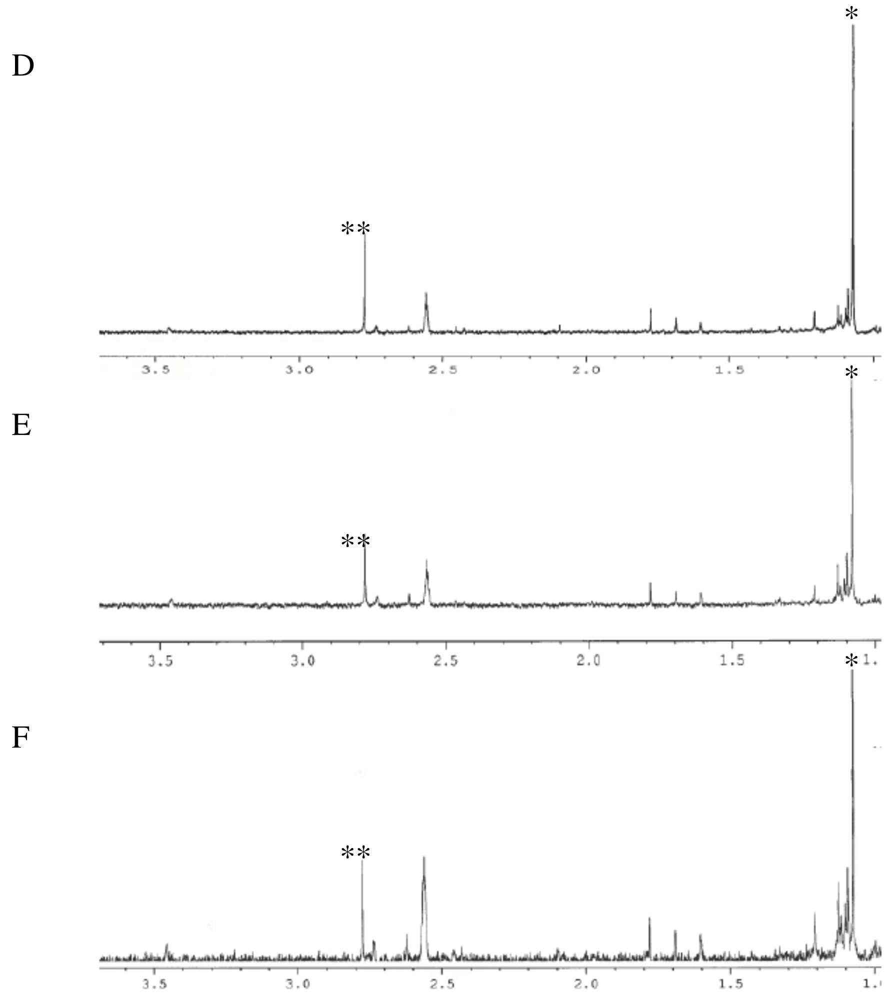

Figure S5.
A 100 mM solution of TMTH (**10**) in d_6-DMSO was diluted in D_2O (A, B, C, D) or deuterated PBS (a solution of PBS was lyophilized and the residue dissolved in D_2O) (E, F) to a final concentration of 1 mM. The solution was allowed to sit at room temperature and an NMR taken (Bruker AV-500) at 10 minutes (A, D), 48 h (B, E), and 144 h (C, F). After 48 h, the amount of TMTH has decreased in both samples. The compound still persists after 144 h, though significant amounts are lost.

Figure S6

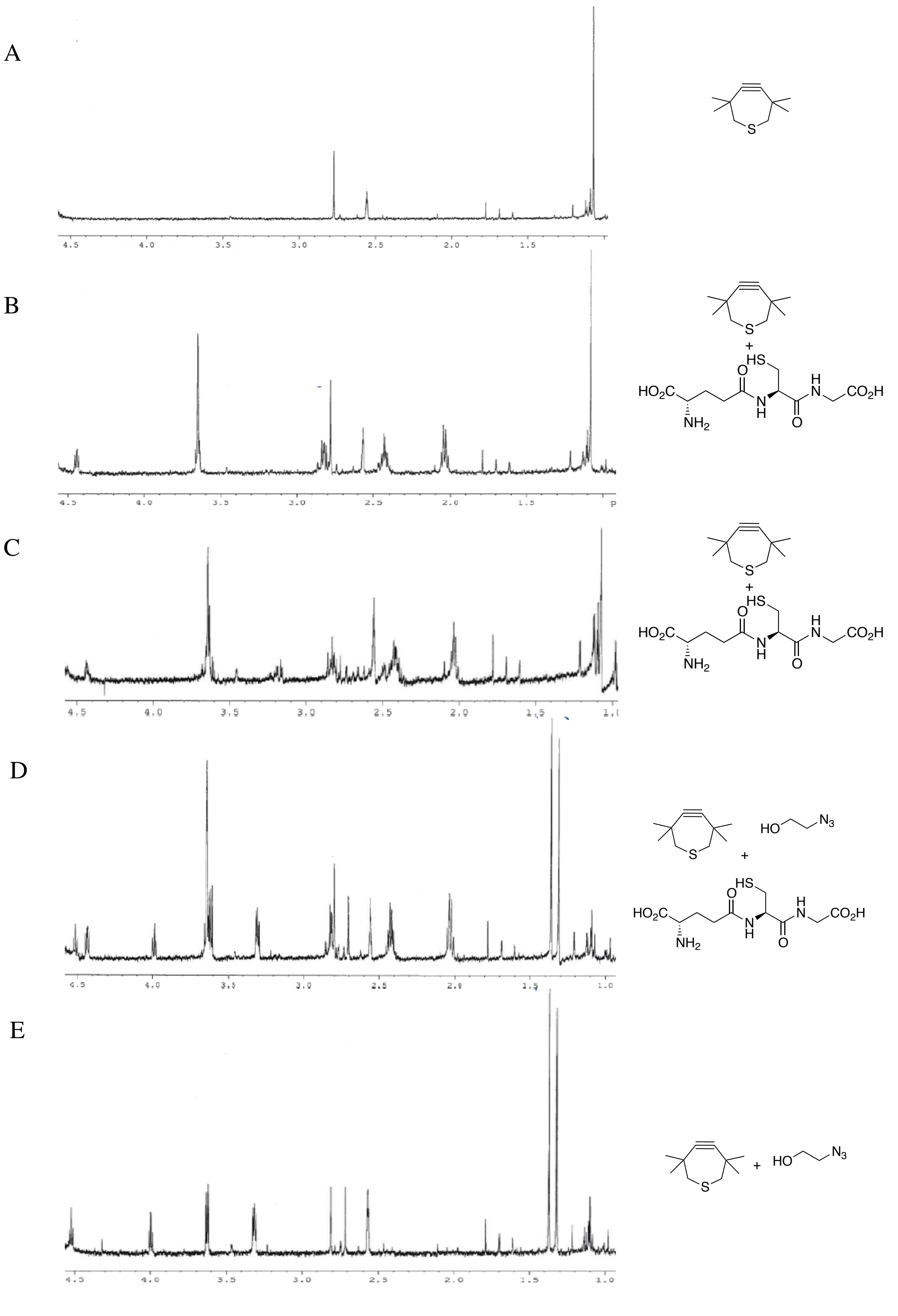

Figure S6.
TMTH reacts more readily with 2-azidoethanol than glutathione in PBS. All NMR spectra were taken on Bruker AV-500 or Bruker AV-600. A) A 1 mM solution of TMTH in deuterated PBS B) A solution of 1 mM TMTH and 1 mM glutathione in deuterated PBS was allowed to react for 10 min. No reaction is observed. C) A solution of 1 mM TMTH and 1 mM glutathione in deuterated PBS was allowed to react for 26 h. No starting TMTH is present, and the product methyl peaks appear at ~1.1 ppm. D) TMTH was added to a final concentration of 1 mM TMTH to a solution of 1 mM 2-azidoethanol and 1 mM glutathione in deuterated PBS. The NMR taken after 10 min indicates no remaining TMTH and methyl peaks corresponding to those of the cycloadduct with 2-azidoethanol. E) A solution of 1 mM TMTH and 1 mM azidoethanol in deuterated PBS was allowed to react for 10 min. No starting TMTH is present, and the product methyl peaks appear at ~ 1.3 – 1.4 ppm.

Western Blot Competition Experiments
Western blot analysis was performed on Jurkat cell lysates. Jurkat cells were incubated in the described media containing 25 μM $Ac_4GalNAz$ or DMSO vehicle for 3 days. They were then washed in Dulbecco's modified PBS and lysed in 1% NP-40, NaCl (150 mM), Tris pH 7.4 with protease inhibitors. The cell lysate was then sonicated and cleared by centrifugation. Protein concentration was normalized using a BCA assay.

Figure 2: TMTH (**10**) was added at varying concentrations for 1.5 hours. Phosphine-FLAG (500 μM final concentration) was added to the protein solutions and the vials agitated overnight.

Figure S8: Phosphine-FLAG (500 μM final concentration) was added to the protein solutions at 0^0 C. TMTH (**10**) was then added at varying concentrations and the vials agitated at room temperature overnight.

The following day, 4X loading buffer was added and the proteins were separated on a 4-12% Bis-Tris gradient SDS-PAGE gel at 140 V (Bio-Rad, Criterion system). They were then electroblotted onto nitrocellulose, blocked in 5% bovine serum albumin (BSA, Sigma) in Tris-buffered saline with Tween (TBST,10 mM Tris pH 8.0, 150 mM NaCl, 0.1% Tween-20), and treated with anti-FLAG (M2, Sigma, 1:1000 dilution in 5% BSA in TBST from stock) overnight at 4 ºC. The blots were washed with TBST three times for ten min then treated with anti-mouse κ light chain-HRP-conjugated secondary antibody (Southern Biotech, 1:5000 dilution in 5% BSA in TBST). The blots were again washed with TBST three times for ten min and analyzed by standard enhanced chemiluminescence immunoblotting methods (Pierce).
The blots were stored at 4 ºC. India ink staining was performed by washing the blots copiously with deionized water 3x for ten min then TBST for ten min. The blot was then incubated in 1:1000 dilution of India ink in TBST for 1 h then washed for 30 s with water and ten min with TBS.

Figure S7

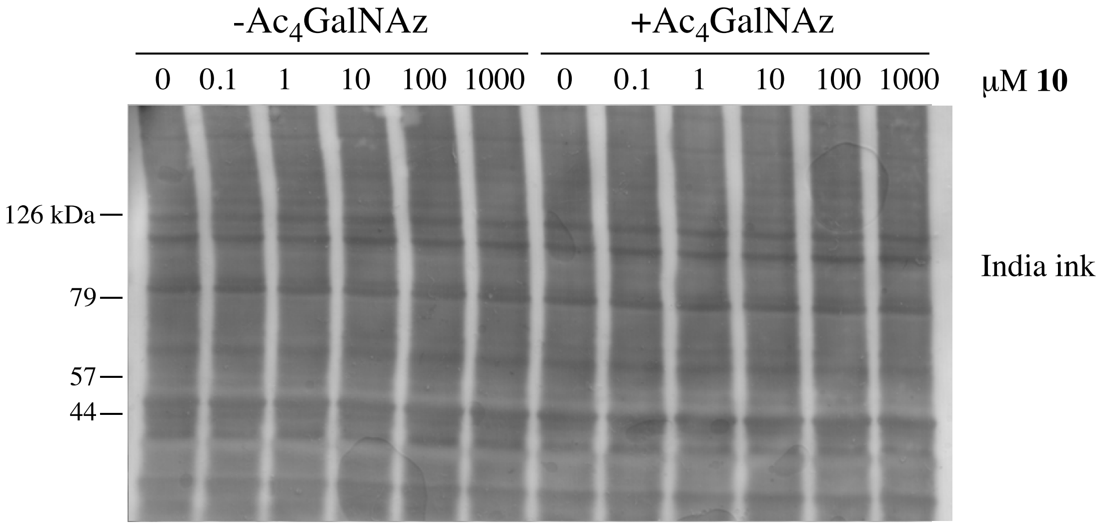

Figure S7.
India ink staining of the western blot shown in Figure 2

Figure S8

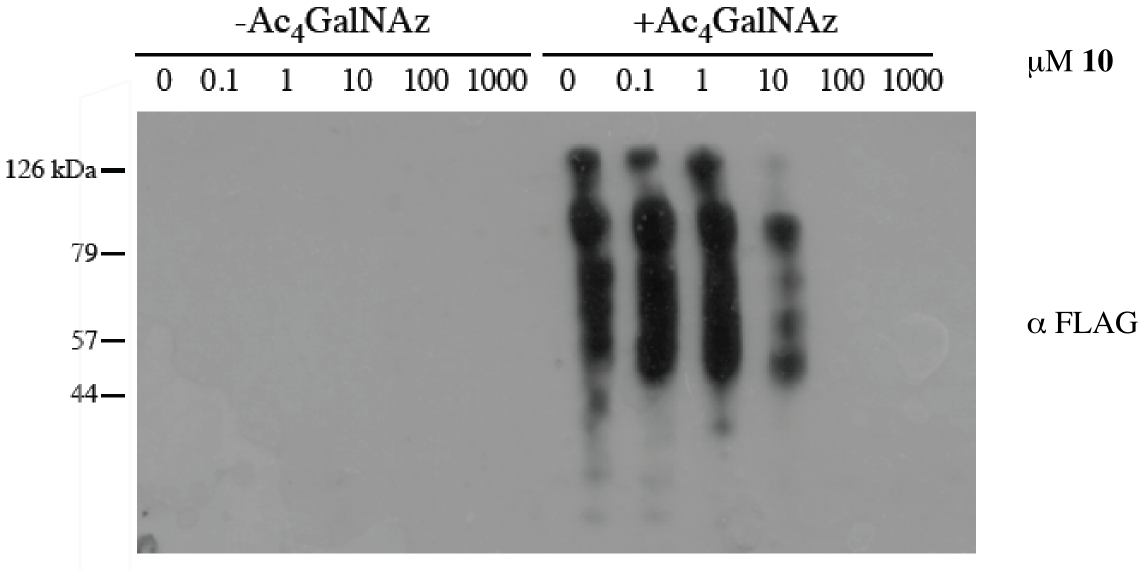

Figure S8.
Simulatneous treatment Ac_4GalNAz-labeled cells with TMTH and phosphine-FLAG inhibits phosphine- FLAG dependent labeling. Jurkat cells were incubated with Ac_4-GalNAz (50μM) or vehicle for 3 d and then lysed. The lysates were treatedwith phos - phine-FLAG (500μM) at 0° C then TMTH (concentrations from 1 nM to 1 mM) and allowed to warm to room temperature and incubate overnight. The lysates were analyzed

by Western blot using an anti-FLAG antibody then a secondary antibody conjugated to horse radish peroxidase.

Figure S9

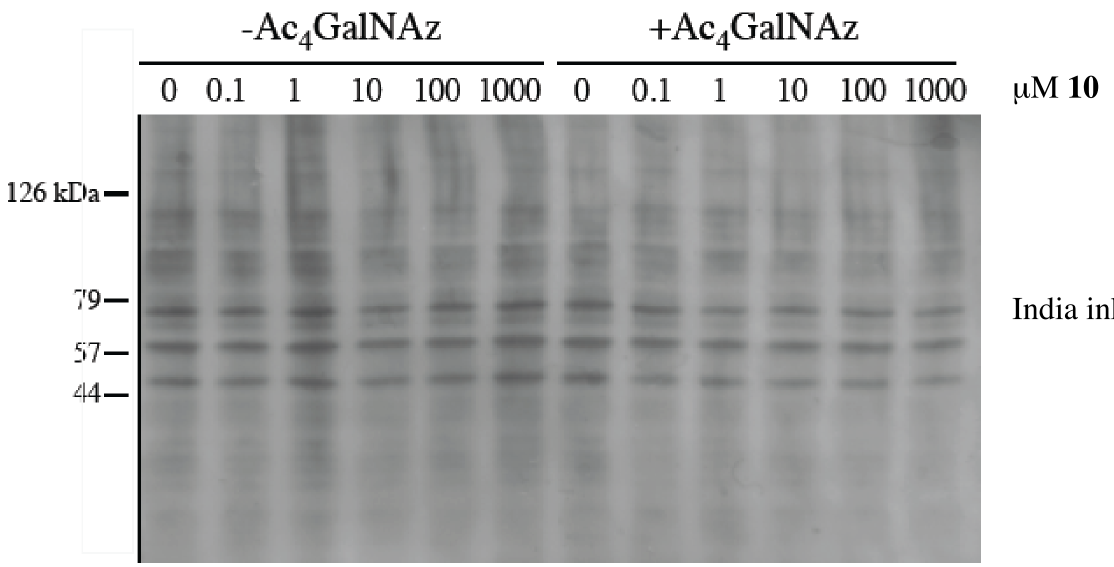

Figure S9.
India ink staining of the western blot shown in Figure S8.

Barstar Expression and Purification

A plasmid (pQE30-Barstar) containing the *Bacillus amyloliquefaciens* protein Barstar as a 6xHis fusion and with two point mutations for improved stability (Cys53Ala and Cys95Ala) in a pQE30 expression vector was obtained from D. Tirrell (California Insitiue of Technology). For incorporation of the unnatural amino acid azidohomoalanine (AHA), the *E. coli* methionine auxotrophic strain M15-MA was also obtained from D. Tirrell.

To generate both the wild-type Barstar protein (Barstar-MET) and the AHA containing Barstar protein (Barstar-AHA) we followed an expression protocol similar to that reported by *Beatty* et. al.[5] Briefly, the pQE30-Barstar plasmid was transformed into M15-MA cells and individual transformants were used to inoculate 5 mL of M9 minimal media supplemented with 0.04 mg/mL of each of the 20 amino acids, 1mM $MgSO_4$, 5 μg/mL thiamine, 0.4% glucose, 200 μg/mL ampicillin and 35 μg/mL kanamycin (i.e., M9 complete media with 20 amino acids). After an overnight incubation at 37 °C with shaking, 1 mL was transferred to 50 mL of M9 complete media with 20 amino acids. After reaching an OD_{600} of 1.0, the cells were pelleted (6100g for 10 min at 4 °C) and washed three times with a sterile solution of ice-cold 0.9% (w/v) NaCl. The culture was resuspended in M9 complete medium with 19 amino acids (no methionine) and divided in half; one sample was supplemented with 1mM methionine (Barstar-MET) while the other was supplemented with 1mM AHA (Barstar-AHA). After 15 min at 37 °C with

shaking, protein expression was induced with 1 mM IPTG. Cultures were clarified by centrifugation 3 h post induction and the Barstar proteins were purified under denaturing conditions using Ni-NTA spin columns according to the manufacturer's specifications (Qiagen). The purified proteins were verified by ESI-HRMS, calcd for Barstar-MET ($C_{521}H_{808}N_{146}O_{156}S_2$): 11677.1 Da (avg. mass), found 11676.8 Da; calcd for Barstar-AHA ($C_{519}H_{802}N_{152}O_{156}$): 11667.1 Da (avg. mass), found 11667.4 Da

Conjugation Reactions

10 μg of Barstar-MET or Barstar-AHA were acetone precipitated and dissolved in 2.5 μL of PBS. 2.5 μL of 1 mM TMTH in 1% DMSO in PBS were then added to the samples and the samples allowed to incubate at room temperature for 5 d. The proteins were then acetone precipitated and submitted to LC-MS analysis.

LC-MS Analysis

Samples were subjected to RP chromatography with an Agilent 1200 LC system that was connected in-line with an LTQ Orbitrap XL hybrid mass spectrometer. External mass calibration was performed prior to analysis. A binary solvent system consisting of buffer A (0.1% formic acid in water (v/v)) and buffer B (0.1% formic acid in acetonitrile (v/v)) was employed.

For the intact Barstar samples, the mass spectrometer was outfitted with an Ion Max electrospray ionization source. The LC was equipped with a Poroshell 300SB-C8 column and a 100 mL sample loop. For each run, after 100 to 200 picomoles of protein was injected onto the column, analyte trapping was performed for 5 min with 0.5% B, followed by a linear gradient from 30% to 95% B over 19.5 min, and finished with a washing step in 95% B for 5 min. The flow rate was maintained at 90 mL min^{-1}. Mass spectra were recorded in positive ion mode over the *m/z* scan range of 500 to 2000 using the Orbitrap mass analyzer in full-scan, profile mode. Raw mass spectra were processed using Xcalibur (version 4.1, Thermo) and measured charge state distributions were deconvoluted using ProMass (version 2.5 SR-1, Novatia), using the default "small protein" parameters and a background subtraction factor of 1.5.

Scheme S4

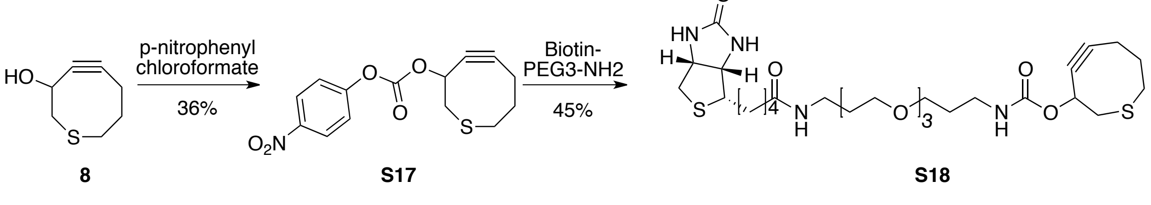

2-thiocyclooct-7-yn-1-(*p*-nitrophenyl)carbonate (S17).

ThiaOCT **8** (6.0 mg, 0.042 mmol, 1.0 equiv) was dissolved in dichloromethane (1.6 mL, anhydrous). To this solution, pyridine (21 μL, 0.26 mmol, 6.2 equiv, anhydrous) and *p*-nitrophenylchloroformate (20 mg, 0.10 mmol, 2.4 equiv) were added. The reaction mixture was stirred overnight and the resulting mixture was evaporated to dryness. The crude product was purified by silica gel chromatography (10:1 to 6:1 hexane/EtOAc) to

give desired product **S17** (4.5 mg, 0.015 mmol, 36%). R_f = 0.55 in 3:1 hexane/EtOAc. ^{1}H NMR (500 MHz, $CDCl_3$): δ 8.26 (d, *J* = 9.1 Hz, 2H), 7.37 (d, *J* = 9.5 Hz, 2H), 5.14-5.09 (m, 1H), 3.21 (d, *J* = 15.6 Hz, 1H), 3.07 (dt, *J* = 14.4, 3.7 Hz, 1H), 2.91 (dd, *J* = 15.2, 9.0 Hz, 1H), 2.81-2.69 (m, 2H), 2.54-2.37 (m, 3H). ^{13}C NMR (150 MHz, $CDCl_3$) δ 155.3, 151.4, 145.2, 125.3, 121.7, 92.3, 88.0, 82.9, 40,4, 38.5, 25.4, 23.2. HRMS (EI): calcd for $C_{14}H_{13}O_5NS^+$ [M]$^+$, 307.0514; found, 307.0514; [M-NO_2]$^+$, 261; [M-$C_7H_5NO_5$]$^+$, 124.

2-thiocyclooct-7-yn-1-(Biotin-PEG$_3$)carbamate (S18).

2-thiocyclooct-7-yn-1-(*p*-nitrophenyl)carbonate **S17** (4.5 mg, 0.015 mmol, 1.0 equiv) was dissolved in DMF (380 μL, anhdyrous). To this solution, triethylamine (5 μL, 0.036 mmol, 2.4 equiv) and Biotin-PEG$_3$-amine[6] (7.4 mg, 0.0156 mmol, 1.0 equiv) were added. The reaction was stirred overnight at rt. The following day the reaction mixture was evaporated to dryness and the crude product was purified by silica gel chromatography (10:1 to 4:1 hexanes/EtOAc). This procedure resulted in 4.2 mg of desired product (0.0068 mmol, 45 %). R_f = 0.2 in 3:1 hexane/EtOAc. ^{1}H NMR (600 MHz, MeOD): δ 4.94-4.90 (m, 1H), 4.50-4.48 (m, 1H), 4.31-4.29 (m, 1H), 3.64-3.61 (m, 4H), 3.60-3.57 (m, 4H), 3.53-3.50 (m, 4H), 3.26 (t, *J* = 6.8 Hz, 2H), 3.22-3.16 (m, 3H), 3.04 (d, *J* = 14.9 Hz, 2H), 2.93 (dd, *J* = 12.8, 4.9 Hz, 1H), 2.77 (dd, *J* = 15.1, 9.0 Hz, 1H), 2.70 (d, *J* = 12.7 Hz, 2H), 2.59-2.56 (m, 1H), 2.40-2.37 (m, 1H), 2.32-2.21 (m, 2H), 2.20 (t, *J* = 7.4 Hz, 2H), 1.77-1.68 (m, 8H), 1.43-1.46 (m, 2H). ^{13}C NMR (150 MHz, MeOD) δ 174.5, 164.7, 155.6, 88.7, 78.7, 70.1, 69.8, 69.8, 68.5, 68.3, 61.9, 60.2, 55.6, 40.7, 39.6, 38.0, 37.7, 36.4, 35.4, 29.4, 29.0, 28.4, 28.1, 25.4, 24.8, 22.5, 20.1. HRMS (ESI): calcd for $C_{28}H_{47}O_7N_4S_2^+$ [M+H]$^+$, 615.2881; found, 615.2874.

Cell culture procedures

Jurkat (human T-cell lymphoma) cells were grown in RPMI-1640 media containing 10% fetal bovine serum, streptomycin (0.1 mg/ mL), and penicillin (100 units/ mL). Cells were grown in the presence of 5% CO_2 and maintained at densities between 1 x 10^5 and 2 x 10^6 cells/ mL.

Cell surface azide labeling

Jurkat cells were incubated in the described media containing 25 μM Ac$_4$ManNAz or DMSO vehicle for 3 days. The cells were then pelleted (1500 rpm, 3 min, 4 ºC) and resuspended in 10 mL FACS buffer (PBS with 1% fetal bovine serum) twice. The cell suspension was then transferred to a 96 well V-bottom plate. The cells were pelleted by centrifugation (3500 rpm, 3 min, 4 ºC). The cells were then resuspended PBS containing 250 μM thiaOCT-biotin, DIMAC-biotin, or vehicle and incubated at rt for 1 h. The cells were washed with FACS buffer (3 x 200 μL, 3500 rpm, 3 min, 4 ºC) and resuspended in FACS buffer containing FITC-avidin (100 μL, 1:200 dilution of 1 mg/ mL stock, Sigma-Aldrich) and incubated in the dark at 4 ºC for 15 minutes. The cells were washed with FACS buffer (1 x 200 μL, 3500 rpm, 3 min, 4 ºC) and again subjected to FITC-avidin (same conditions as first incubation). The cells were concentrated by centrifugation (3500 rpm, 3 min, 4 ºC) and resuspended in 200 μL cold FACS buffer three times. The cell

suspensions were then diluted to 400 μL for flow cytometry analysis. Flow cytometry was performed on a BD Biosciences FACSCalibur flow cytometer equipped with a 488-nm argon laser.

Figure S10

A

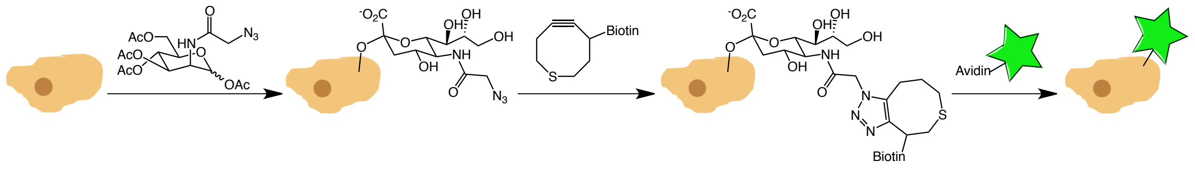

B

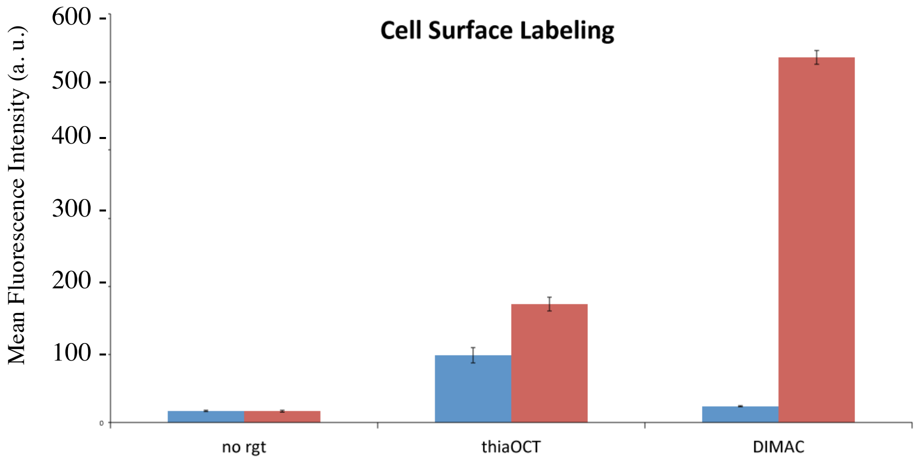

Figure S10.
ThiaOCT can label azides on cell surfaces. A) Schematic for flow cytometry analysis of live cell labeling with thiaOCT. B) Quantificaton of cell-surface labeling with thiaOCT-biotin (**S18**). Cells are grown in the presence (red bars) or absence (blue bars) of 25 μM $Ac_4ManNAz$ for 3 days. The cells were then treated with no reagent, 250 μM thiaOCT-biotin (**S18**), or 250 μM DIMAC-biotin[7] followed by FITC-avidin. Error bars represent the standard deviation of three replicate experiments.

Figure S11

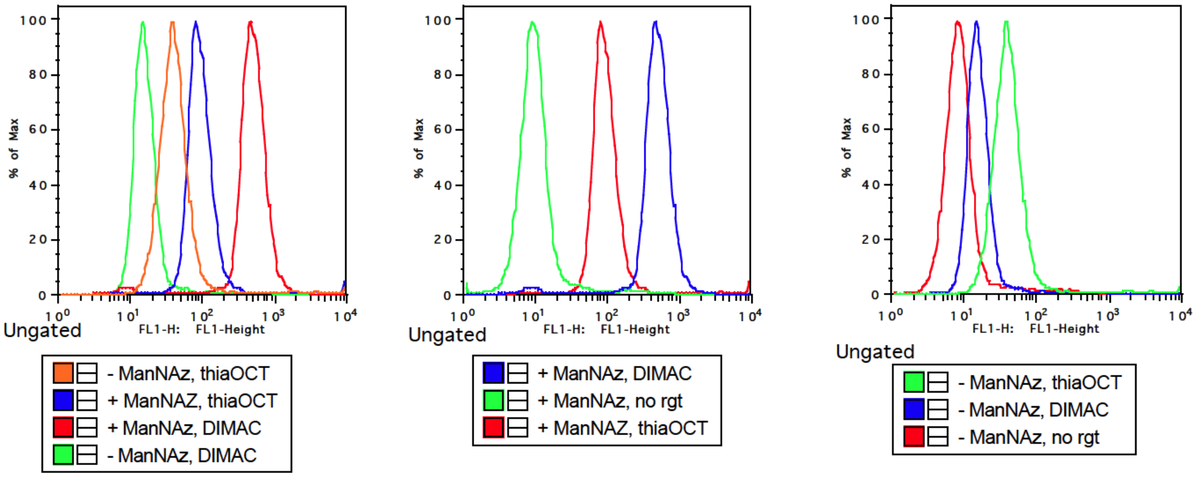

Figure S11.
Representative histograms of fluorescence (x-axis) vs % of total cell counts (y-axis) for the experiment described in Figure S10. Jurkat cells were treated with (+ ManNAz) or without (- ManNAz) 25 µM Ac_4ManNAz for 3 days and then treated with 250 µM thiaOCT-biotin, DIMAC-biotin, or vehicle followed by FITC-avidin.

Figure S12

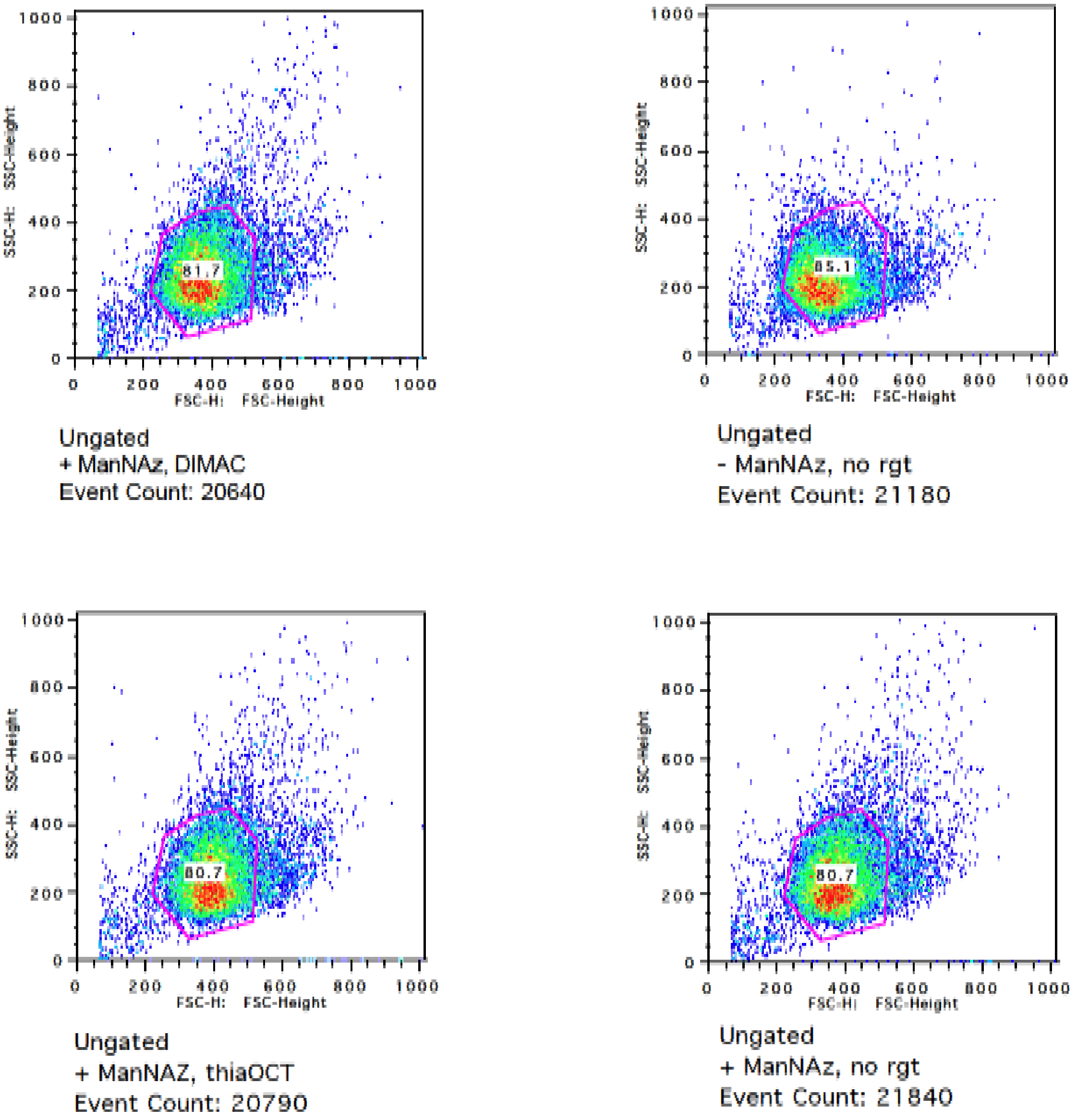

Figure S12.
Representative forward-scatter (x-axis) and side-scatter (y-axis) plots for the experiment described in Figure S10. Jurkat cells were treated with (+ ManNAz) or without (- ManNAz) 25 μM Ac_4ManNAz for 3 days and then treated with 250 μM thiaOCT-biotin, DIMAC-biotin,[6] or vehicle followed by FITC-avidin.

Figure S13

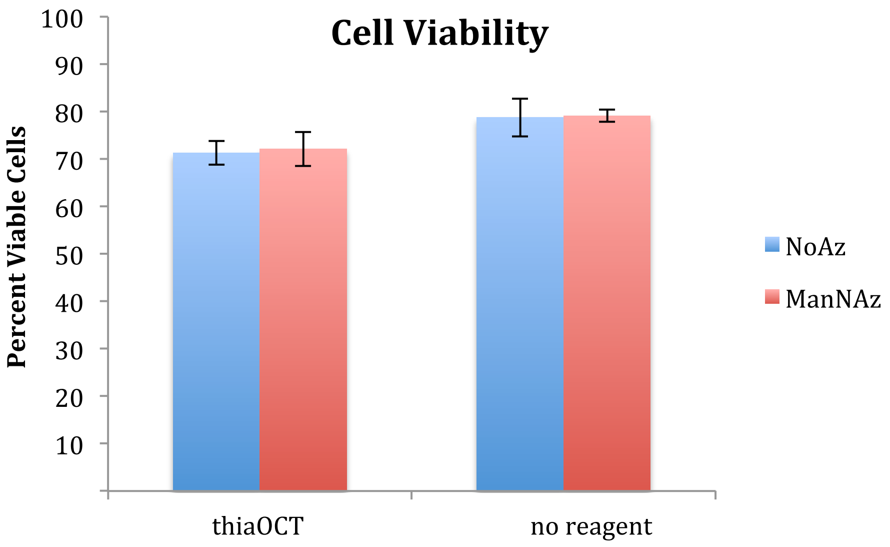

Figure S13.
Cytotoxicity analysis of thiaOCT-biotin **S18**. Jurkat cells were treated with (ManNAz, red bars) or without (NoAz, blue bars) 25 μM $Ac_4ManNAz$ for 3 days and then treated with 250 μM thiaOCT-biotin or vehicle followed by FITC-avidin. Prior to flow cytometry analysis, the cells were treated with 7-amino-actinomycin D (7-AAD) following the provided procedure.[8] The samples were diluted and analyzed by flow cytometry. The error bars represent standard deviations from three replicates.

Figure S14.

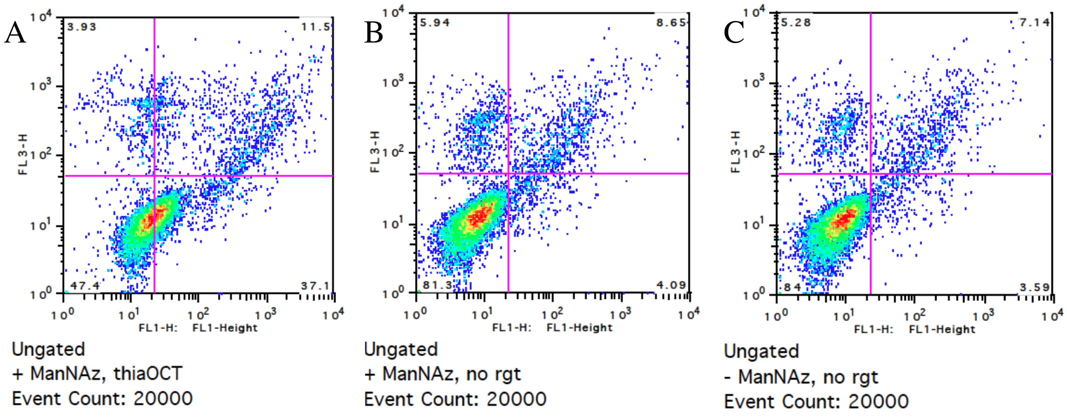

Figure S14.
Representative FL3 vs. FL1 scatter plots for the flow cytometry experiments described in Figure S13. In all plots, the x-axis indicates the degree of cell-surface glycan labeling as measured by FITC fluorescence (FL1), and the y-axis represents the degree of 7-AAD (FL3, cell viability marker) measured. Jurkat cells were treated with (A, B) or without (C) 25 μM $Ac_4ManNAz$ for 3 days and then treated with 250 μM thiaOCT-biotin (A) or

vehicle (B, C) followed by FITC-avidin. Prior to flow cytometry analysis, the cells were treated with 7-AAD following the provided procedure.[8] The samples were diluted and analyzed by flow cytometry.

---

[1] A. B. Pangborn, M. A. Giardello, R. H. Grubbs, R. K. Rosen, F. J. Timmers, *Organometallics* **1996**, *15*, 1518.

[2] Okamoto, S.; Livinghouse, T. *J. Am. Chem. Soc.* **2000**, *122*, 1223-1224.

[3] N. Feeder, M. J. Ginnelly, R. V. H. Jones, S. O'Sullivan, S. Warren, *Tet. Lett.* **1994**, *48*, 9095.

[4] Kimling, H., Krebs, A., *Liebigs Ann. Chem.* **1974**, 2074.

[5] K. E. Beatty, F. Xie, Q. Wang, D. A. Tirrell, *J. Am. Chem. Soc.* **2005**, *127*

[6] D. S. Wilbur, D. K. Hamlin, R. L. Vessella, J. E. Stray, K. R. Buhler, P. S. Strayton, L. A. Klumb, P. M. Pathare, S. A. Weerawarna, *Bioconjug. Chem.* **1996**, *7*, 689.

[7] E. M. Sletten, C. R. Bertozzi, *Org. Lett.* **2008**, *10*, 3097.

[8] http://www.bdbiosciences.com/external_files/pm/doc/tds/cell_bio/live/web_enabled/68981E_559925.pdf

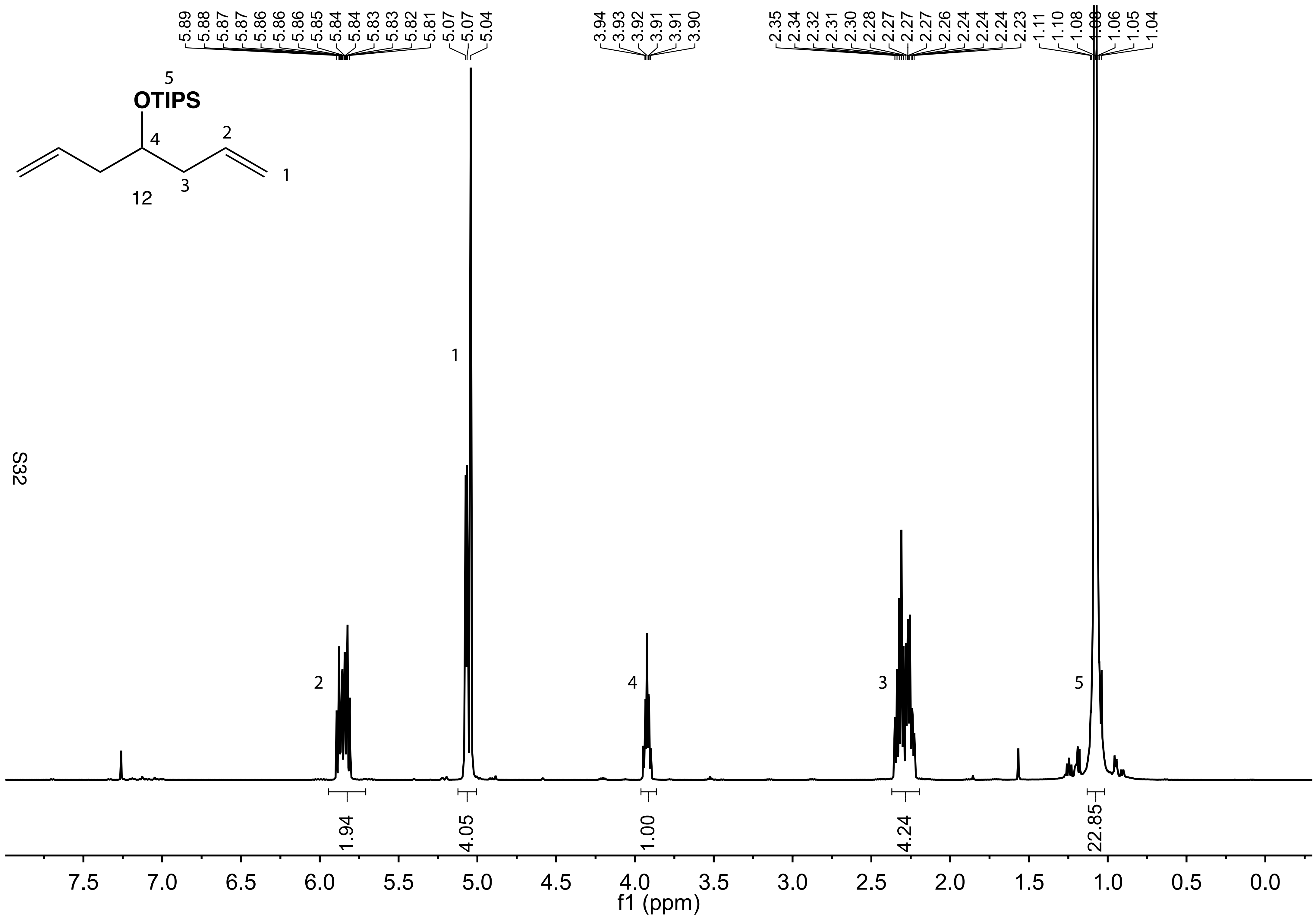
5.89 5.88 5.87 5.87 5.86 5.86 5.86 5.85 5.84 5.84 5.83 5.83 5.82 5.81
5.07 5.07 5.04
3.94 3.93 3.92 3.91 3.91 3.90
2.35 2.34 2.32 2.31 2.30 2.28 2.27 2.27 2.27 2.26 2.24 2.24 2.24 2.23
1.11 1.10 1.08 1.08 1.06 1.05 1.04
OTIPS
5
4
3
2
1
12
1.94
4.05
1.00
4.24
22.85
7.5 7.0 6.5 6.0 5.5 5.0 4.5 4.0 3.5 3.0 2.5 2.0 1.5 1.0 0.5 0.0
f1 (ppm)

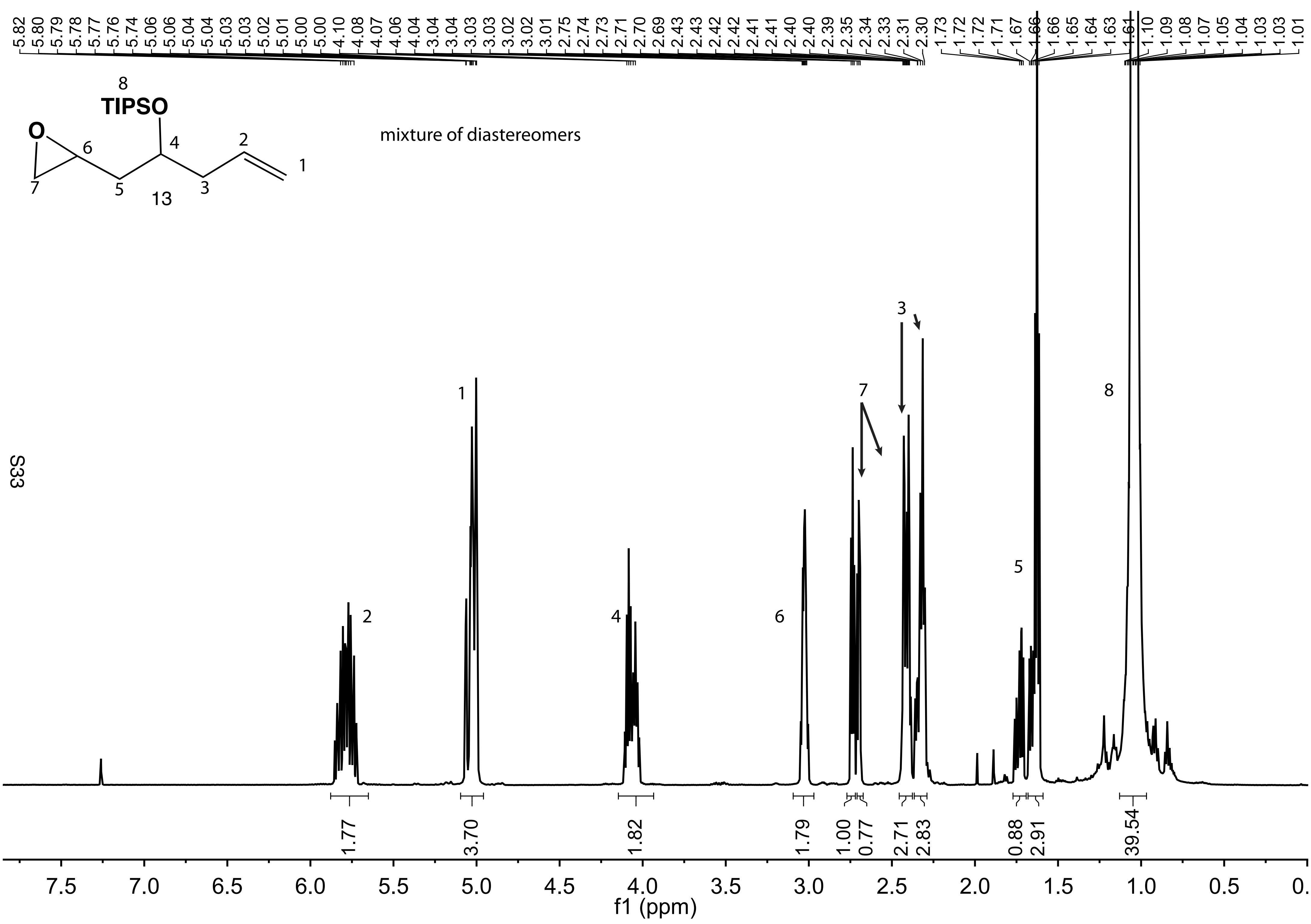

TIPSO
O
13
mixture of diastereomers
f1 (ppm)
1.77
3.70
1.82
1.79
1.00
0.77
2.71
2.83
0.88
2.91
39.54

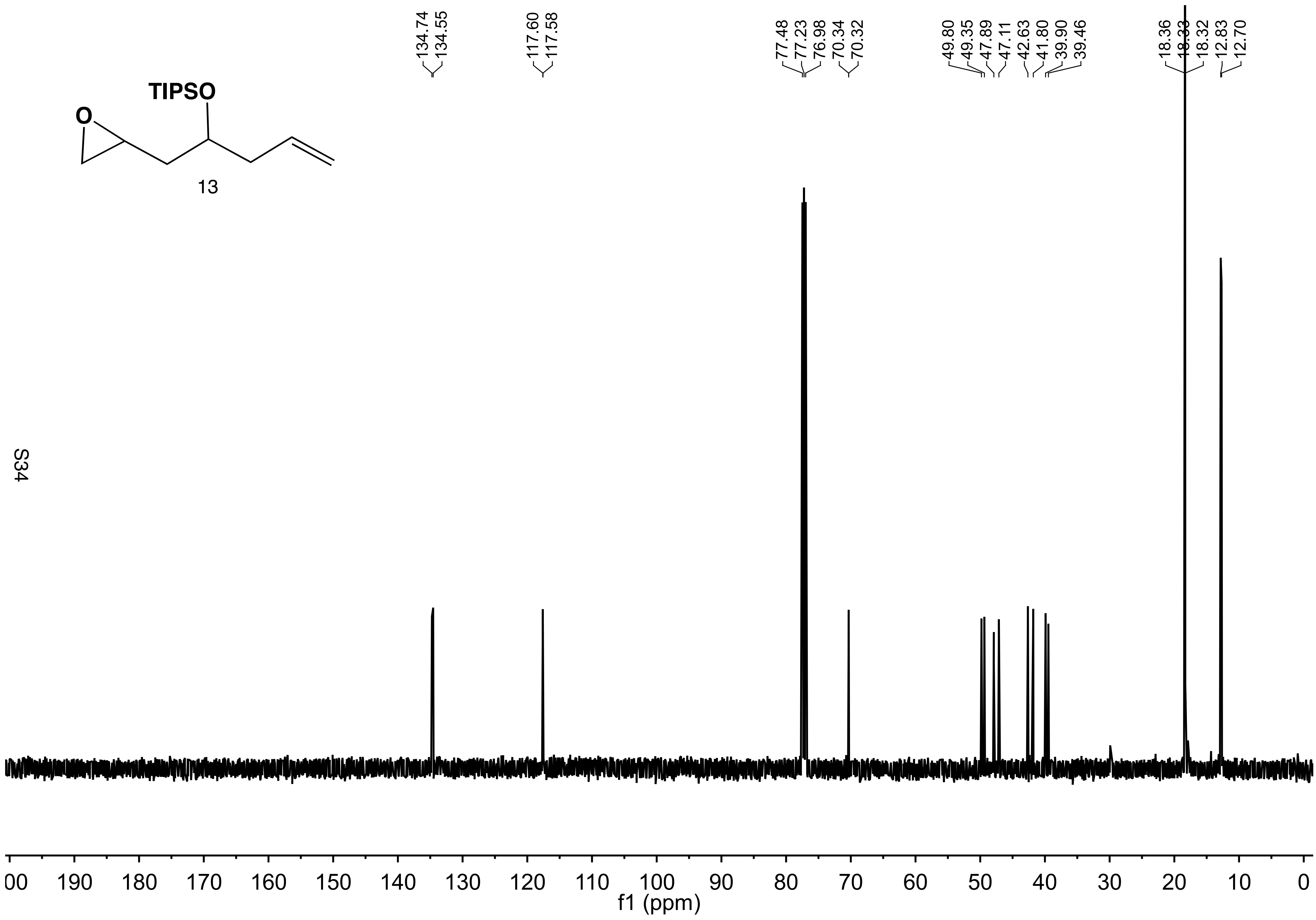

TIPSO
O
134.74
134.55
117.60
117.58
77.48
77.23
76.98
70.34
70.32
49.80
49.35
47.89
47.11
42.63
41.80
39.90
39.46
18.36
18.33
18.32
12.83
12.70
200 190 180 170 160 150 140 130 120 110 100 90 80 70 60 50 40 30 20 10 0
f1 (ppm)

13

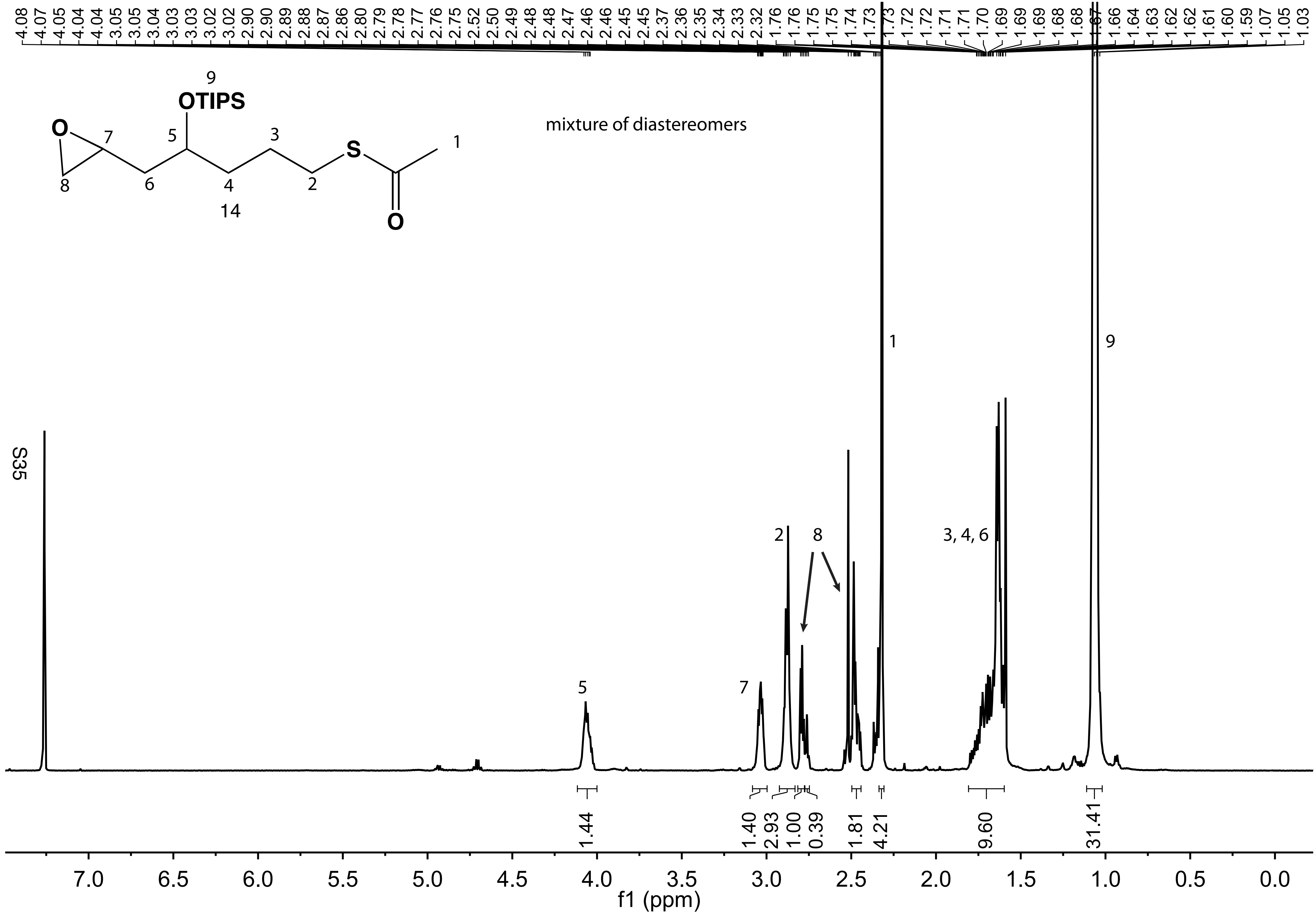
4.08 4.07 4.05 4.04 4.04 3.05 3.05 3.04 3.03 3.03 3.02 3.02 2.90 2.90 2.89 2.88 2.87 2.86 2.80 2.79 2.78 2.77 2.76 2.75 2.52 2.50 2.49 2.48 2.48 2.47 2.46 2.46 2.45 2.45 2.37 2.36 2.35 2.34 2.33 2.32 1.76 1.76 1.75 1.75 1.74 1.73 1.73 1.72 1.72 1.71 1.71 1.70 1.69 1.69 1.69 1.69 1.68 1.68 1.67 1.66 1.64 1.63 1.62 1.62 1.61 1.60 1.59 1.07 1.05 1.03
OTIPS
S
O
mixture of diastereomers
14
1
9
2
8
3, 4, 6
5
7
1.44
1.40
2.93
1.00
0.39
1.81
4.21
9.60
31.41
f1 (ppm)

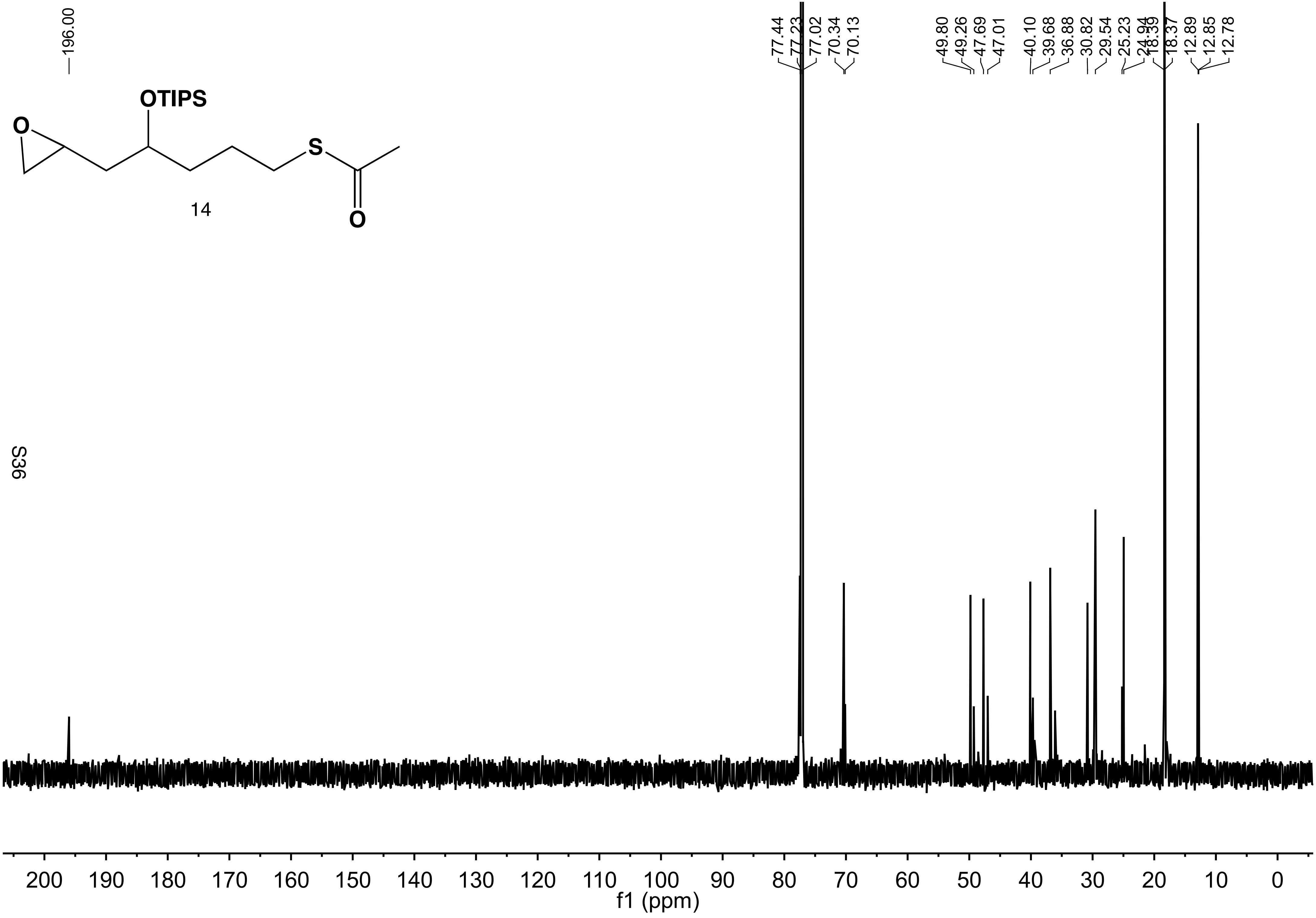

196.00
77.44
77.23
77.02
70.34
70.13
49.80
49.26
47.69
47.01
40.10
39.68
36.88
30.82
29.54
25.23
24.94
18.39
18.37
12.89
12.85
12.78
OTIPS
O
S
O
14
200
190
180
170
160
150
140
130
120
110
100
90
80
70
60
50
40
30
20
10
0
f1 (ppm)

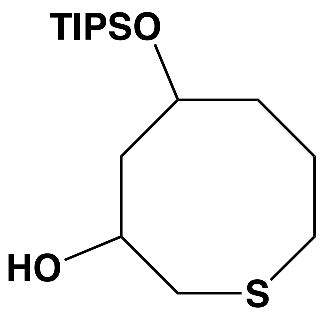

15

diastereomers

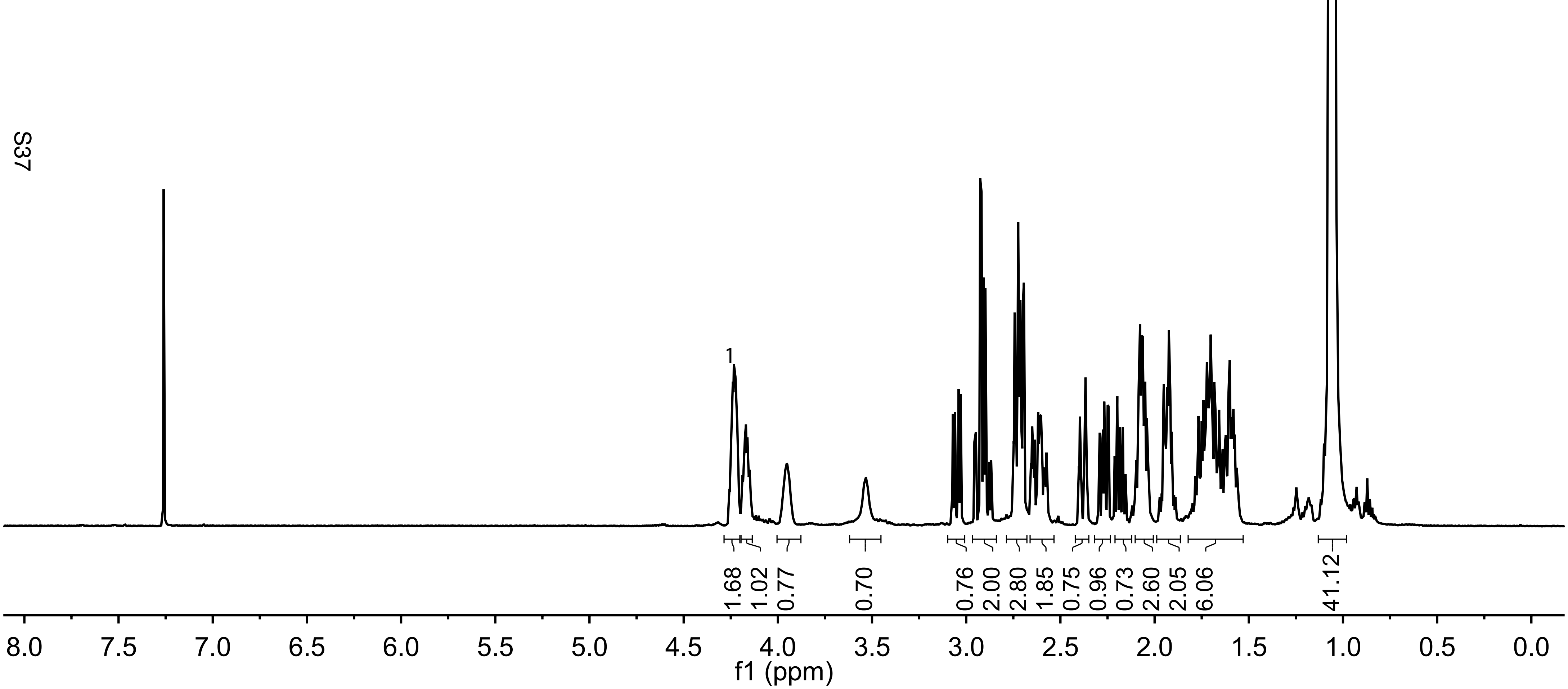

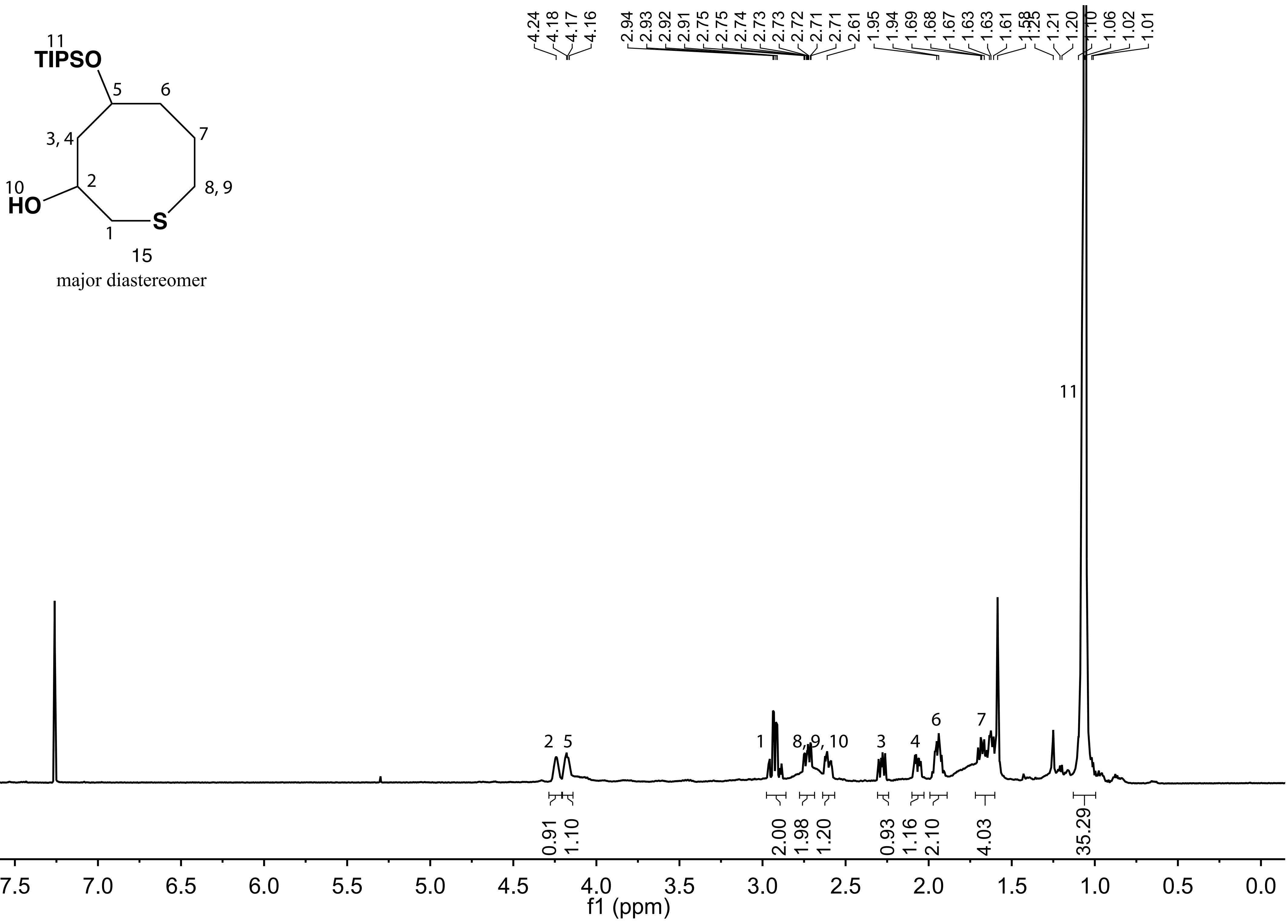
11
TIPSO
5
6
3, 4
7
10
HO
2
8, 9
1
S
15
major diastereomer
4.24
4.18
4.17
4.16
2.94
2.93
2.92
2.91
2.75
2.75
2.74
2.73
2.73
2.72
2.71
2.71
2.61
1.95
1.94
1.69
1.68
1.67
1.63
1.63
1.61
1.58
1.25
1.21
1.20
1.10
1.06
1.02
1.01
11
2 5
1
8, 9, 10
3
4
6
7
0.91
1.10
2.00
1.98
1.20
0.93
1.16
2.10
4.03
35.29
7.5
7.0
6.5
6.0
5.5
5.0
4.5
4.0
3.5
3.0
2.5
2.0
1.5
1.0
0.5
0.0
f1 (ppm)

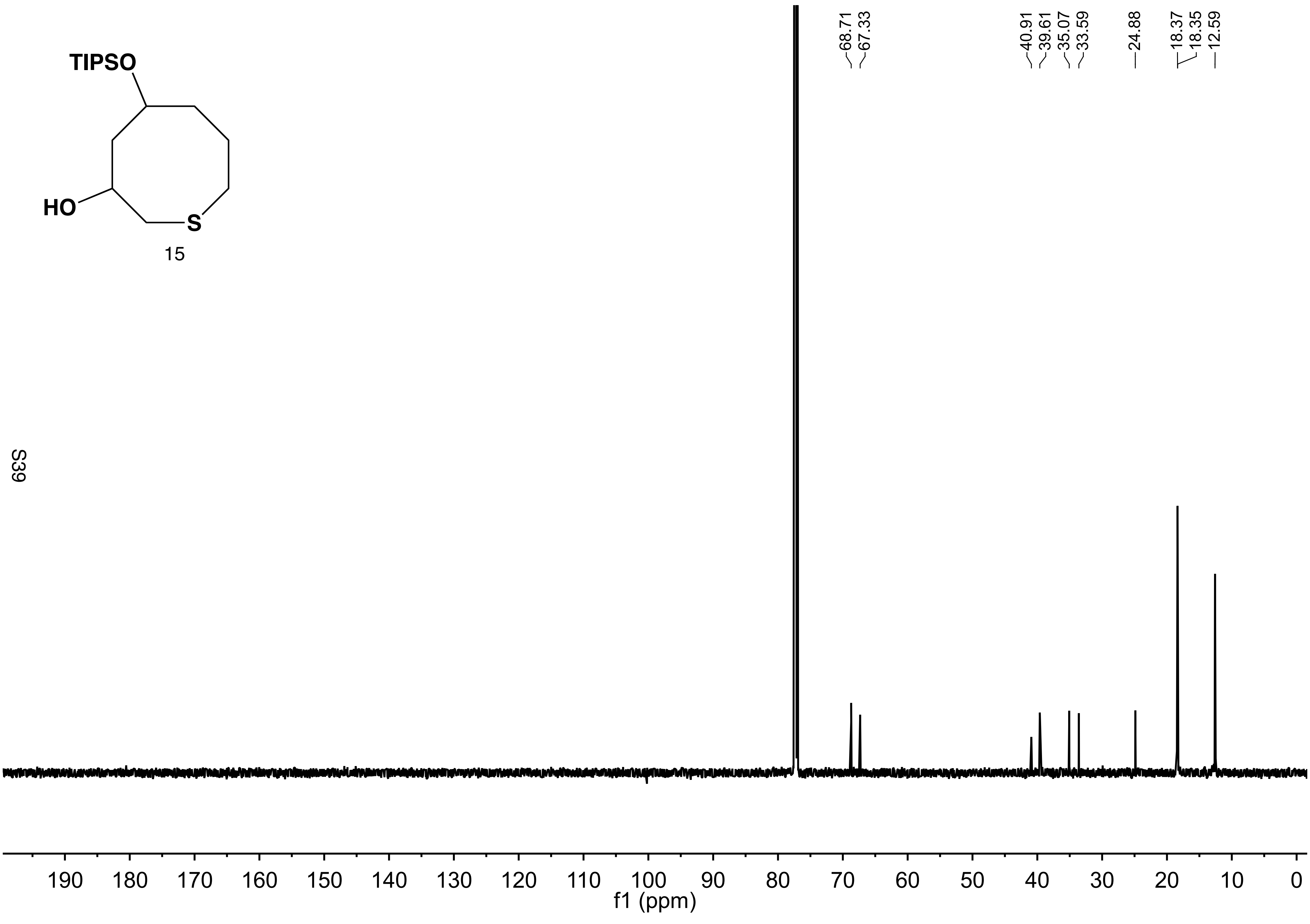

TIPSO
HO
S
15
68.71
67.33
40.91
39.61
35.07
33.59
24.88
18.37
18.35
12.59
190 180 170 160 150 140 130 120 110 100 90 80 70 60 50 40 30 20 10 0
f1 (ppm)

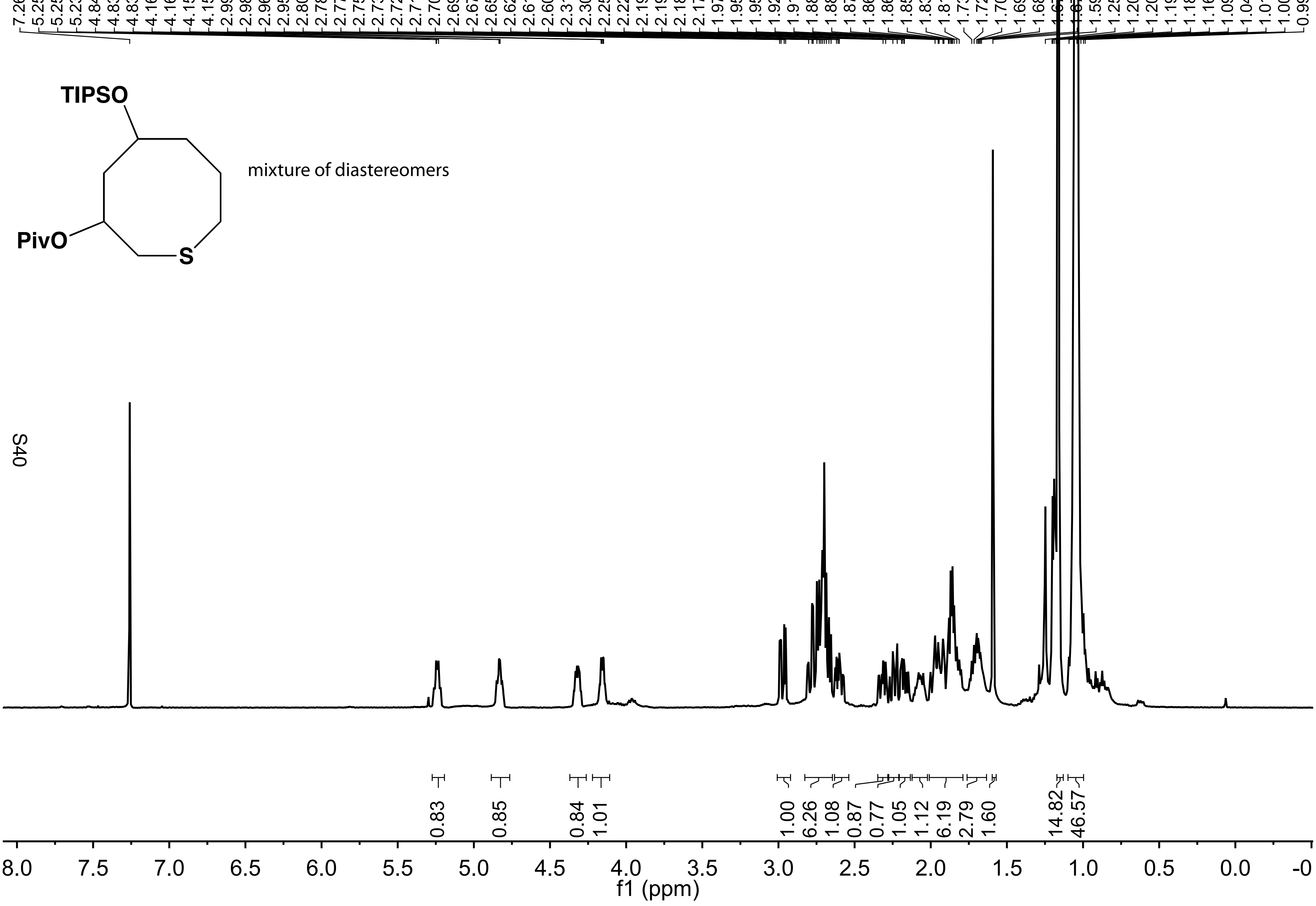
7.26
5.25
5.25
5.23
4.84
4.83
4.16
4.16
4.15
4.15
2.99
2.98
2.96
2.95
2.80
2.78
2.77
2.75
2.73
2.72
2.71
2.70
2.69
2.67
2.65
2.62
2.61
2.60
2.31
2.30
2.25
2.22
2.19
2.19
2.18
2.17
1.97
1.95
1.95
1.92
1.91
1.88
1.88
1.87
1.86
1.86
1.85
1.83
1.81
1.73
1.72
1.70
1.69
1.68
1.67
1.67
1.59
1.25
1.20
1.20
1.19
1.18
1.16
1.09
1.04
1.01
1.00
0.99
TIPSO
PivO
S
mixture of diastereomers
0.83
0.85
0.84
1.01
1.00
6.26
1.08
0.87
0.77
1.05
1.12
6.19
2.79
1.60
14.82
46.57
8.0
7.5
7.0
6.5
6.0
5.5
5.0
4.5
4.0
3.5
3.0
2.5
2.0
1.5
1.0
0.5
0.0
-0
f1 (ppm)

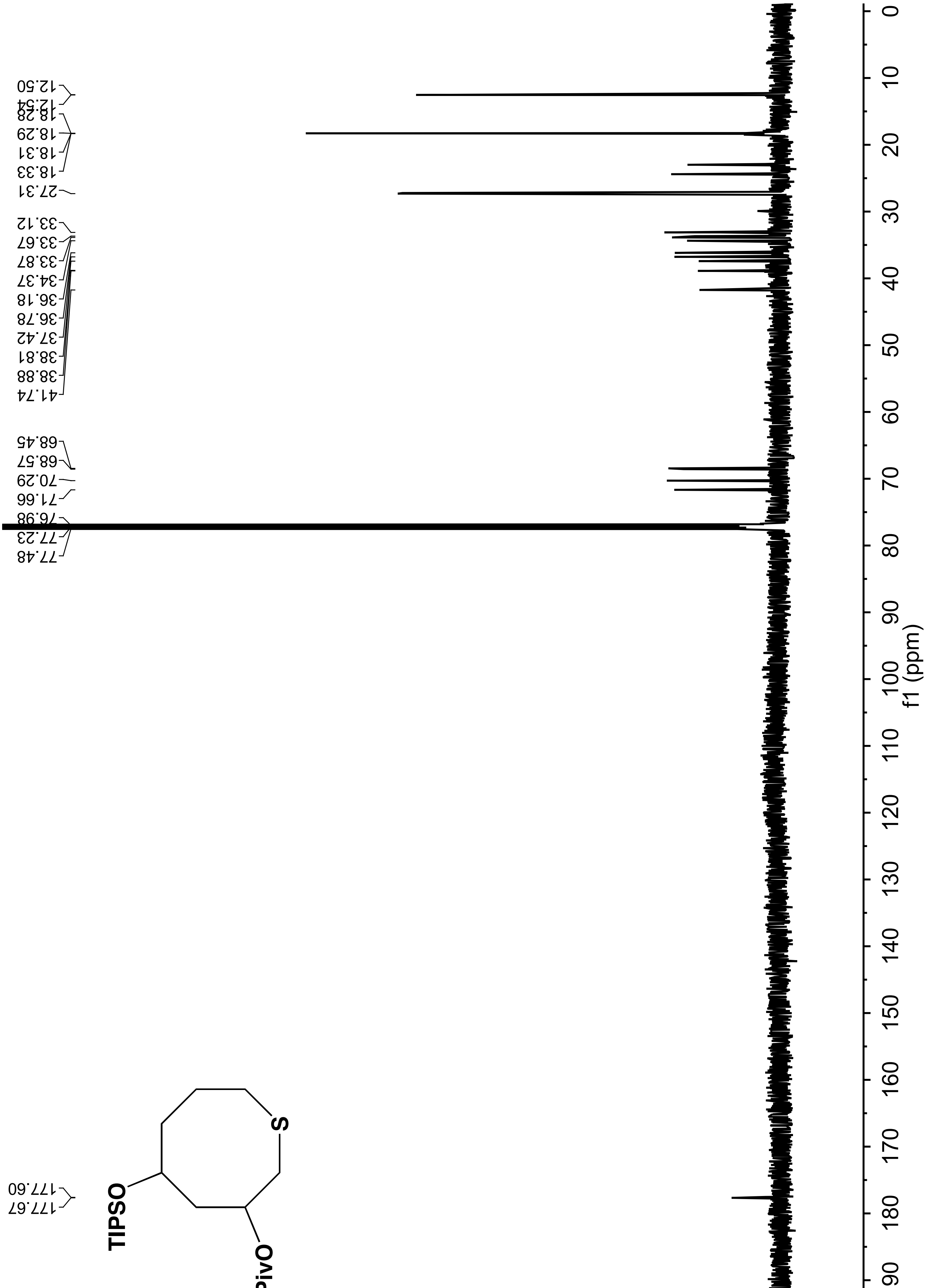
12.50
12.54
18.28
18.29
18.31
18.33
27.31
33.12
33.67
33.87
34.37
36.18
36.78
37.42
38.81
38.88
41.74
68.45
68.57
70.29
71.66
76.98
77.23
77.48
177.60
177.67
TIPSO
PivO
S
f1 (ppm)

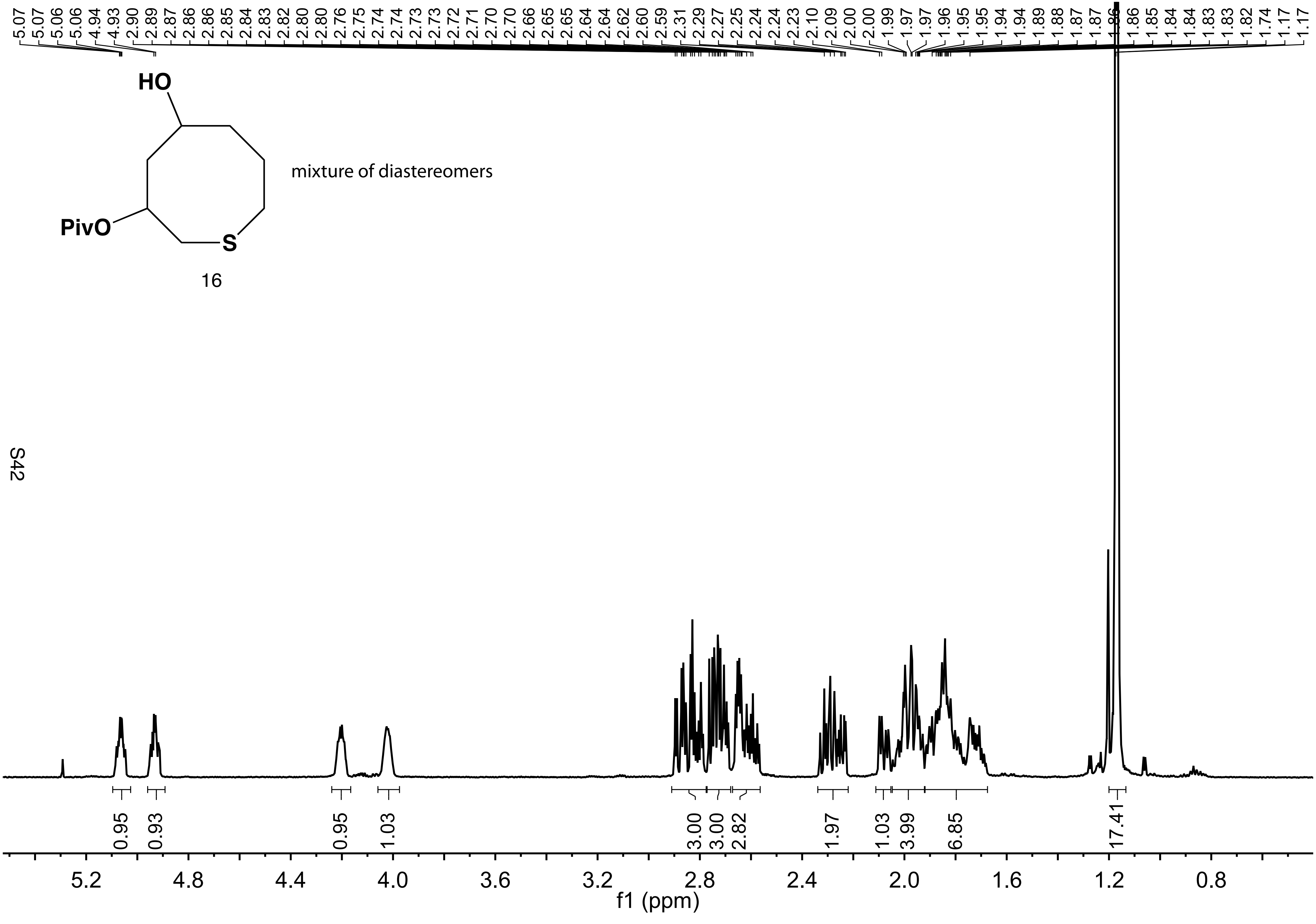
HO
PivO
S
16
mixture of diastereomers
5.07
5.07
5.06
5.06
4.94
4.93
2.90
2.89
2.87
2.86
2.86
2.85
2.84
2.83
2.82
2.80
2.80
2.76
2.75
2.74
2.74
2.73
2.73
2.72
2.71
2.70
2.70
2.66
2.65
2.65
2.64
2.64
2.62
2.60
2.59
2.31
2.29
2.27
2.25
2.24
2.24
2.23
2.10
2.09
2.00
2.00
1.99
1.97
1.97
1.96
1.96
1.95
1.95
1.94
1.94
1.89
1.88
1.87
1.87
1.86
1.86
1.85
1.84
1.84
1.83
1.83
1.82
1.74
1.17
1.17
0.95
0.93
0.95
1.03
3.00
3.00
2.82
1.97
1.03
3.99
6.85
17.41
5.2
4.8
4.4
4.0
3.6
3.2
2.8
2.4
2.0
1.6
1.2
0.8
f1 (ppm)

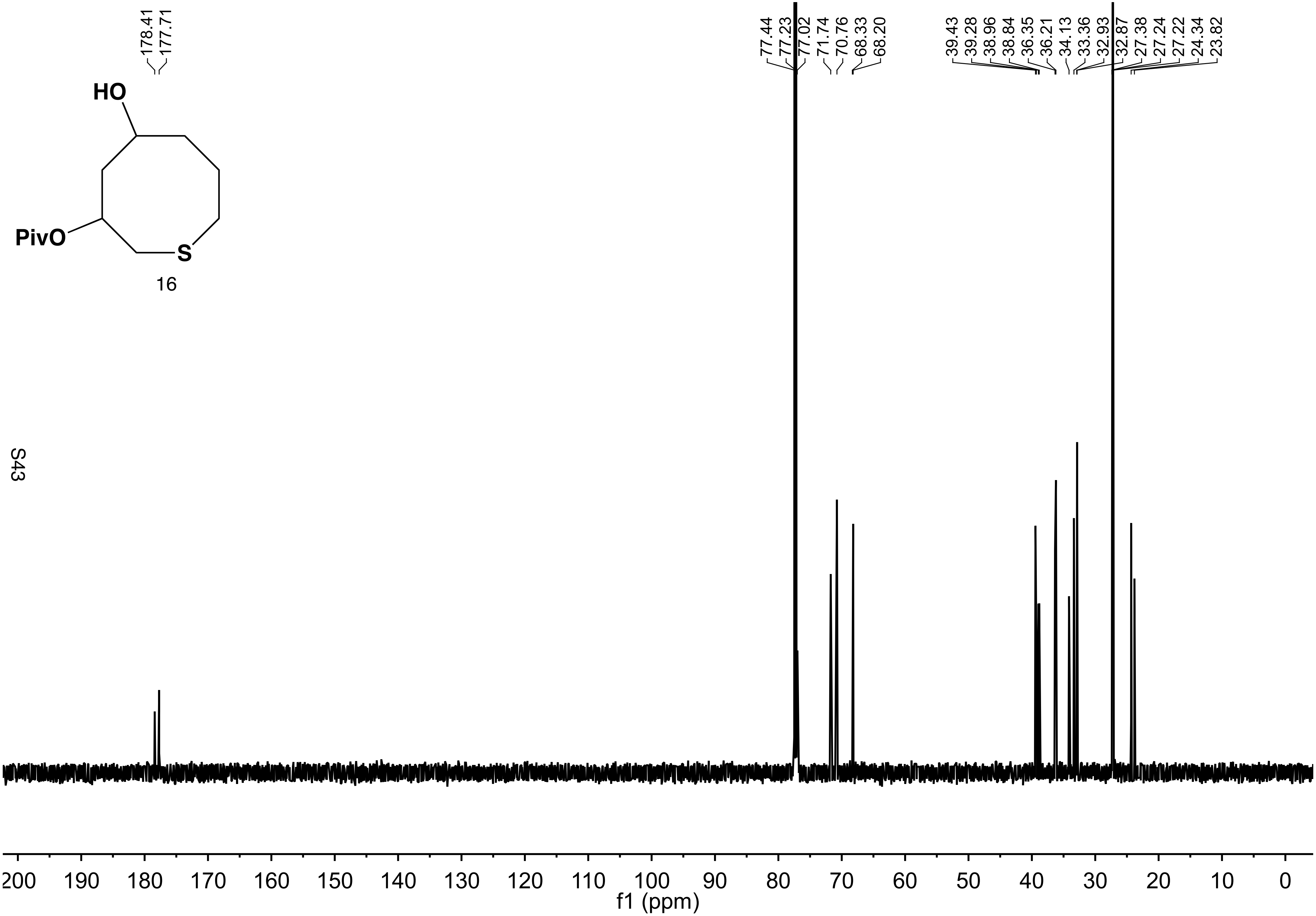

16

178.41
177.71
77.44
77.23
77.02
71.74
70.76
68.33
68.20
39.43
39.28
38.96
38.84
36.35
36.21
34.13
33.36
32.93
32.87
27.38
27.24
27.22
24.34
23.82
200
190
180
170
160
150
140
130
120
110
100
90
80
70
60
50
40
30
20
10
0
f1 (ppm)

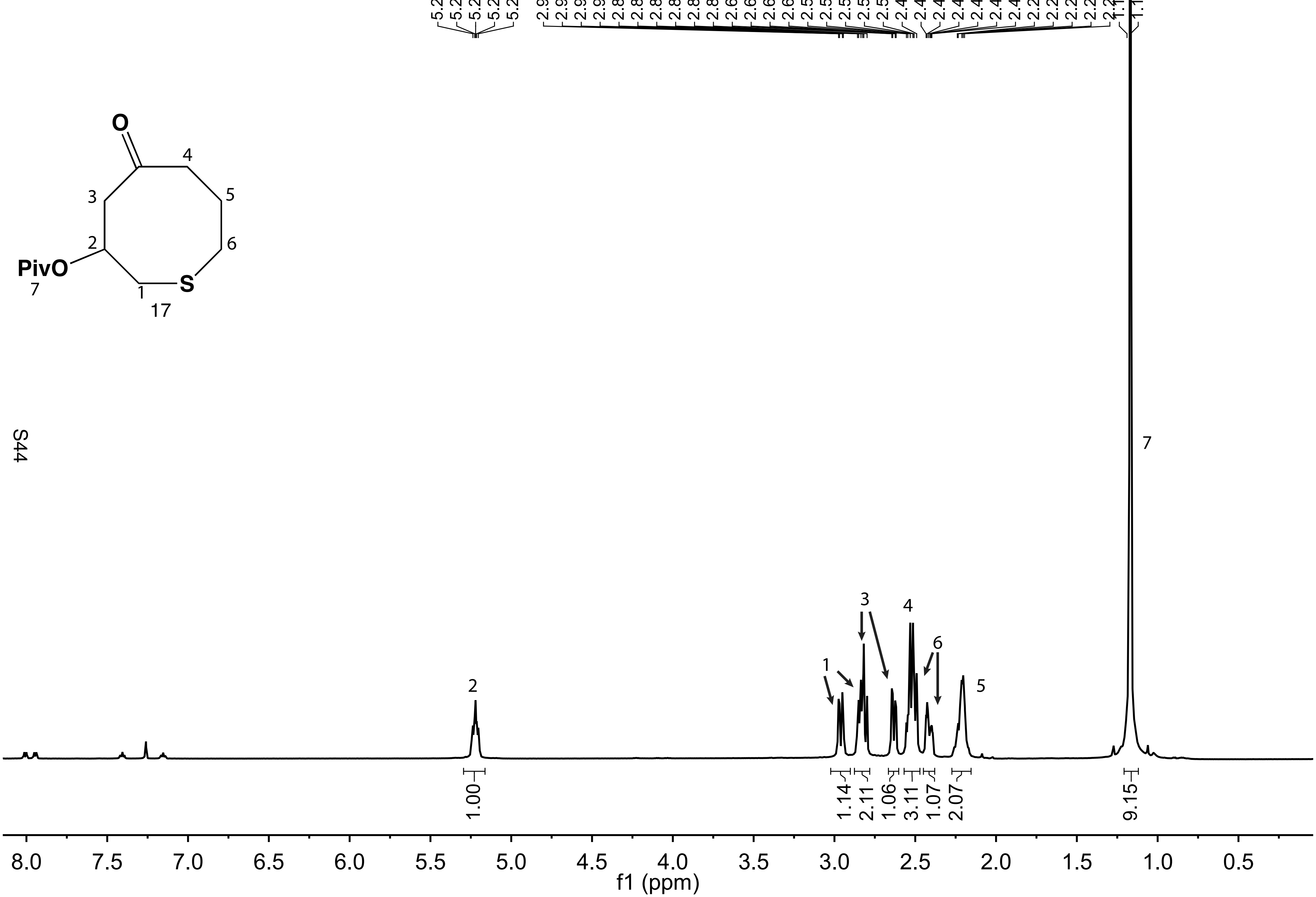
5.24 5.23 5.22 5.21 5.20
2.97 2.97 2.95 2.94 2.86 2.85 2.84 2.83 2.82 2.80 2.65 2.64 2.62 2.62 2.55 2.55 2.53 2.51 2.51 2.49 2.43 2.43 2.42 2.41 2.40 2.40 2.24 2.23 2.21 2.20 2.20 1.19 1.17
O
PivO
S
1
2
3
4
5
6
7
17
1.00
1.14
2.11
1.06
3.11
1.07
2.07
9.15
8.0 7.5 7.0 6.5 6.0 5.5 5.0 4.5 4.0 3.5 3.0 2.5 2.0 1.5 1.0 0.5
f1 (ppm)

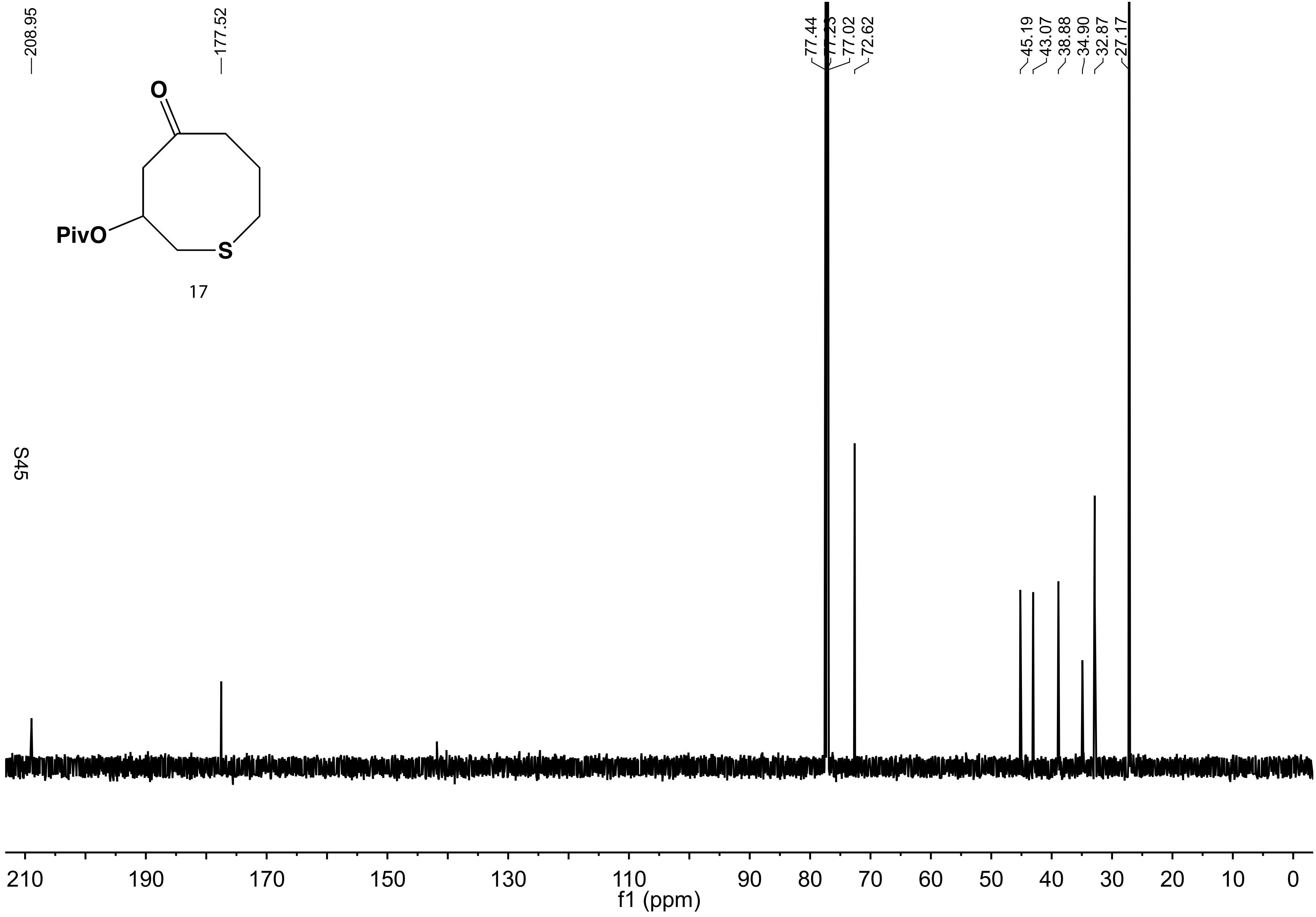
208.95
177.52
O
PivO
S
77.44
77.23
77.02
72.62
45.19
43.07
38.88
34.90
32.87
27.17
210
190
170
150
130
110
90
80
70
60
50
40
30
20
10
0
f1 (ppm)

17

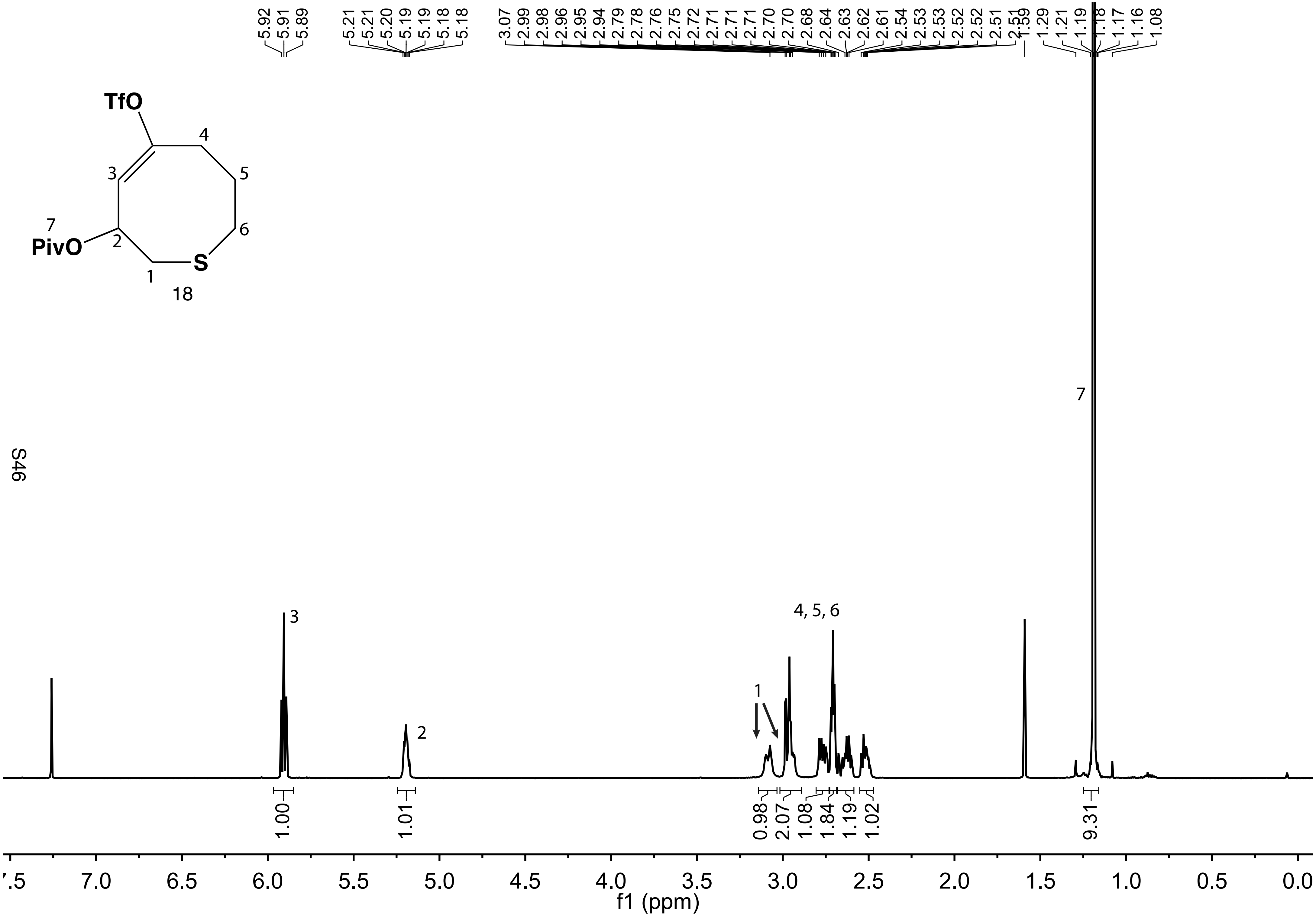
5.92
5.91
5.89
5.21
5.21
5.20
5.19
5.19
5.18
5.18
3.07
2.99
2.98
2.96
2.95
2.94
2.79
2.78
2.76
2.75
2.72
2.71
2.71
2.71
2.70
2.70
2.68
2.64
2.63
2.62
2.61
2.54
2.53
2.53
2.52
2.52
2.51
2.51
1.59
1.29
1.21
1.19
1.18
1.17
1.16
1.08
TfO
PivO
S
1
2
3
4
5
6
7
18
3
2
1
4, 5, 6
7
1.00
1.01
0.98
2.07
1.08
1.84
1.19
1.02
9.31
7.5
7.0
6.5
6.0
5.5
5.0
4.5
4.0
3.5
3.0
2.5
2.0
1.5
1.0
0.5
0.0
f1 (ppm)

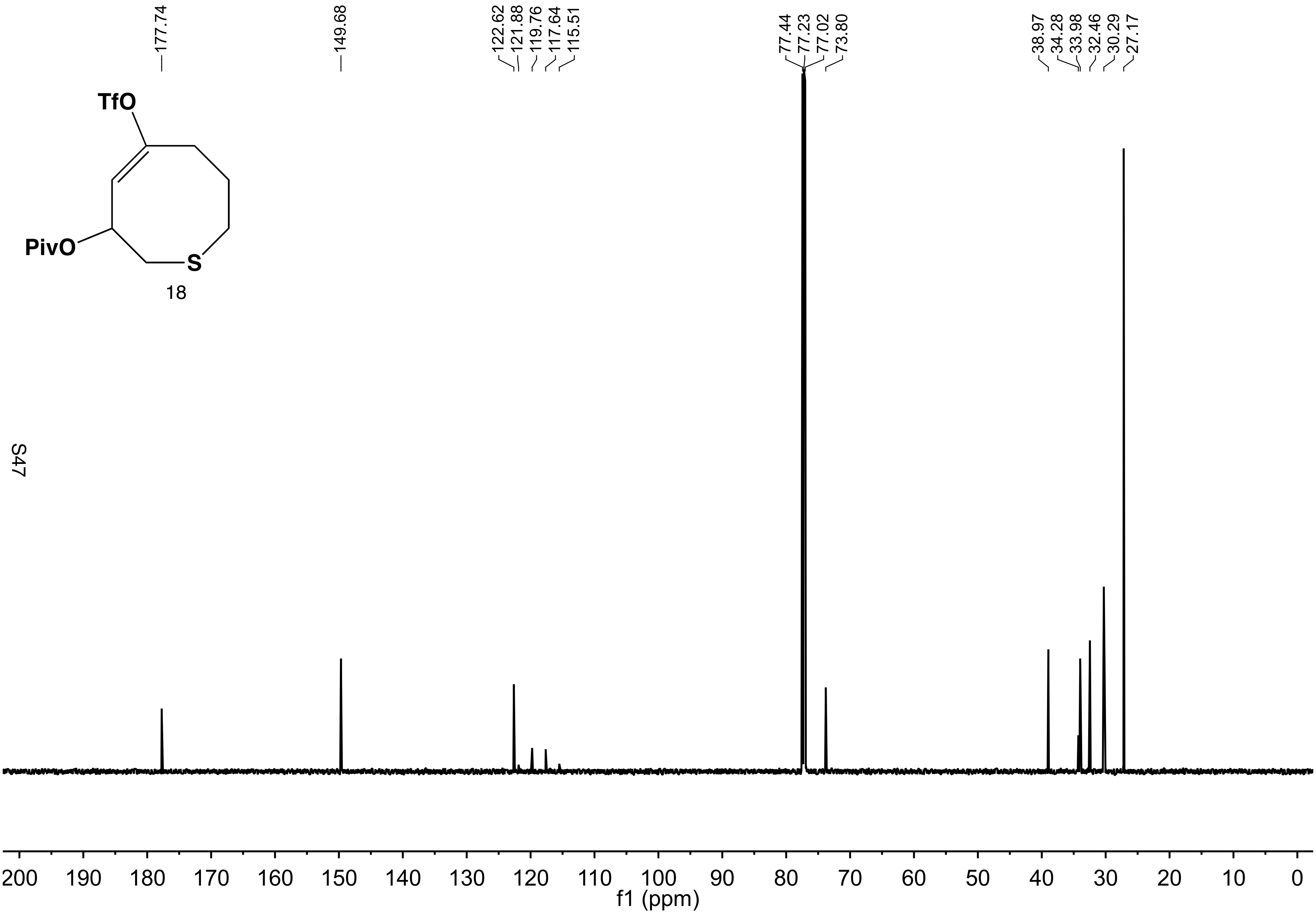
177.74
149.68
122.62
121.88
119.76
117.64
115.51
77.44
77.23
77.02
73.80
38.97
34.28
33.98
32.46
30.29
27.17
TfO
PivO
S
18
200
190
180
170
160
150
140
130
120
110
100
90
80
70
60
50
40
30
20
10
0
f1 (ppm)

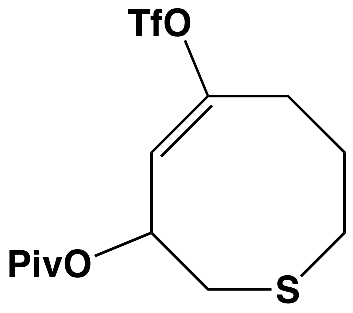
TfO
PivO
S

18

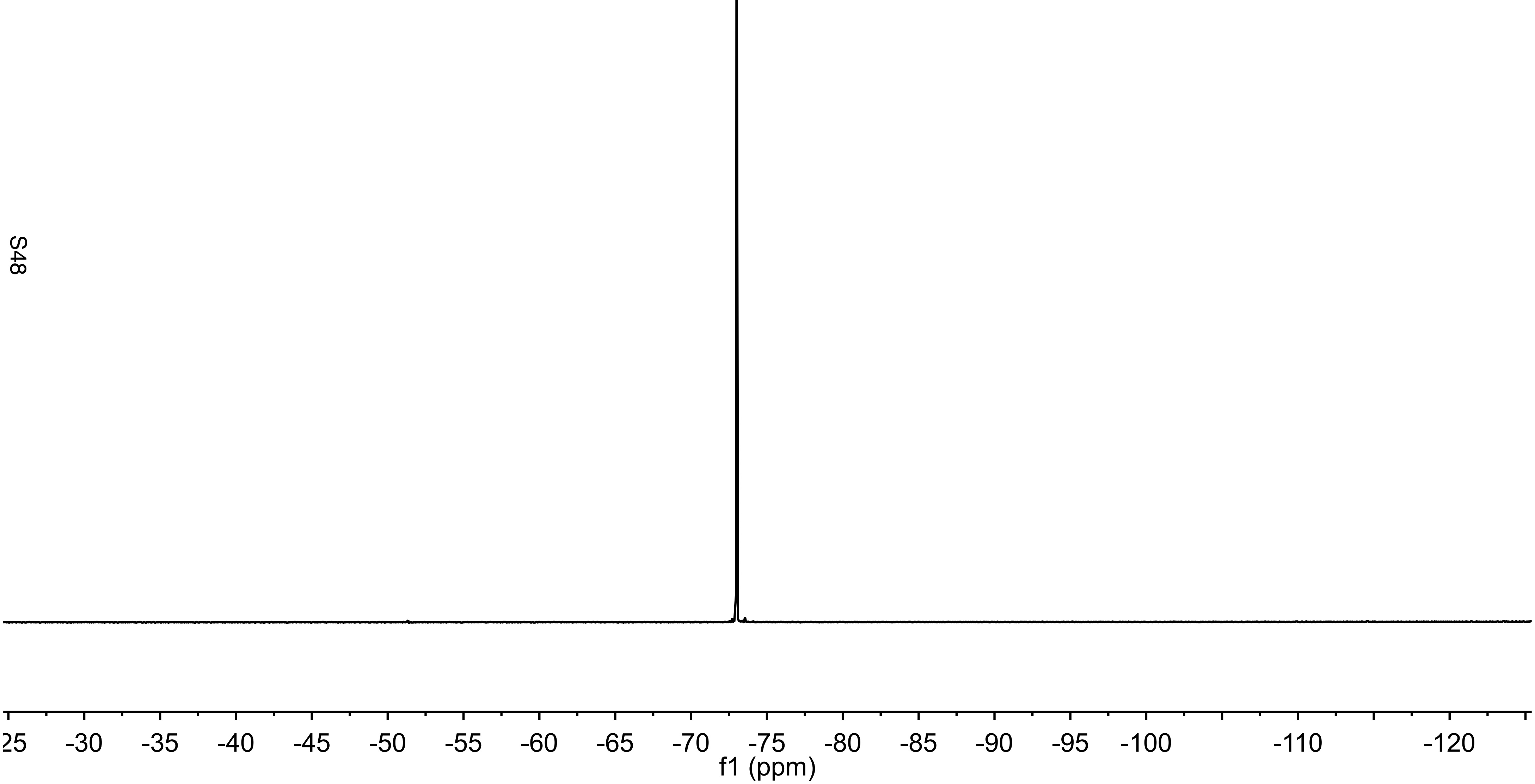
-25 -30 -35 -40 -45 -50 -55 -60 -65 -70 -75 -80 -85 -90 -95 -100 -110 -120
f1 (ppm)

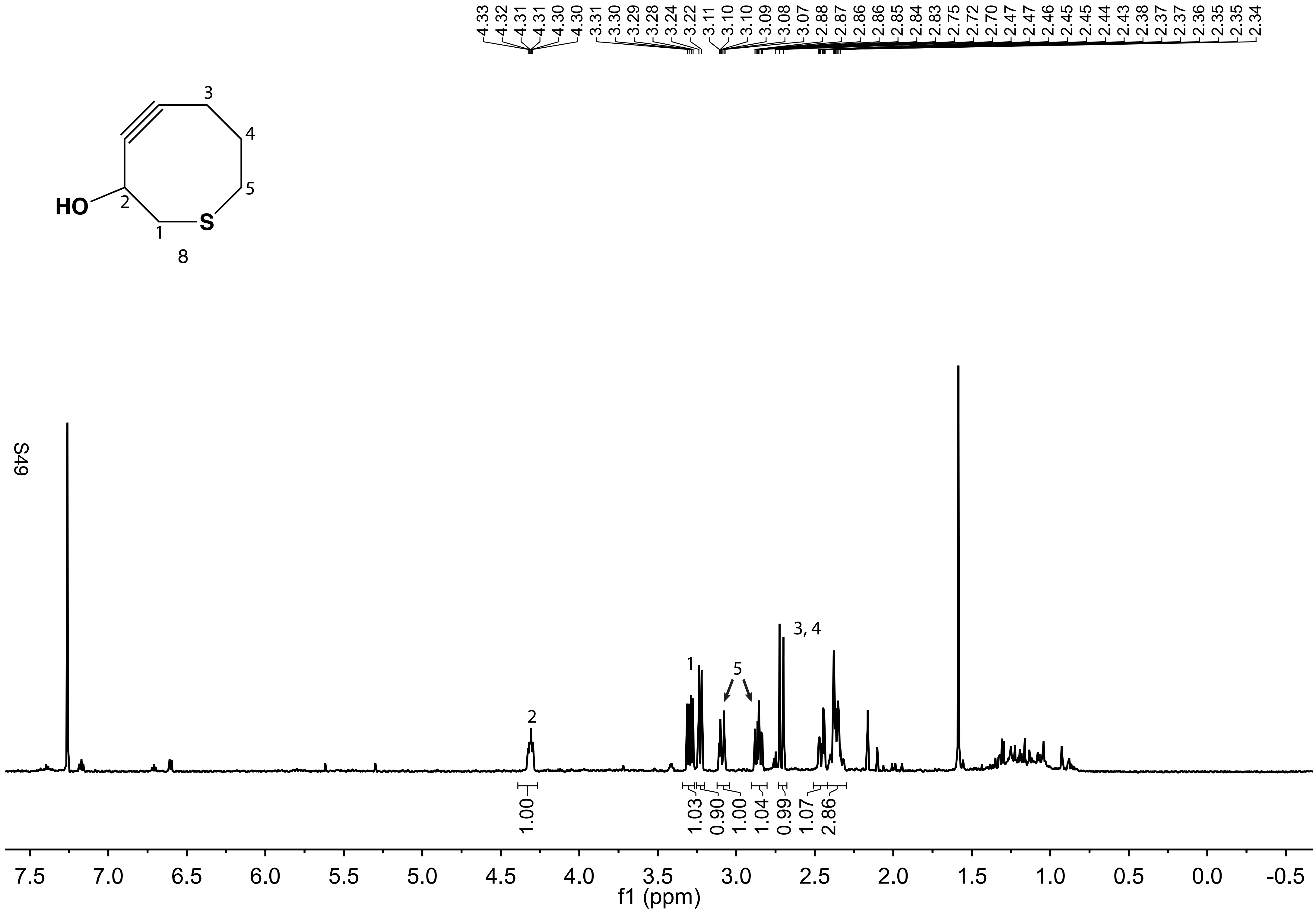
HO
S
1
2
3
4
5
8
4.33 4.32 4.31 4.31 4.30 4.30 3.31 3.30 3.29 3.28 3.24 3.22 3.11 3.10 3.10 3.09 3.08 3.07 2.88 2.87 2.86 2.86 2.85 2.84 2.83 2.75 2.72 2.70 2.47 2.47 2.46 2.45 2.45 2.44 2.43 2.38 2.37 2.37 2.36 2.35 2.35 2.34
2
1
5
3, 4
1.00
1.03
0.90
1.00
1.04
0.99
1.07
2.86
7.5 7.0 6.5 6.0 5.5 5.0 4.5 4.0 3.5 3.0 2.5 2.0 1.5 1.0 0.5 0.0 -0.5
f1 (ppm)

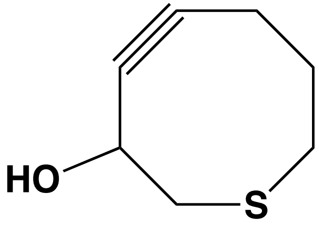

8

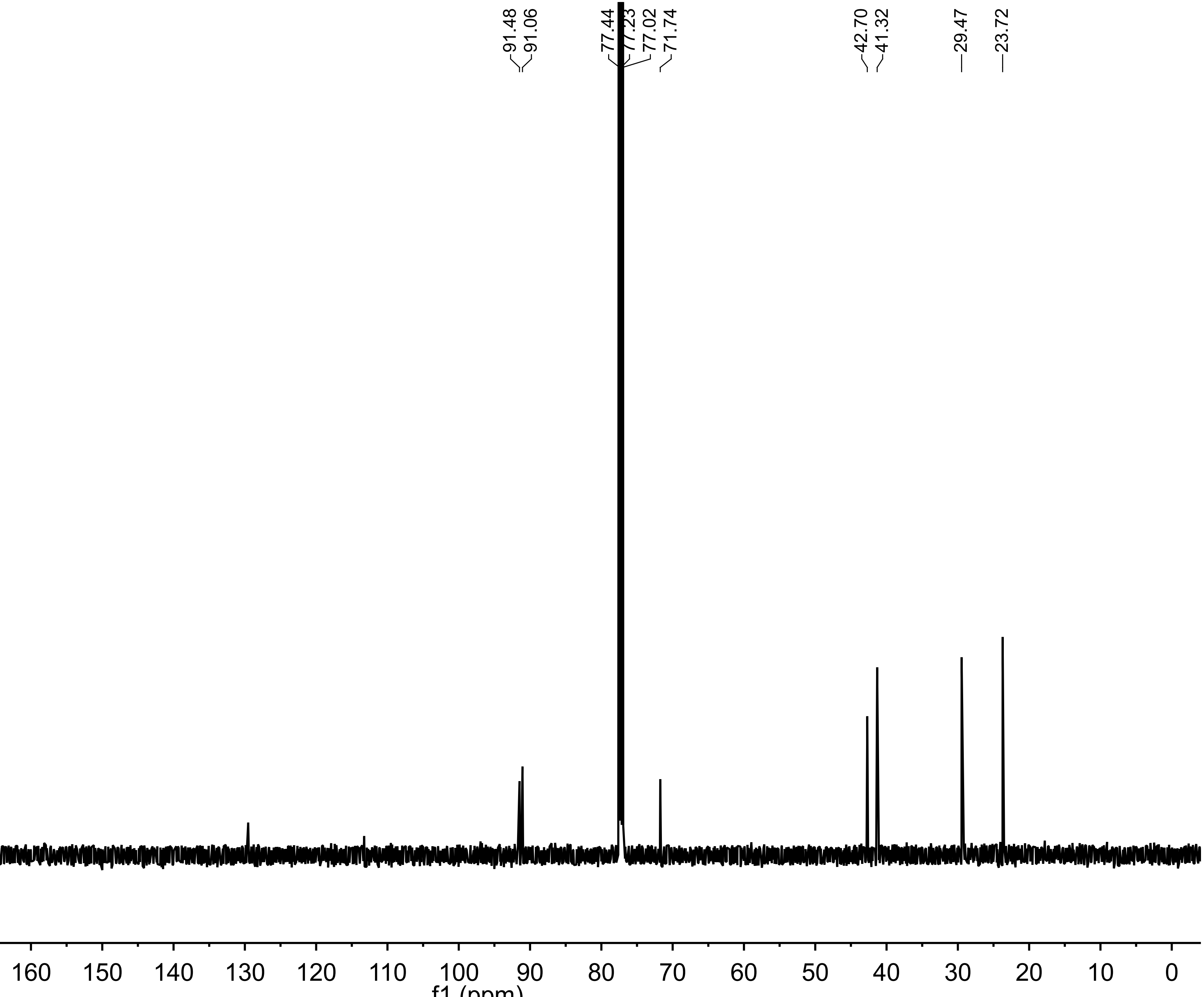
91.48
91.06
77.44
77.23
77.02
71.74
42.70
41.32
29.47
23.72
190 180 170 160 150 140 130 120 110 100 90 80 70 60 50 40 30 20 10 0
f1 (ppm)

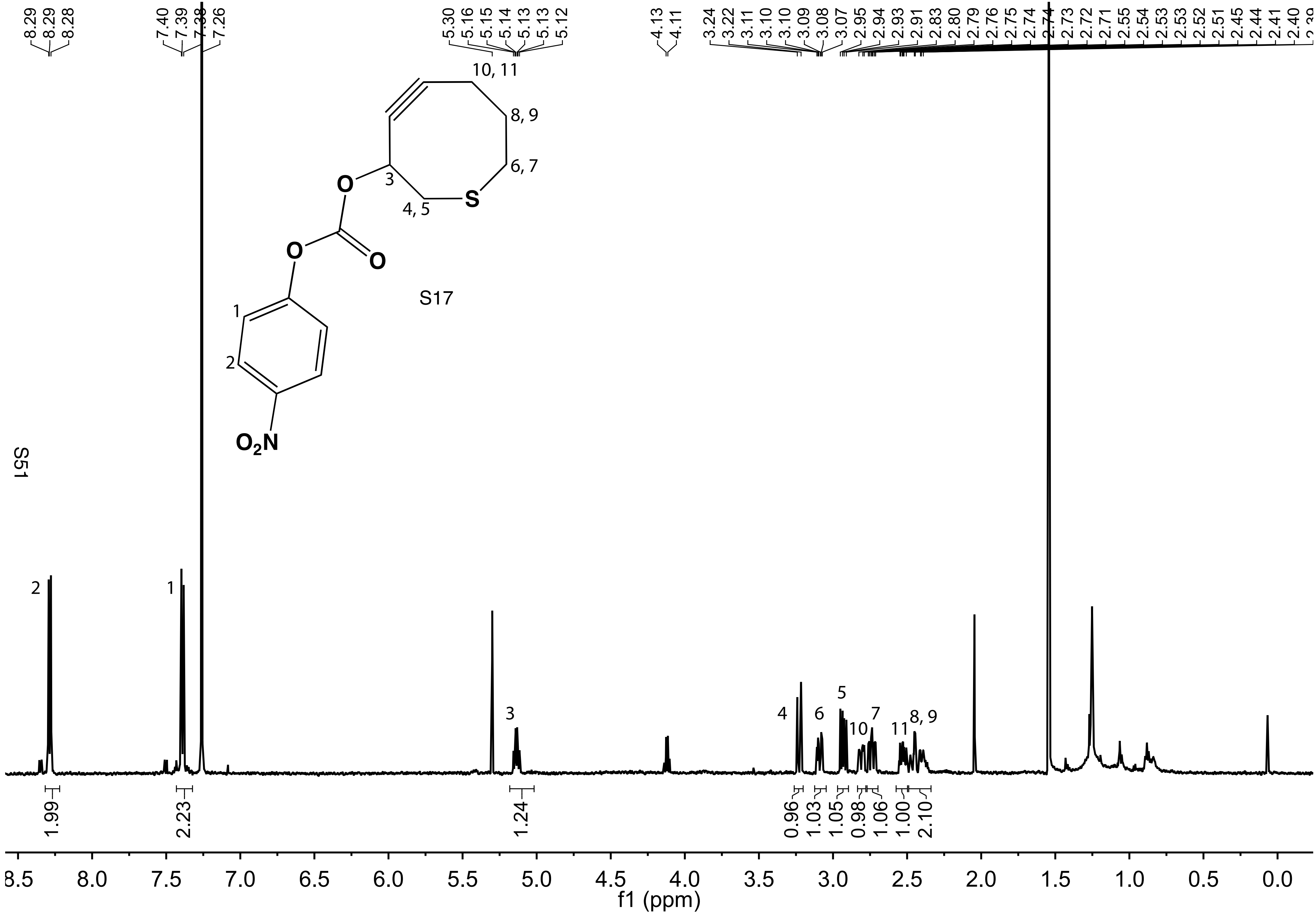
8.29
8.29
8.28
7.40
7.39
7.26
5.30
5.16
5.15
5.14
5.13
5.13
5.12
4.13
4.11
3.24
3.22
3.11
3.10
3.10
3.09
3.08
3.07
2.95
2.94
2.93
2.91
2.83
2.80
2.79
2.76
2.75
2.74
2.73
2.72
2.71
2.55
2.54
2.53
2.53
2.52
2.51
2.45
2.44
2.41
2.40
10, 11
8, 9
6, 7
3
4, 5
S
O
O
O
1
2
O_2N
S17
2
1
3
4
6
5
10
7
11
8, 9
1.99
2.23
1.24
0.96
1.03
1.05
0.98
1.06
1.00
2.10
8.0
7.5
7.0
6.5
6.0
5.5
5.0
4.5
4.0
3.5
3.0
2.5
2.0
1.5
1.0
0.5
0.0
f1 (ppm)

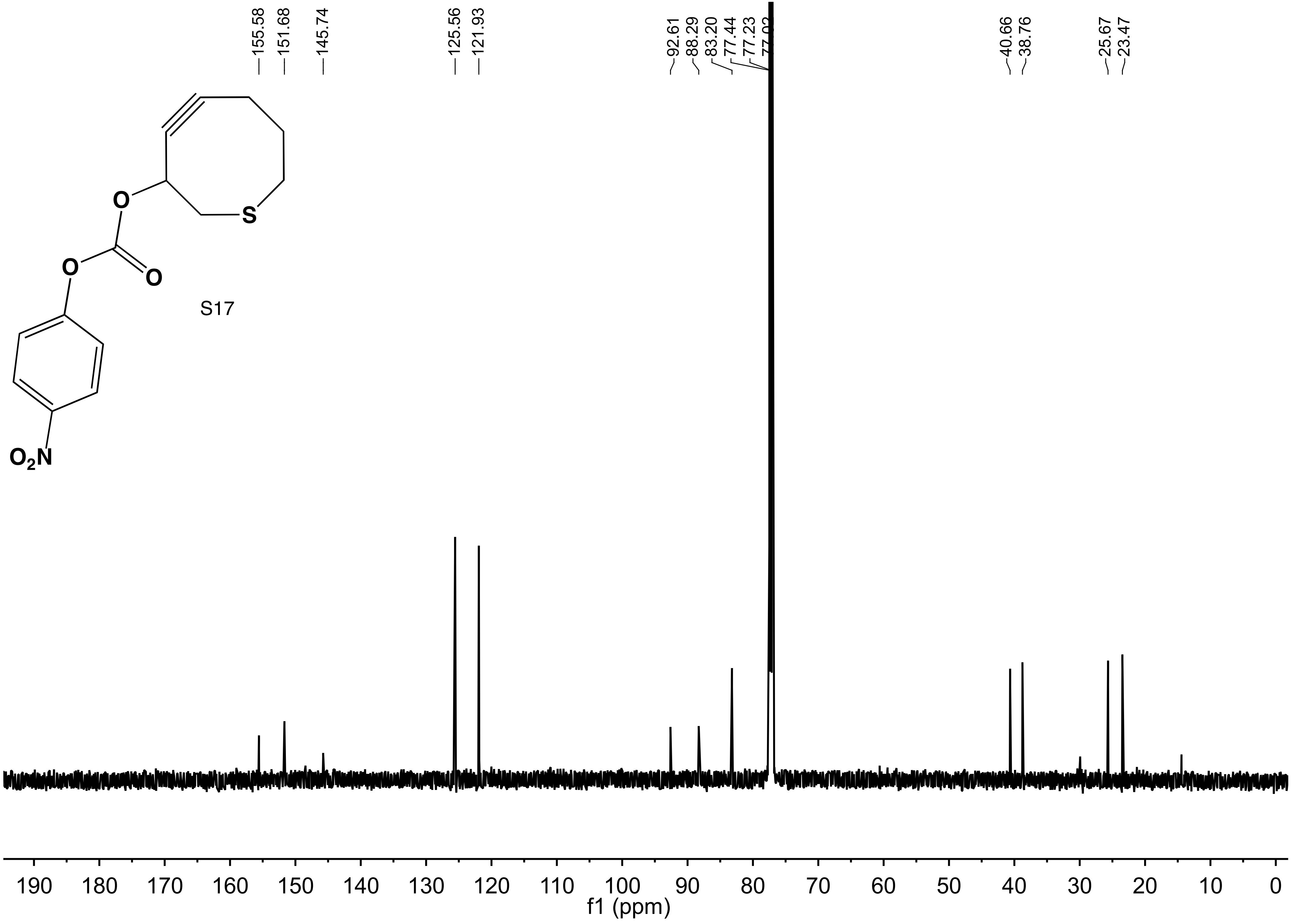
155.58
151.68
145.74
125.56
121.93
92.61
88.29
83.20
77.44
77.23
77.02
40.66
38.76
25.67
23.47
O
O
O
S
O₂N
S17
190 180 170 160 150 140 130 120 110 100 90 80 70 60 50 40 30 20 10 0
f1 (ppm)

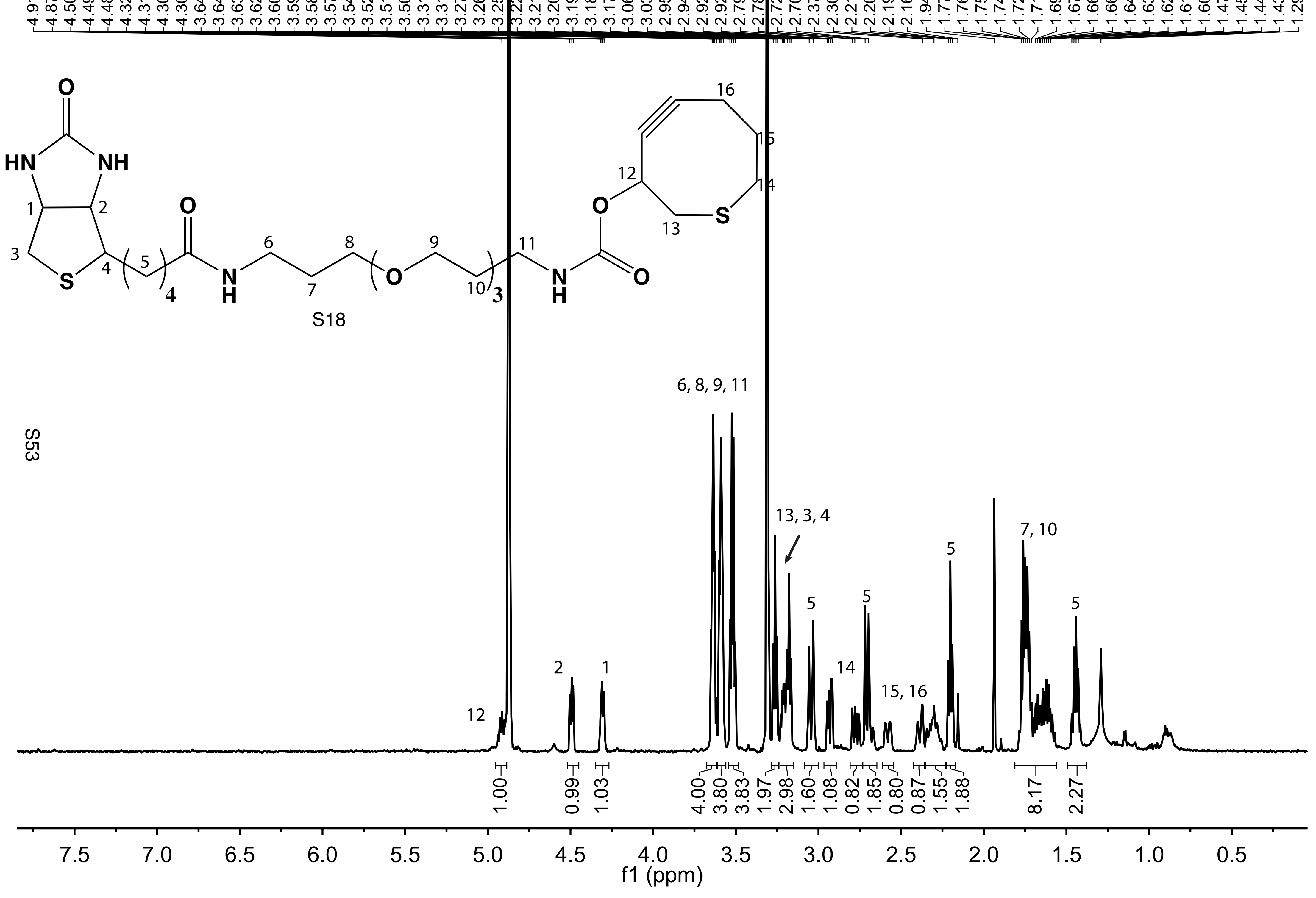
4.91 4.87 4.50 4.49 4.48 4.32 4.31 4.30 4.30 3.64 3.64 3.63 3.62 3.60 3.59 3.58 3.57 3.54 3.52 3.51 3.50 3.31 3.31 3.27 3.26 3.25 3.22 3.21 3.20 3.19 3.18 3.17 3.06 3.03 2.95 2.94 2.92 2.92 2.79 2.78 2.72 2.70 2.37 2.30 2.21 2.20 2.19 2.16 1.94 1.77 1.76 1.75 1.74 1.72 1.71 1.69 1.67 1.66 1.66 1.64 1.63 1.62 1.61 1.60 1.47 1.45 1.44 1.43 1.29
S18
12
2
1
6, 8, 9, 11
13, 3, 4
5
14
5
15, 16
5
7, 10
5
1.00
0.99
1.03
4.00
3.80
3.83
1.97
2.98
1.60
1.08
0.82
1.85
0.80
0.87
1.55
1.88
8.17
2.27
f1 (ppm)

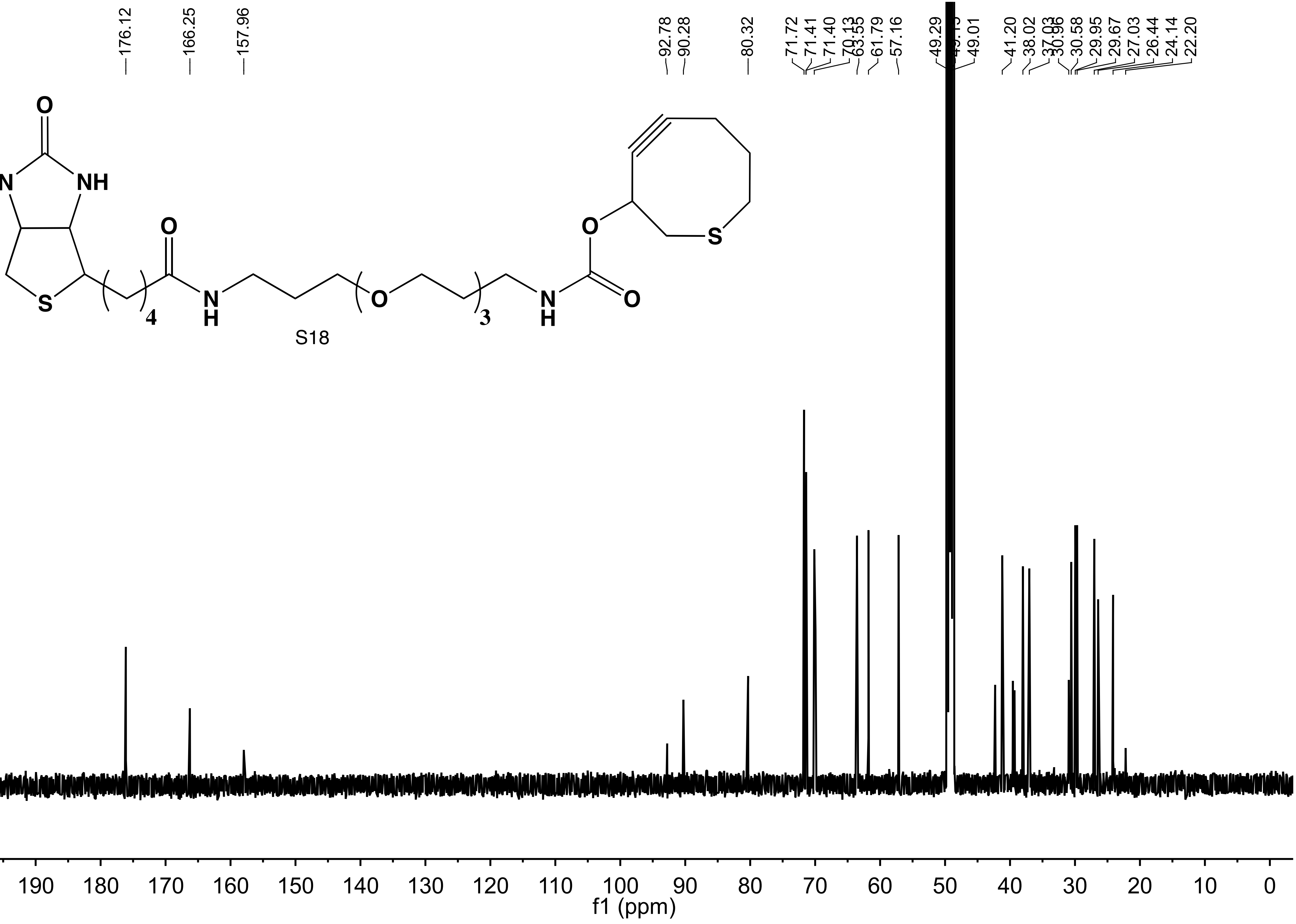
176.12
166.25
157.96
92.78
90.28
80.32
71.72
71.41
71.40
70.13
63.55
61.79
57.16
49.29
49.01
41.20
38.02
37.03
30.96
30.58
29.95
29.67
27.03
26.44
24.14
22.20
S18
190 180 170 160 150 140 130 120 110 100 90 80 70 60 50 40 30 20 10 0
f1 (ppm)

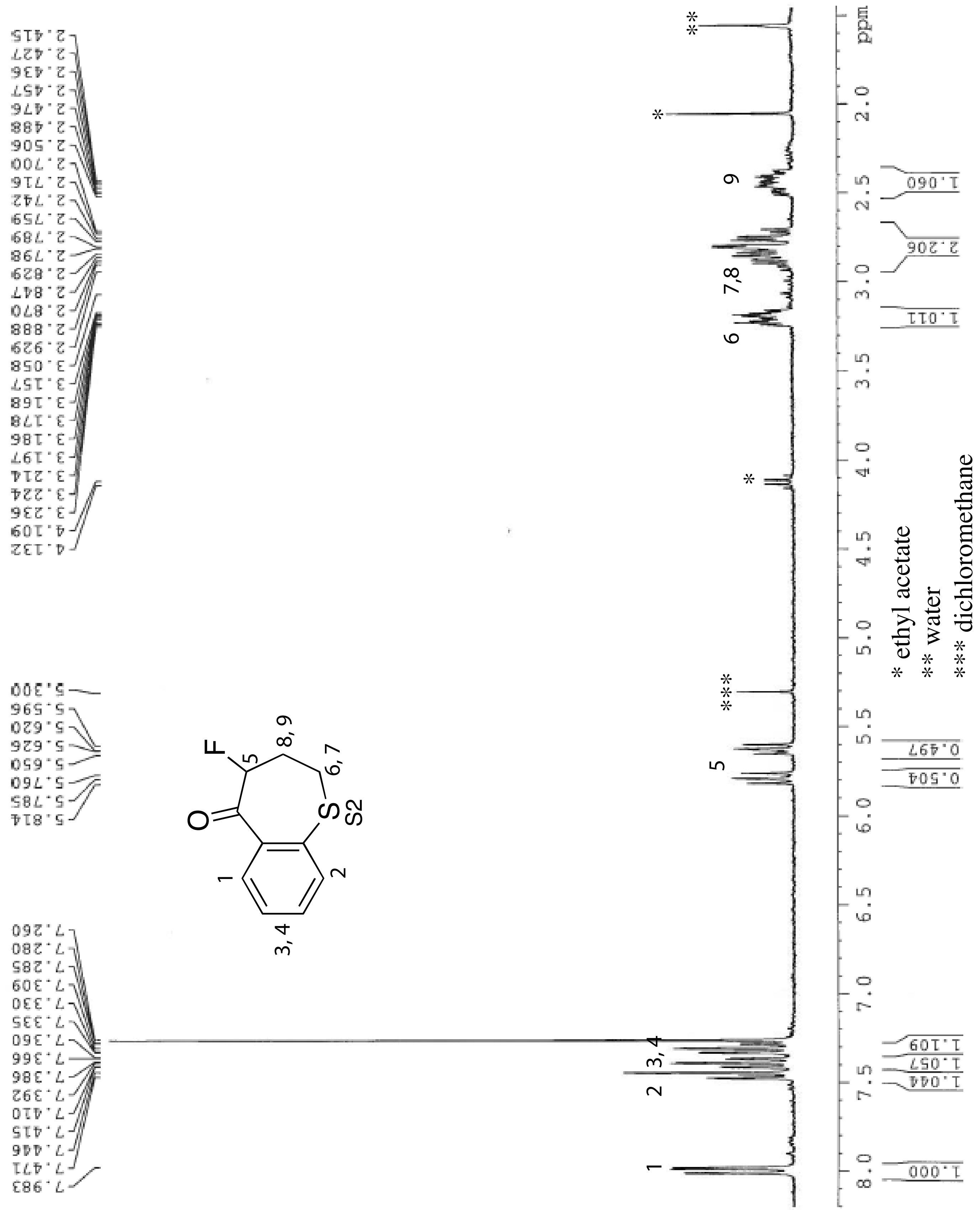

S2
* ethyl acetate
** water
*** dichloromethane
ppm

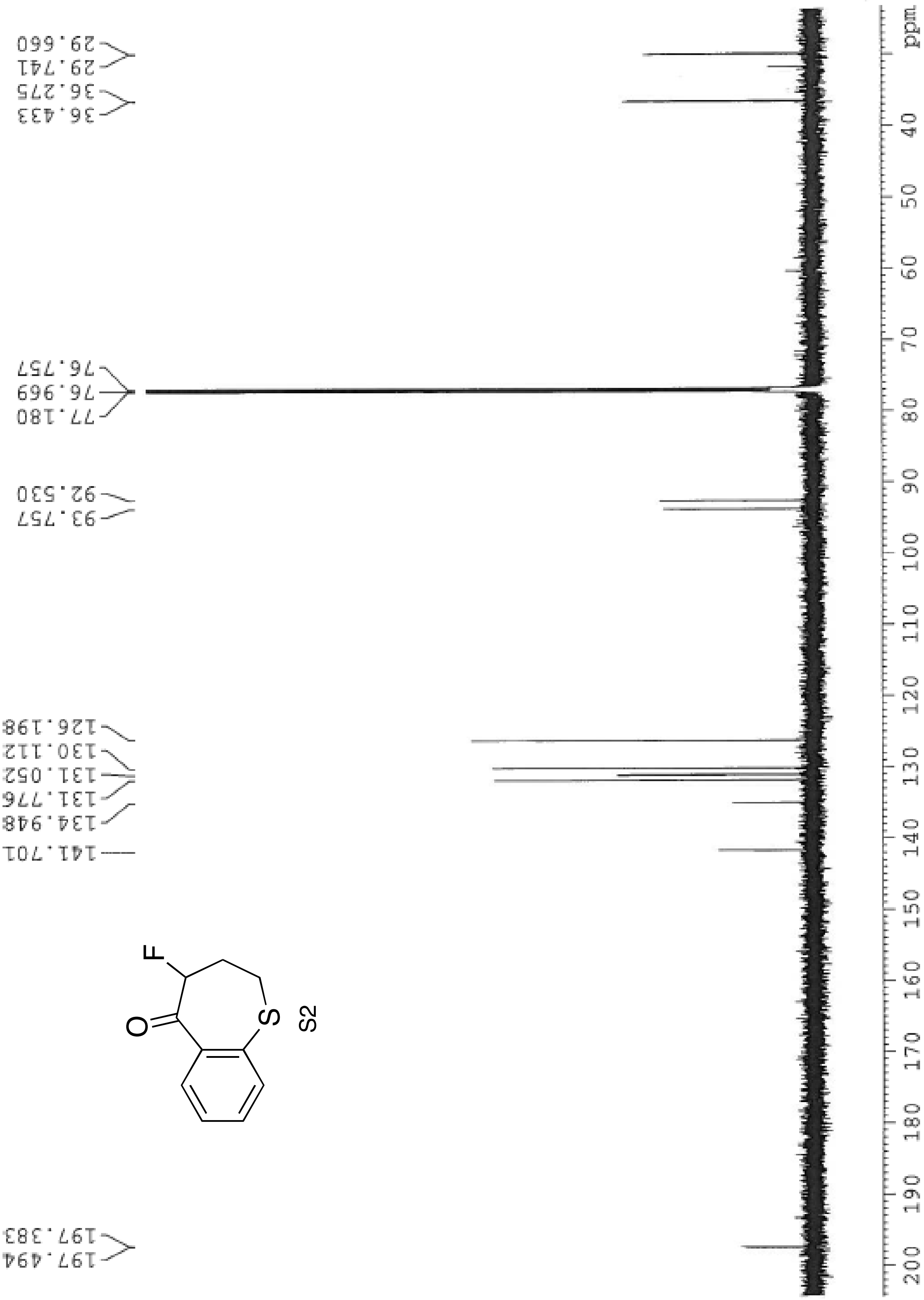

29.660
29.741
36.275
36.433
76.757
76.969
77.180
92.530
93.757
126.198
130.112
131.052
131.776
134.948
141.701
197.383
197.494
O
F
S
S2
ppm

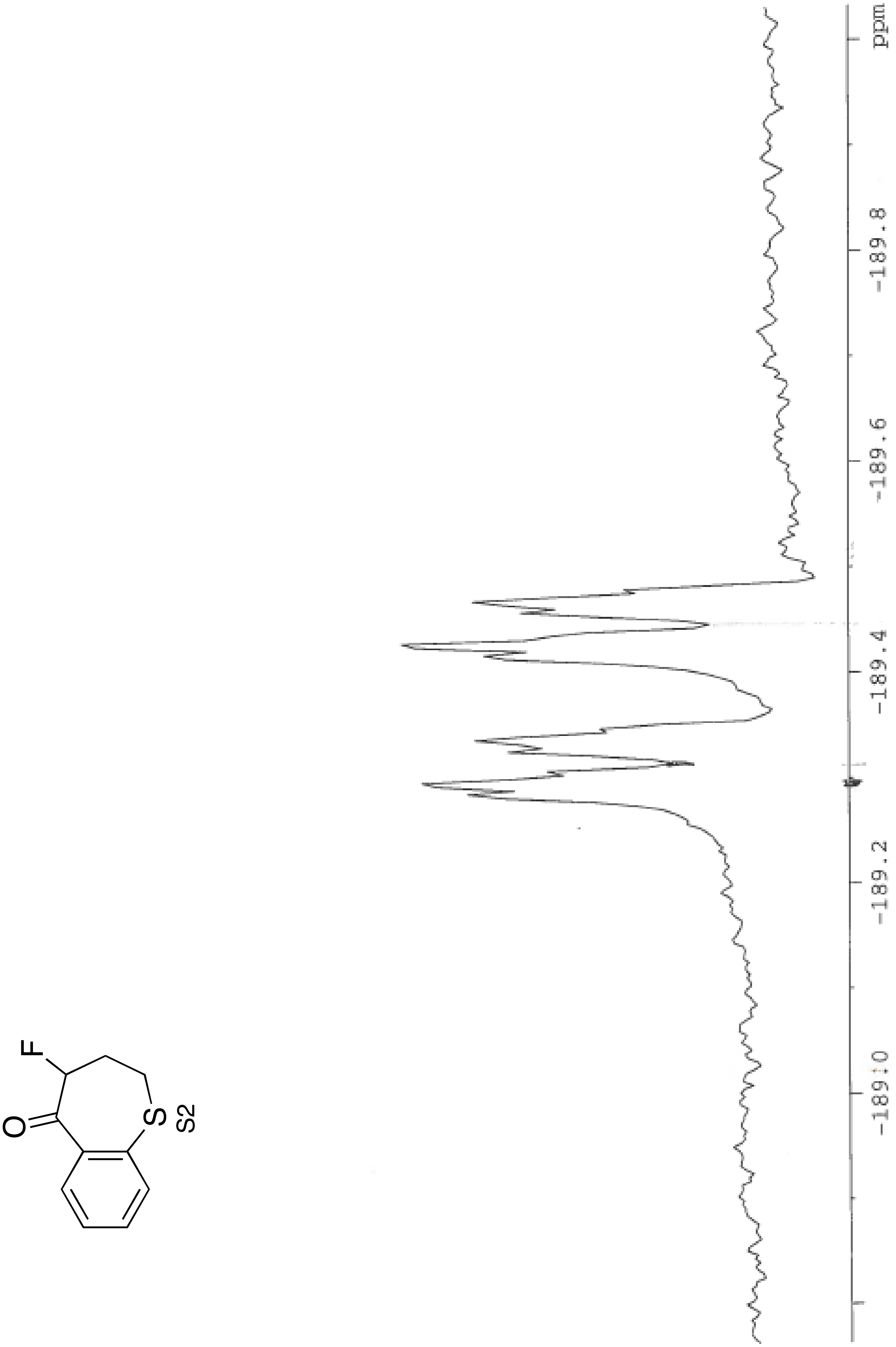

S2

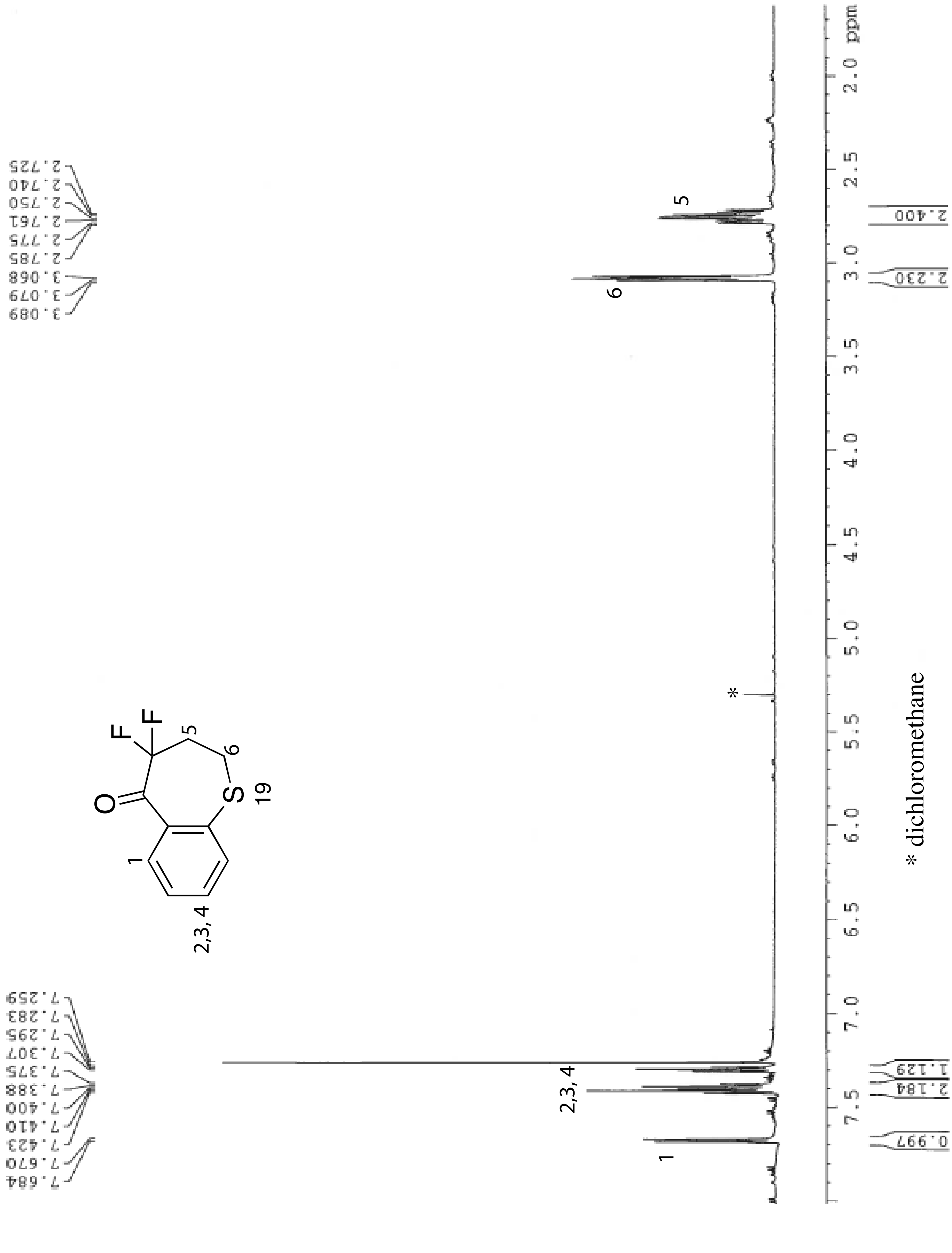
7.684
7.670
7.423
7.410
7.400
7.388
7.375
7.307
7.295
7.283
7.259
3.089
3.079
3.068
2.785
2.775
2.761
2.750
2.740
2.725
O
F
F
S
1
2,3, 4
5
6
19
1
2,3, 4
6
5
*
* dichloromethane
0.997
2.184
1.129
2.230
2.400
7.5
7.0
6.5
6.0
5.5
5.0
4.5
4.0
3.5
3.0
2.5
2.0
ppm

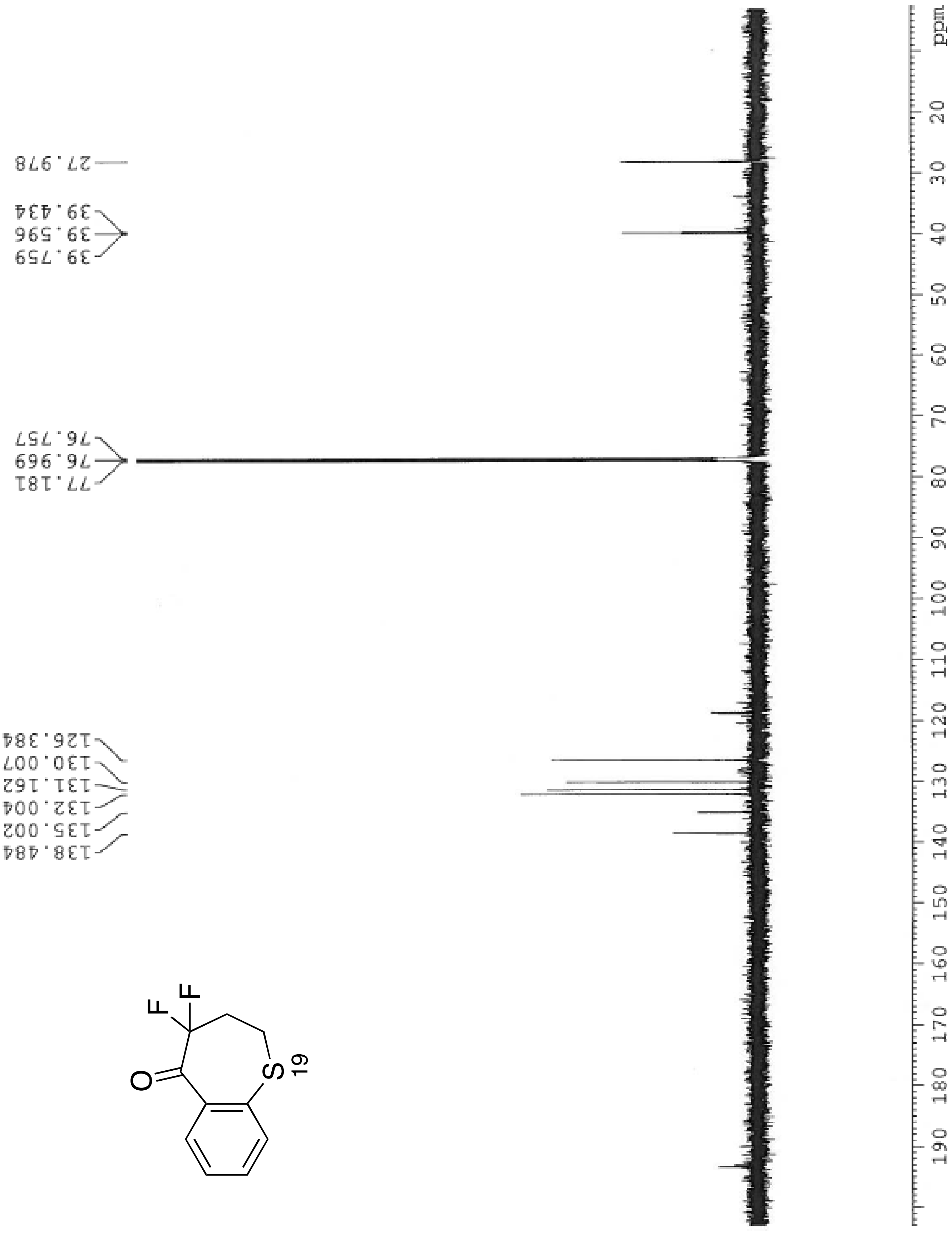
27.978
39.434
39.596
39.759
76.757
76.969
77.181
126.384
130.007
131.162
132.004
135.002
138.484
ppm
O
F
F
S
19

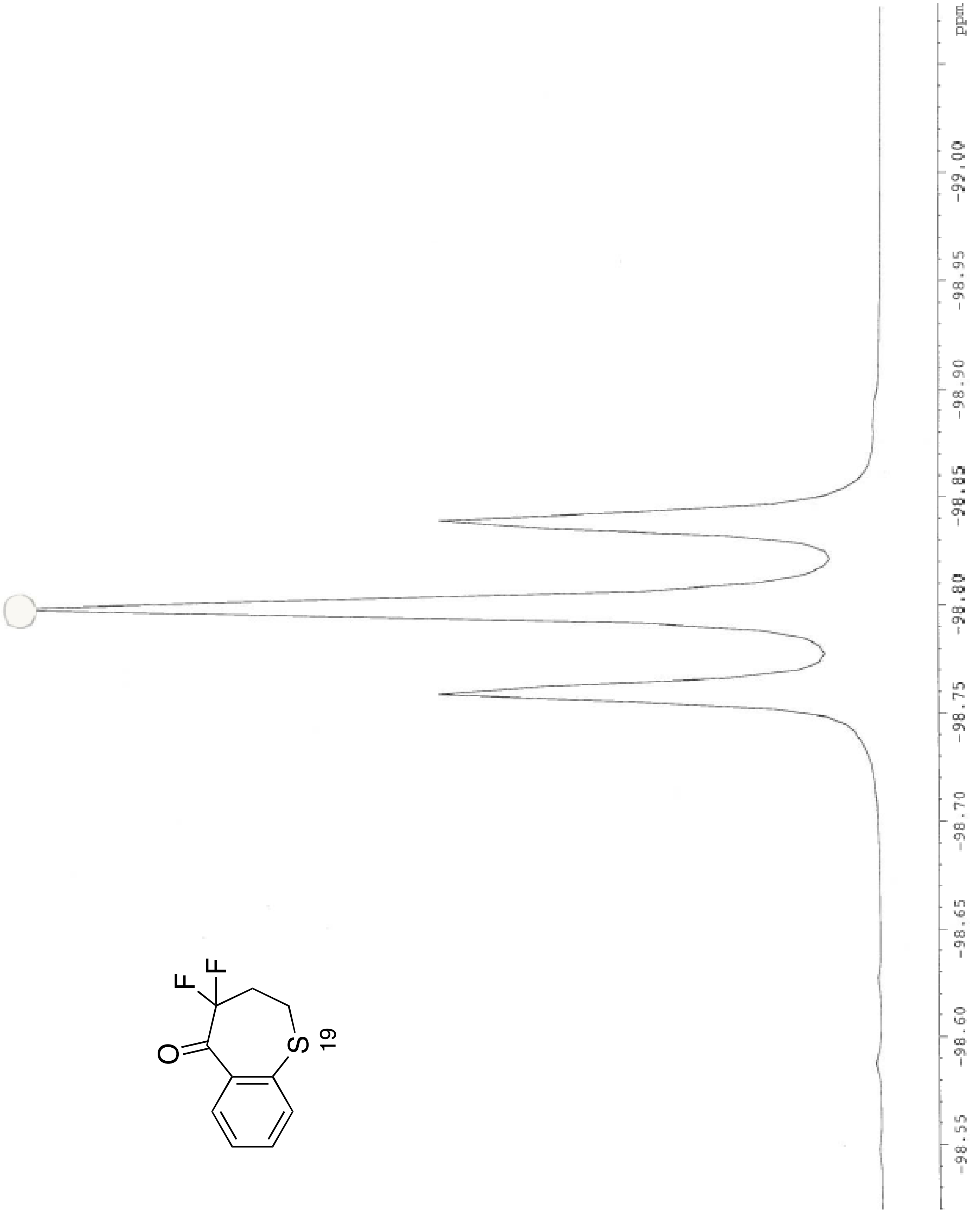
19
ppm
-99.00
-98.95
-98.90
-98.85
-98.80
-98.75
-98.70
-98.65
-98.60
-98.55

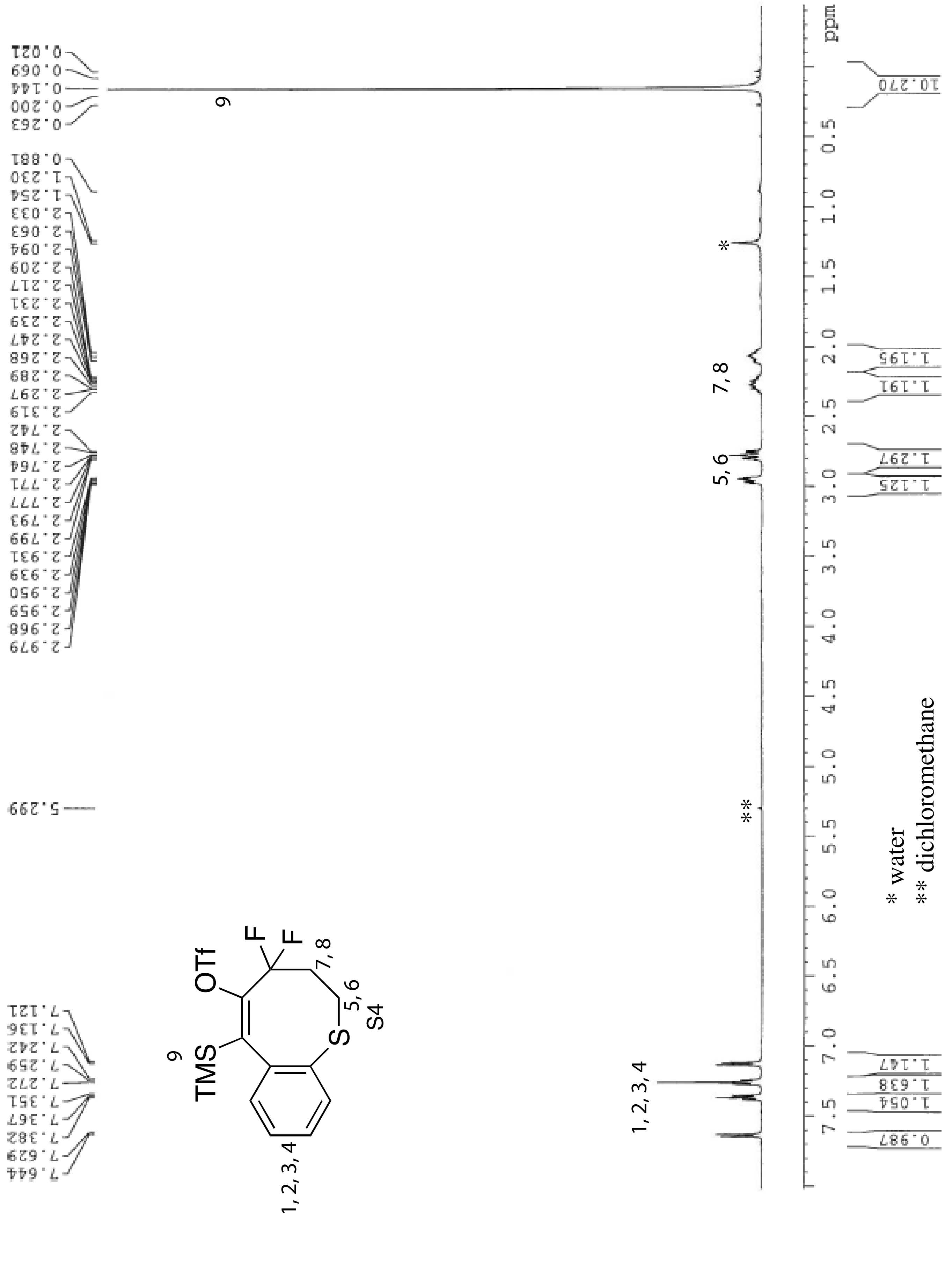

TMS
OTf
F
F
S
S4
9
1, 2, 3, 4
5, 6
7, 8
* water
** dichloromethane
ppm

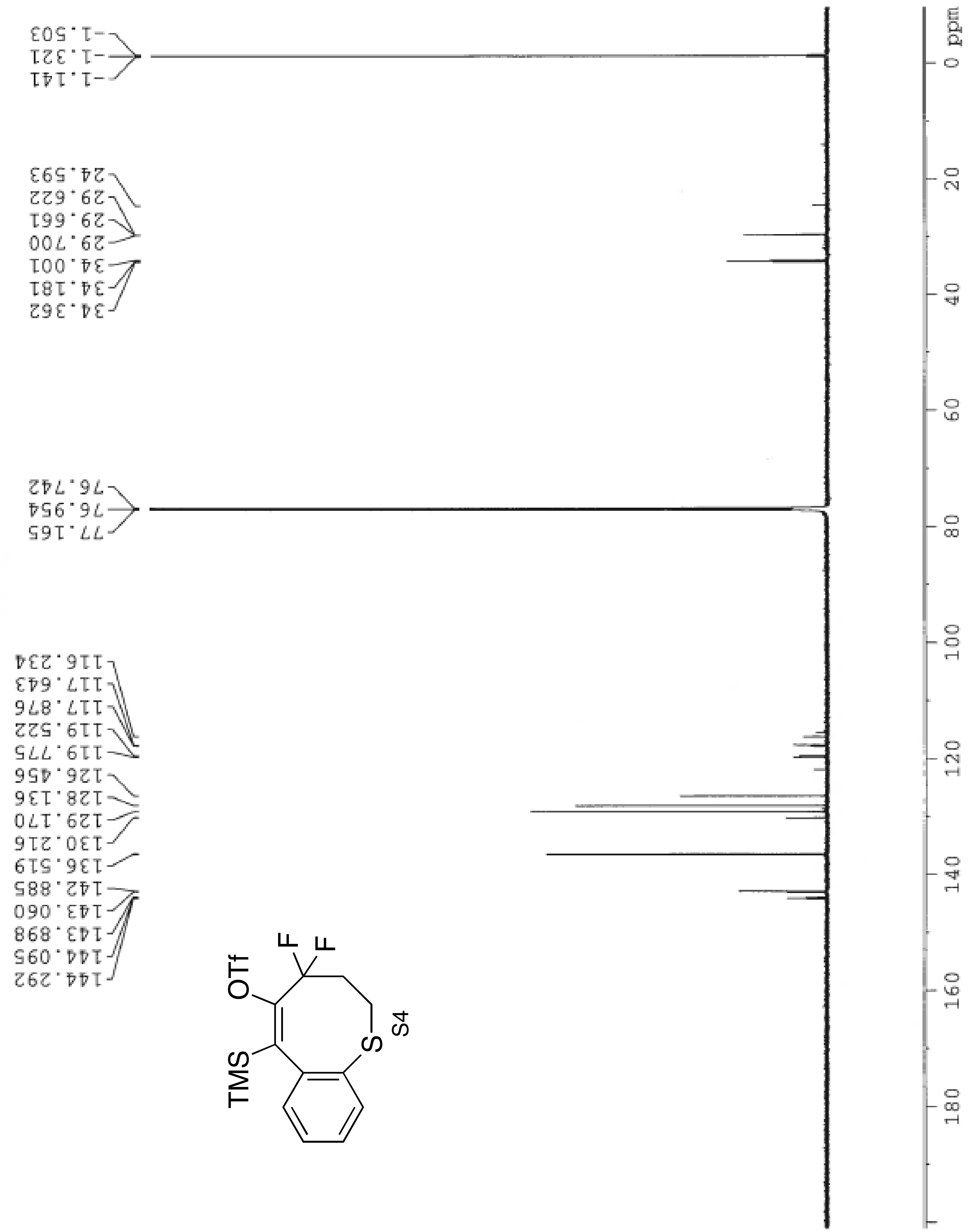
-1.503
-1.321
-1.141
24.593
29.622
29.661
29.700
34.001
34.181
34.362
76.742
76.954
77.165
116.234
117.643
117.876
119.522
119.775
126.456
128.136
129.170
130.216
136.519
142.885
143.060
143.898
144.095
144.292
TMS
OTf
F
F
S
S4
0
20
40
60
80
100
120
140
160
180
ppm

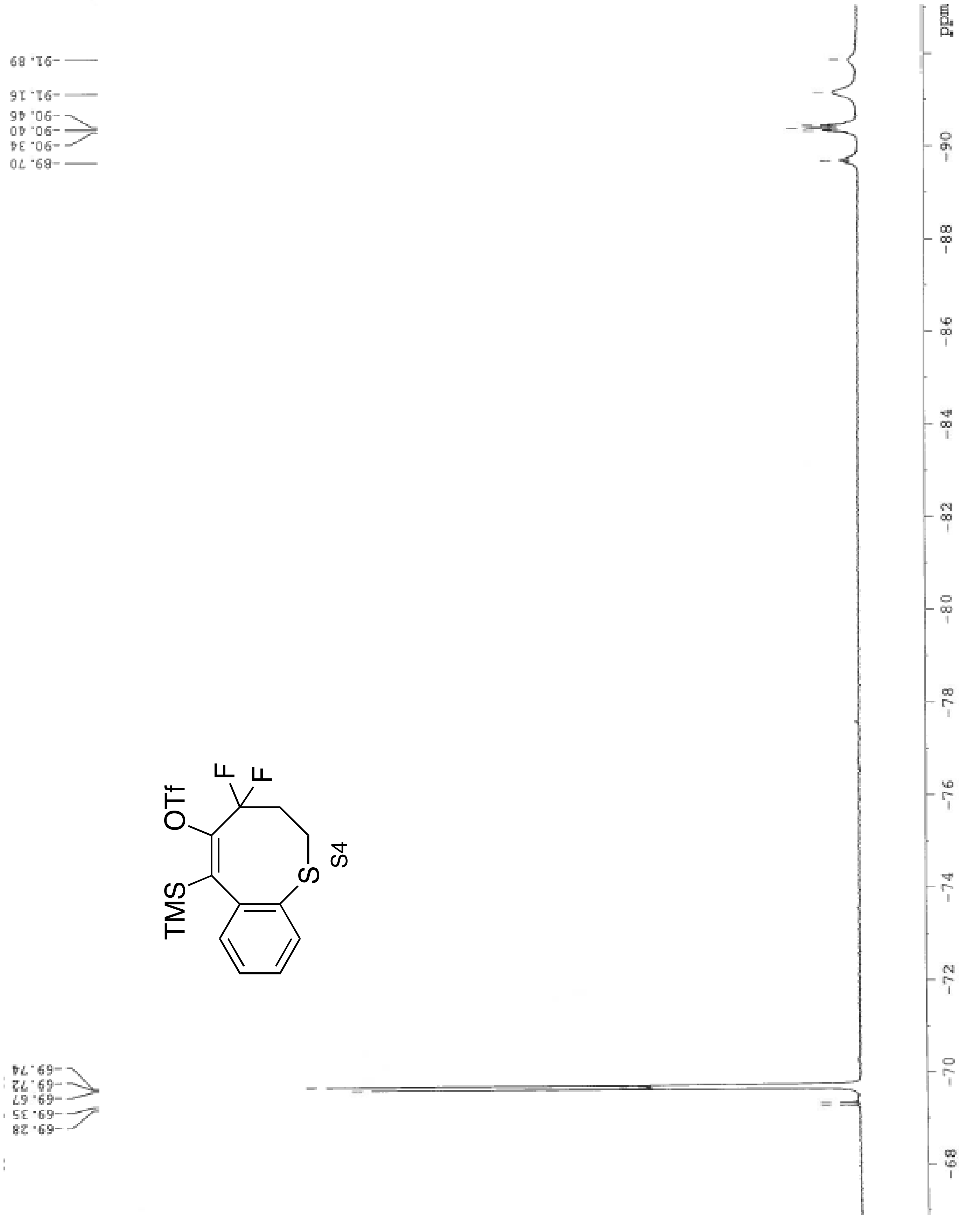
-91.89
-91.16
-90.46
-90.40
-90.34
-89.70
-69.74
-69.72
-69.67
-69.35
-69.28
TMS
OTf
F
F
S
S4
ppm
-90
-88
-86
-84
-82
-80
-78
-76
-74
-72
-70
-68

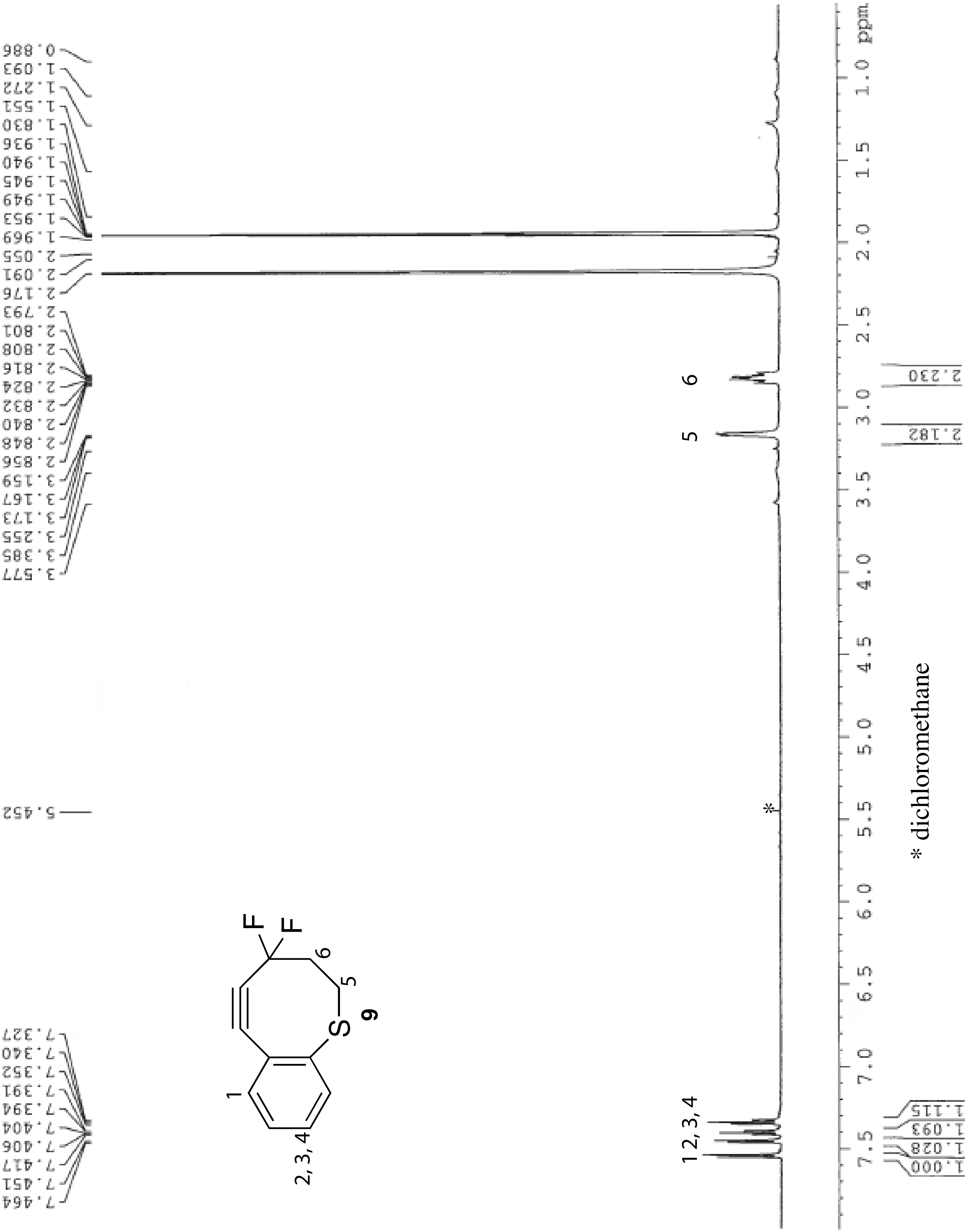
F
F
S
9
1
2, 3, 4
5
6
1 2, 3, 4
* dichloromethane
ppm

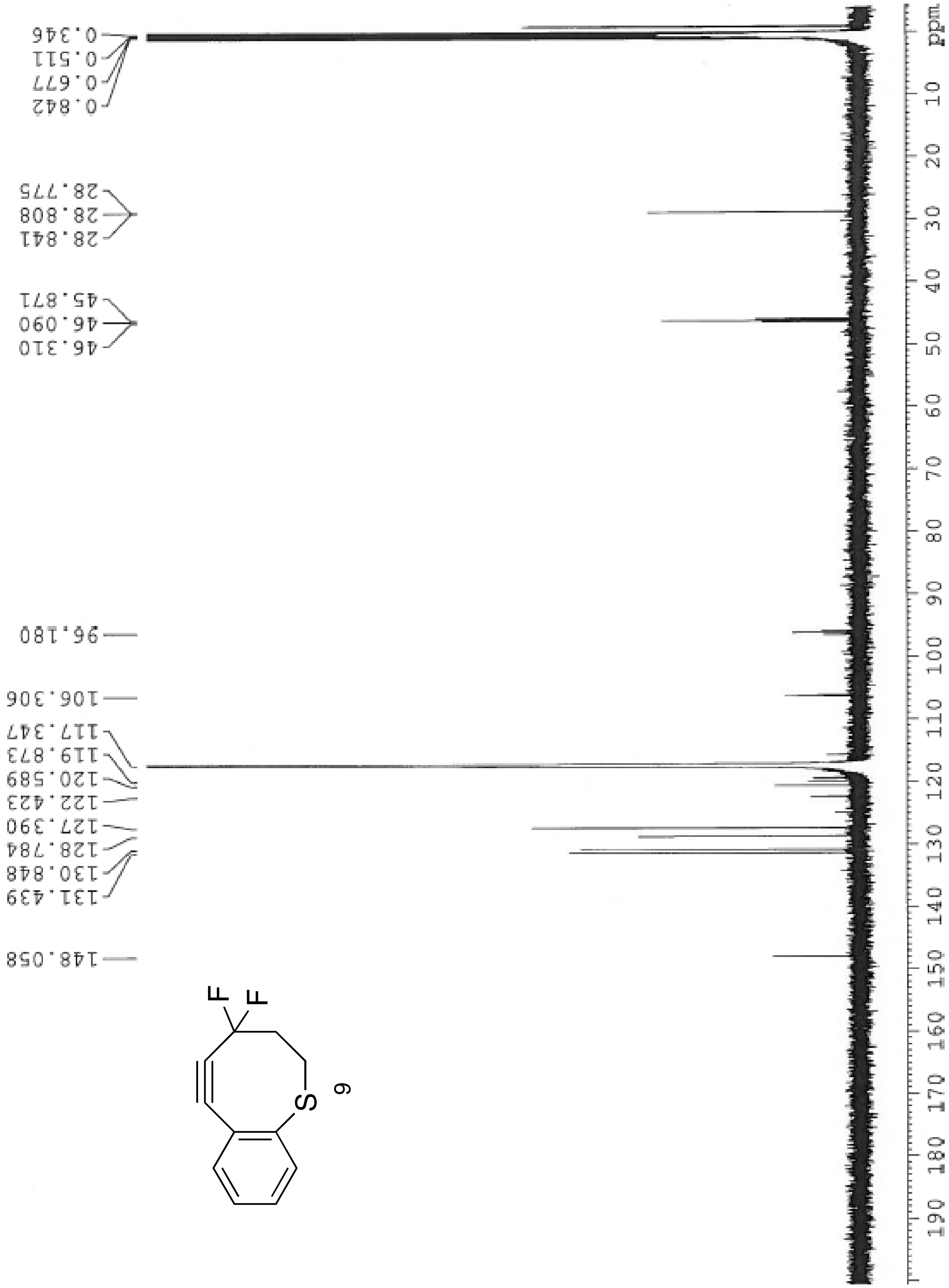
0.346
0.511
0.677
0.842
28.775
28.808
28.841
45.871
46.090
46.310
96.180
106.306
117.347
119.873
120.589
122.423
127.390
128.784
130.848
131.439
148.058
F
F
S
ppm
10 20 30 40 50 60 70 80 90 100 110 120 130 140 150 160 170 180 190

9

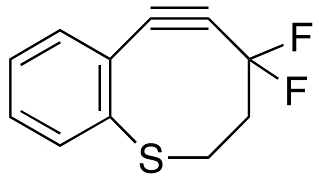

9

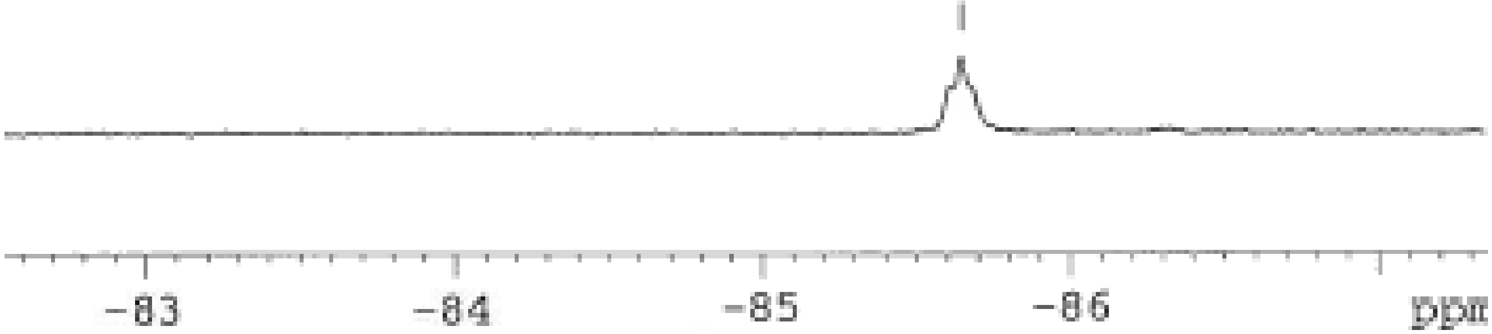

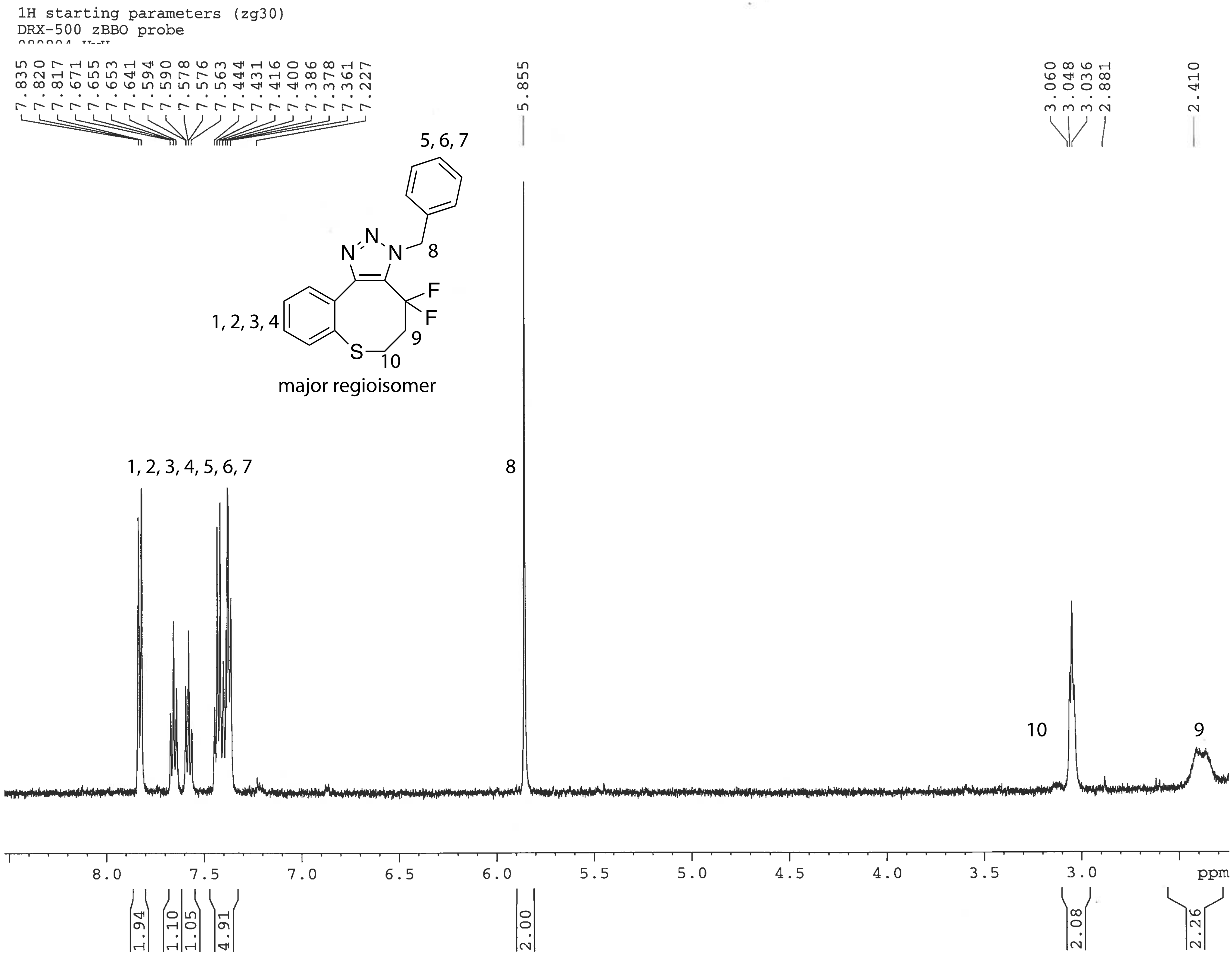
1H starting parameters (zg30)
DRX-500 zBBO probe
7.835
7.820
7.817
7.671
7.655
7.653
7.641
7.594
7.590
7.578
7.576
7.563
7.444
7.431
7.416
7.400
7.386
7.378
7.361
7.227
5.855
3.060
3.048
3.036
2.881
2.410
5, 6, 7
8
N
N
N
F
F
9
10
S
1, 2, 3, 4
major regioisomer
1, 2, 3, 4, 5, 6, 7
8
10
9
8.0
7.5
7.0
6.5
6.0
5.5
5.0
4.5
4.0
3.5
3.0
ppm
1.94
1.10
1.05
4.91
2.00
2.08
2.26

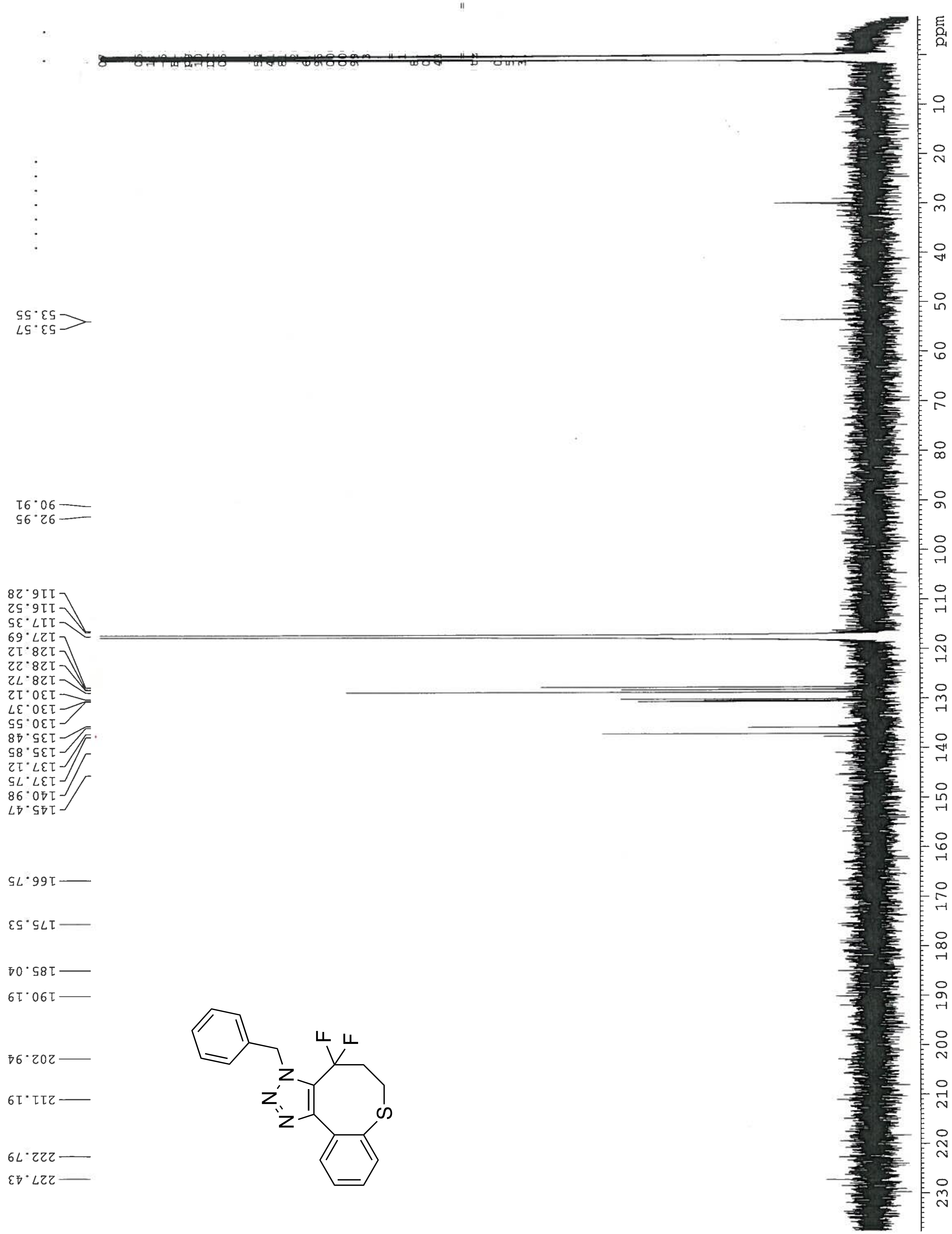
53.55
53.57
90.91
92.95
116.28
116.52
117.35
127.69
128.12
128.22
128.72
130.12
130.37
130.55
135.48
135.85
137.12
137.75
140.98
145.47
166.75
175.53
185.04
190.19
202.94
211.19
222.79
227.43
ppm
F
F
N
N
N
S

AV-600 ZBO proton starting parameters 11/16/08 RN

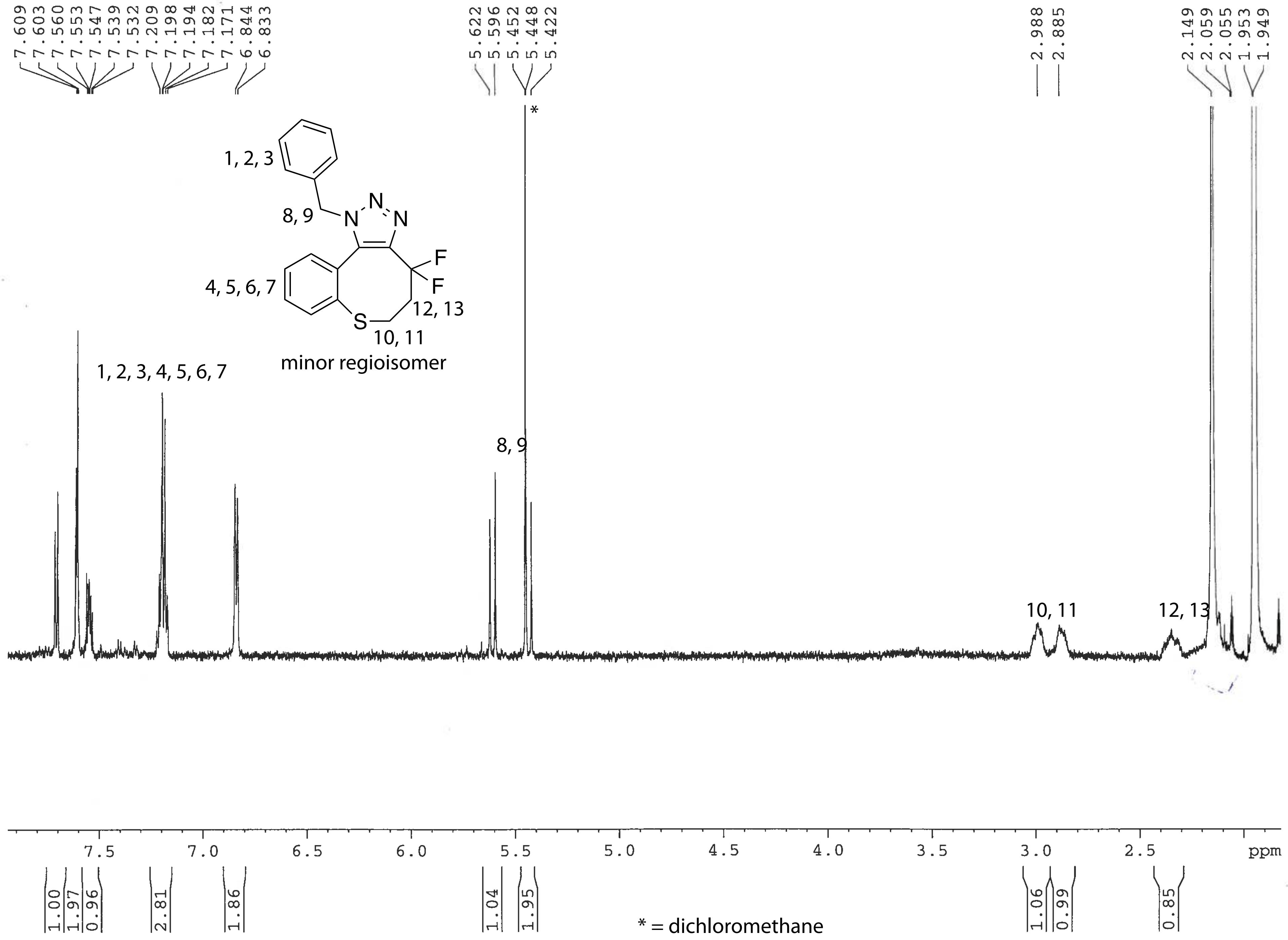

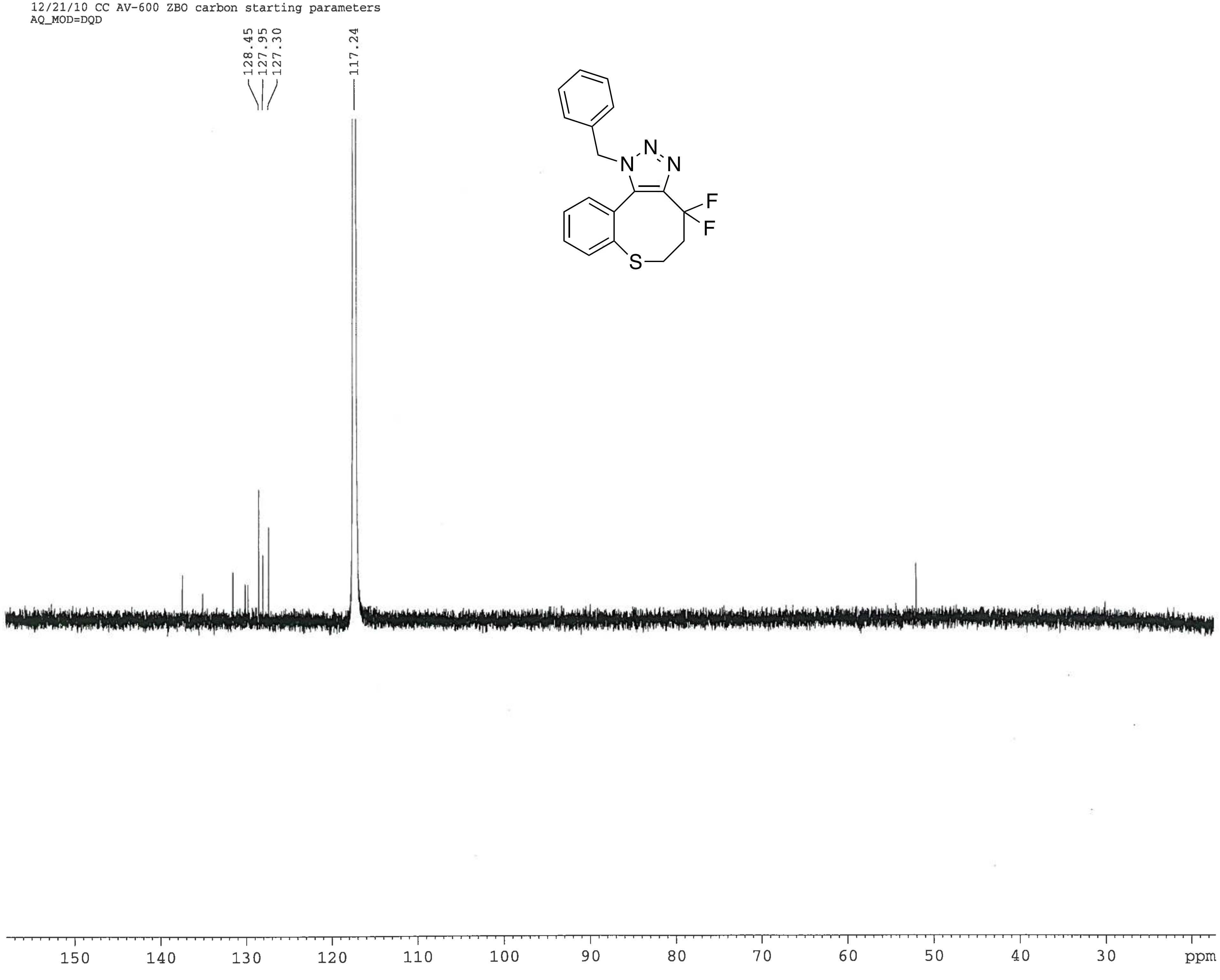
12/21/10 CC AV-600 ZBO carbon starting parameters
AQ_MOD=DQD
128.45
127.95
127.30
117.24
N
N
N
F
F
S
150
140
130
120
110
100
90
80
70
60
50
40
30
ppm

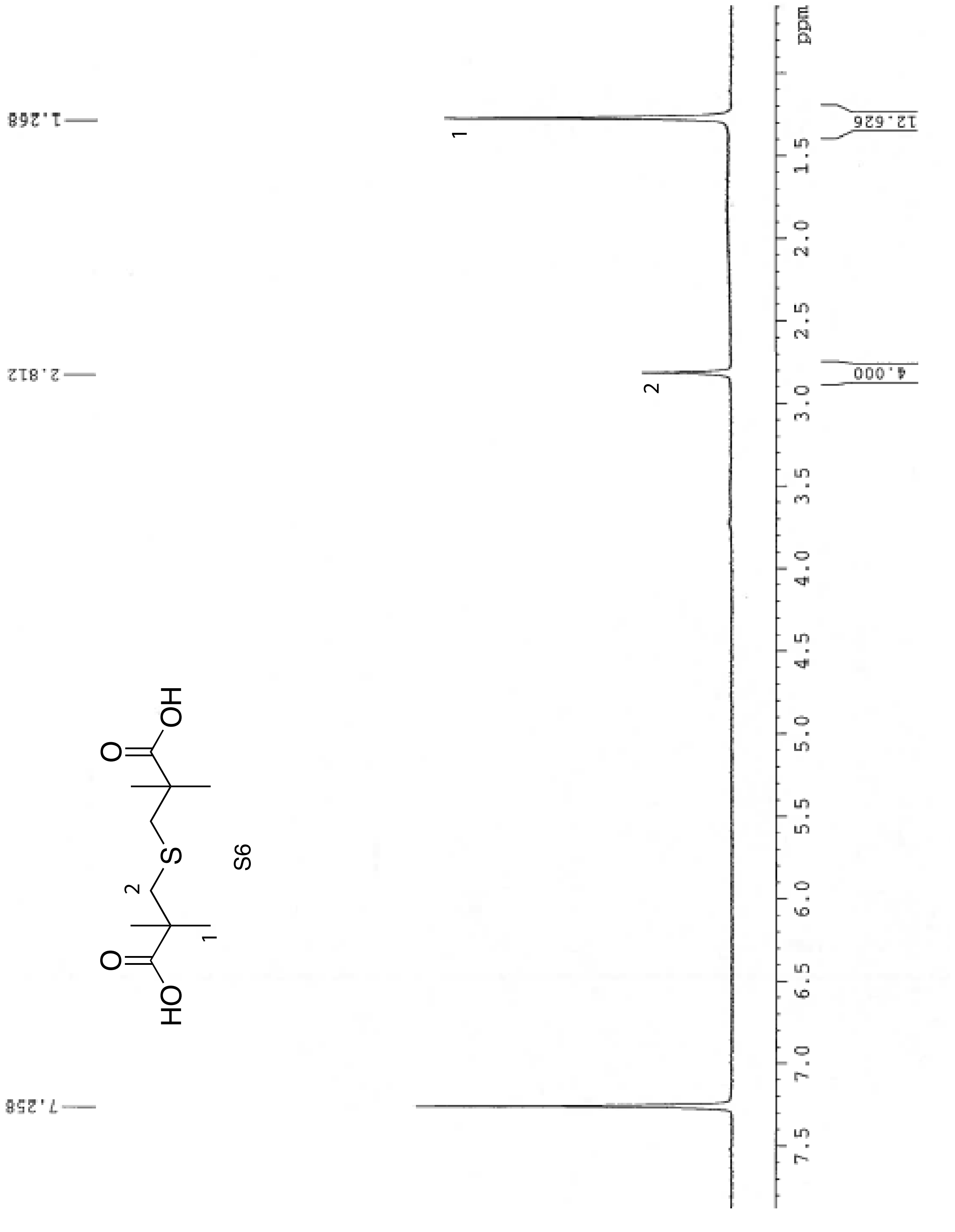

1.268
2.812
7.258
12.626
4.000
ppm
S6

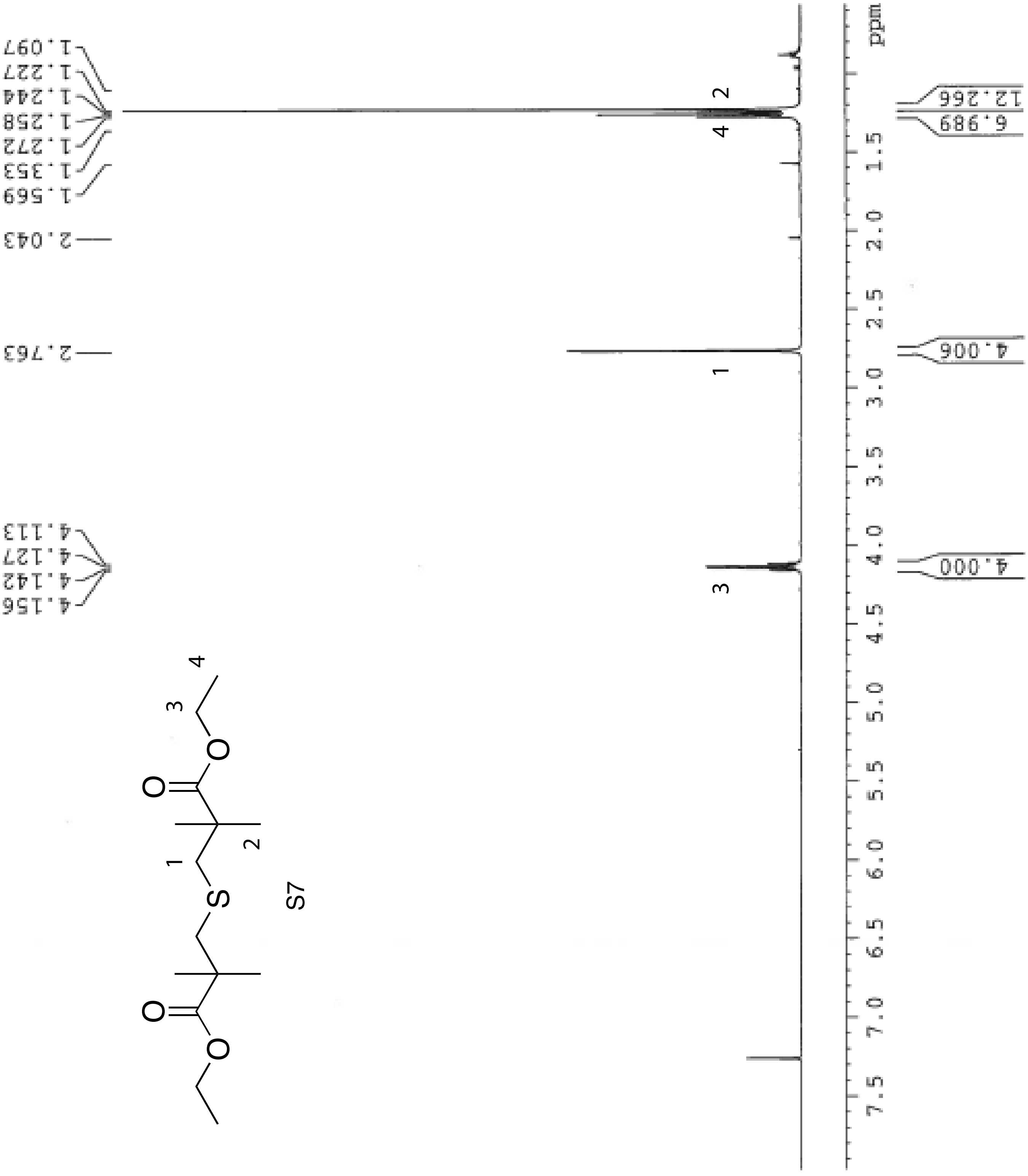
1.097
1.227
1.244
1.258
1.272
1.353
1.569
2.043
2.763
4.113
4.127
4.142
4.156
12.266
6.989
4.006
4.000
ppm
1.5
2.0
2.5
3.0
3.5
4.0
4.5
5.0
5.5
6.0
6.5
7.0
7.5
S7

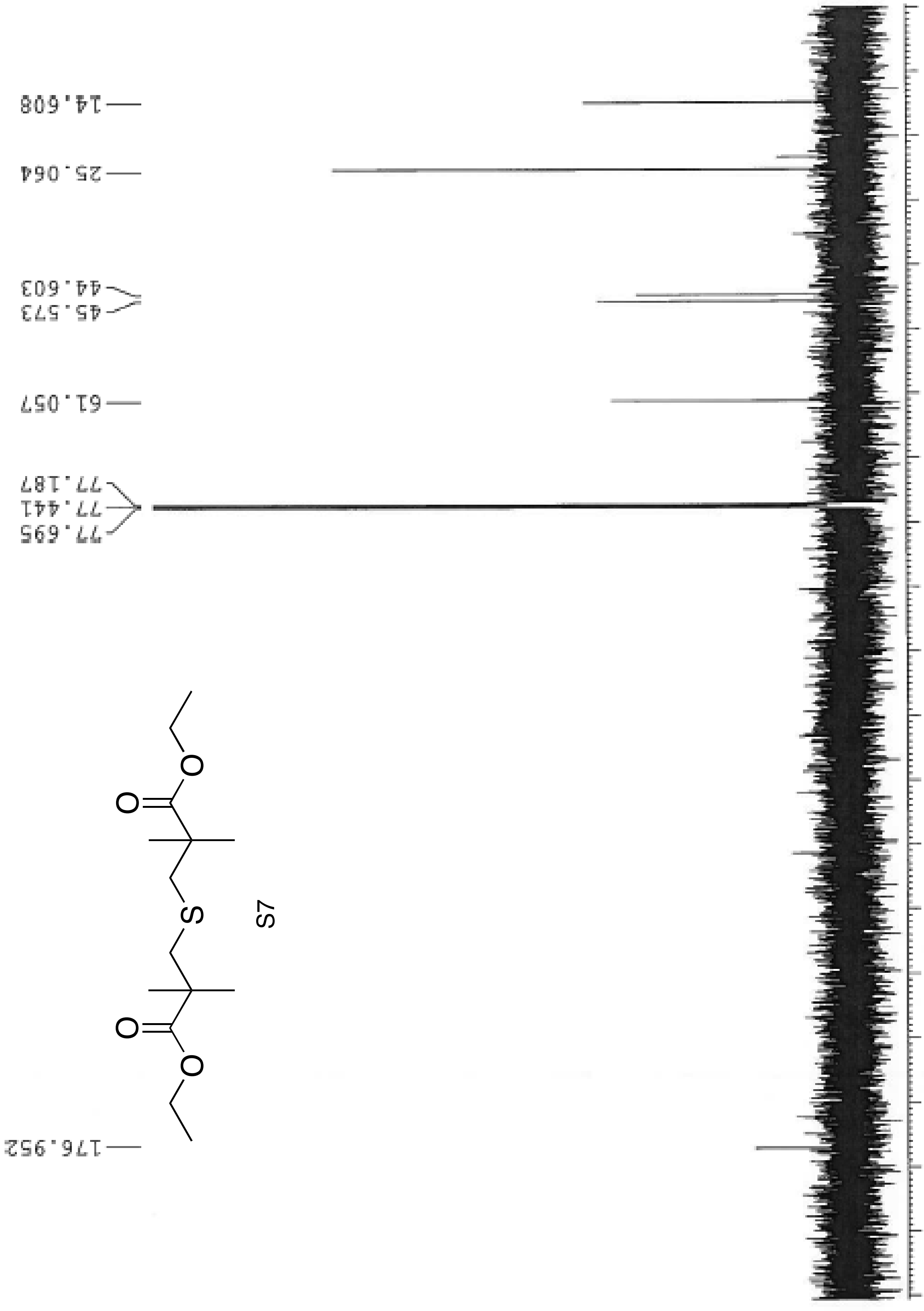
14.608
25.064
44.603
45.573
61.057
77.187
77.441
77.695
176.952
ppm
O
O
S
O
O

S7

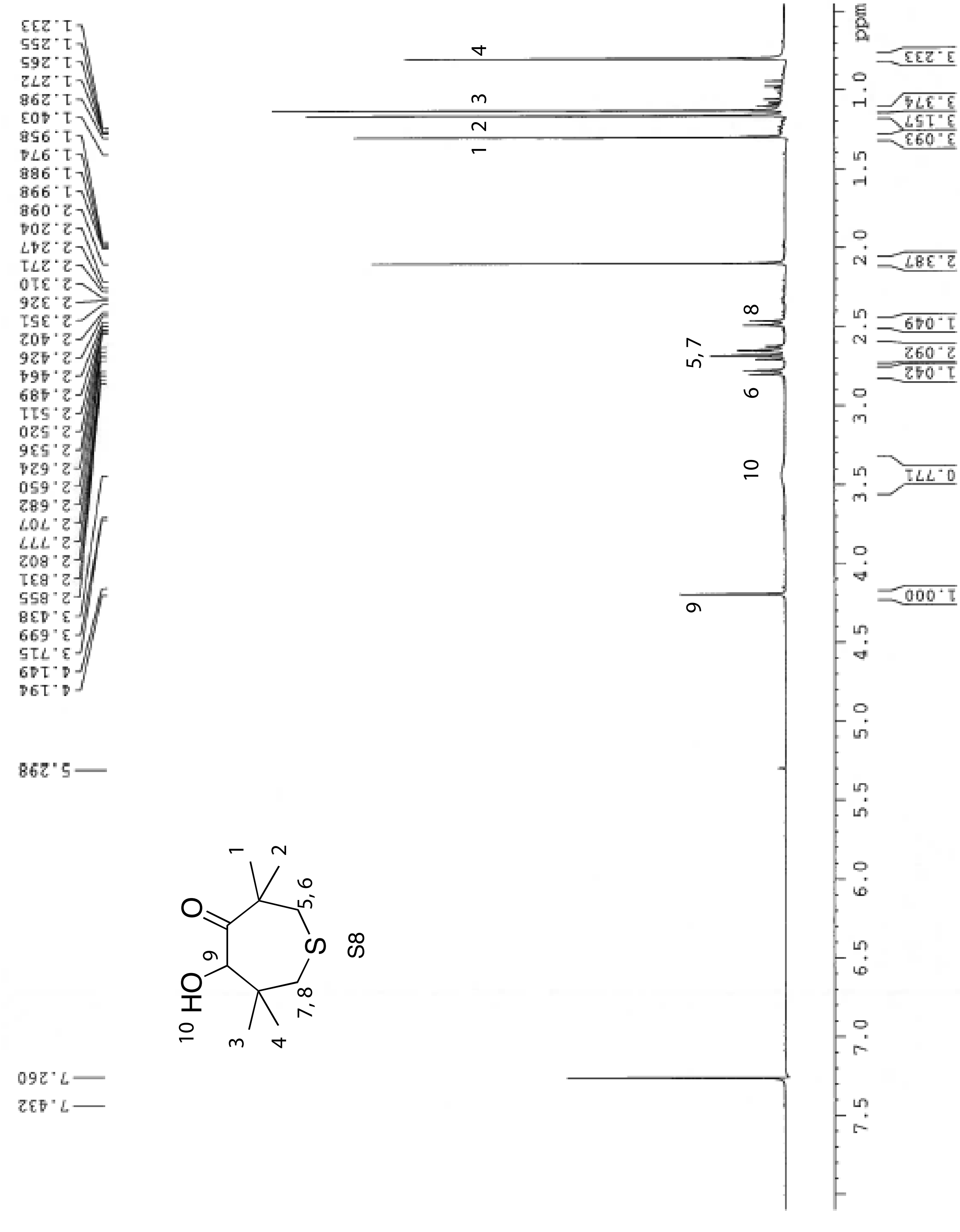

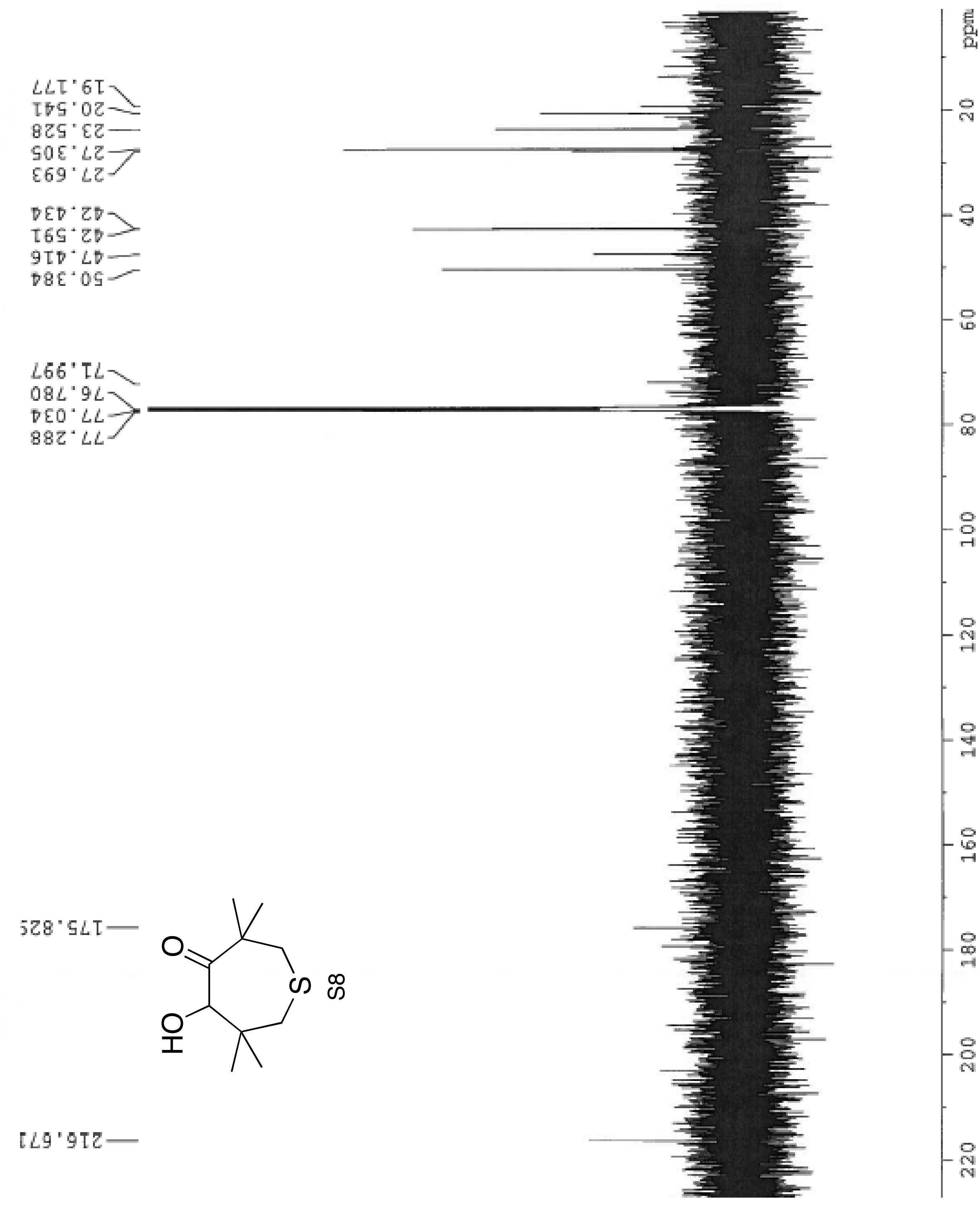
19.177
20.541
23.528
27.305
27.693
42.434
42.591
47.416
50.384
71.997
76.780
77.034
77.288
175.825
216.671
HO
O
S
S8
ppm
20
40
60
80
100
120
140
160
180
200
220

GdA4-159A
AVB-400 ZBO Proton starting parameters. 6/11/03 RN

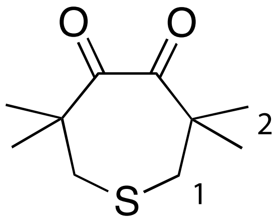

S9

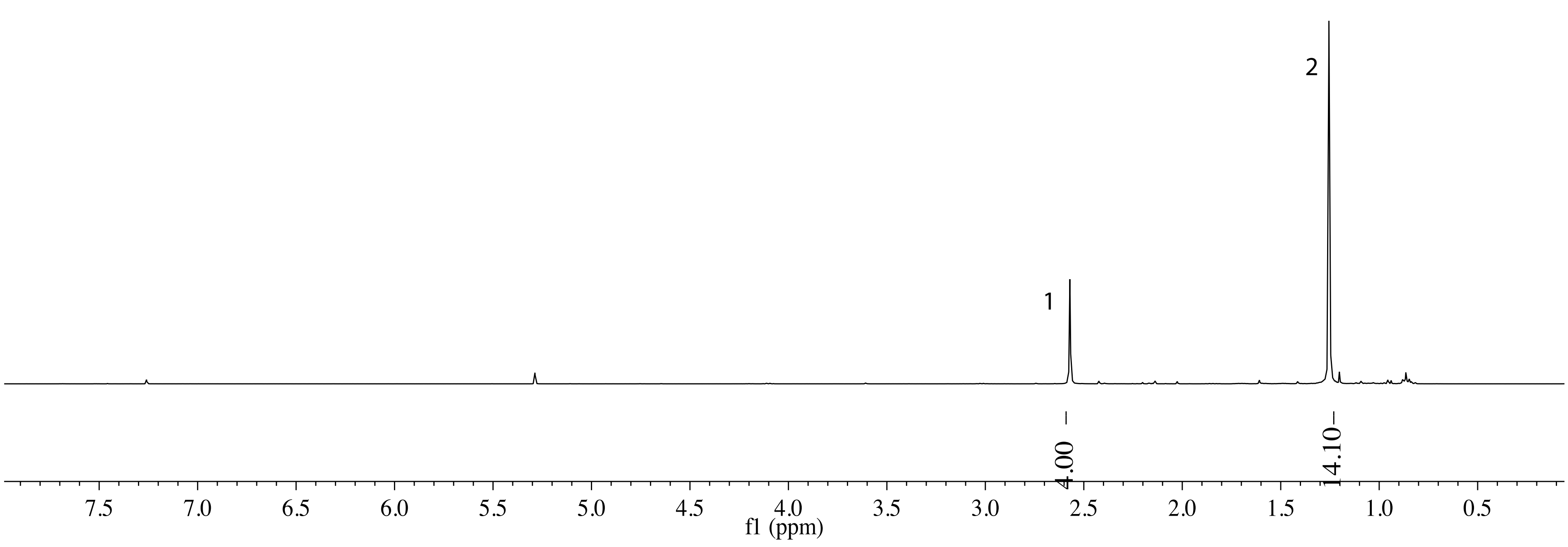

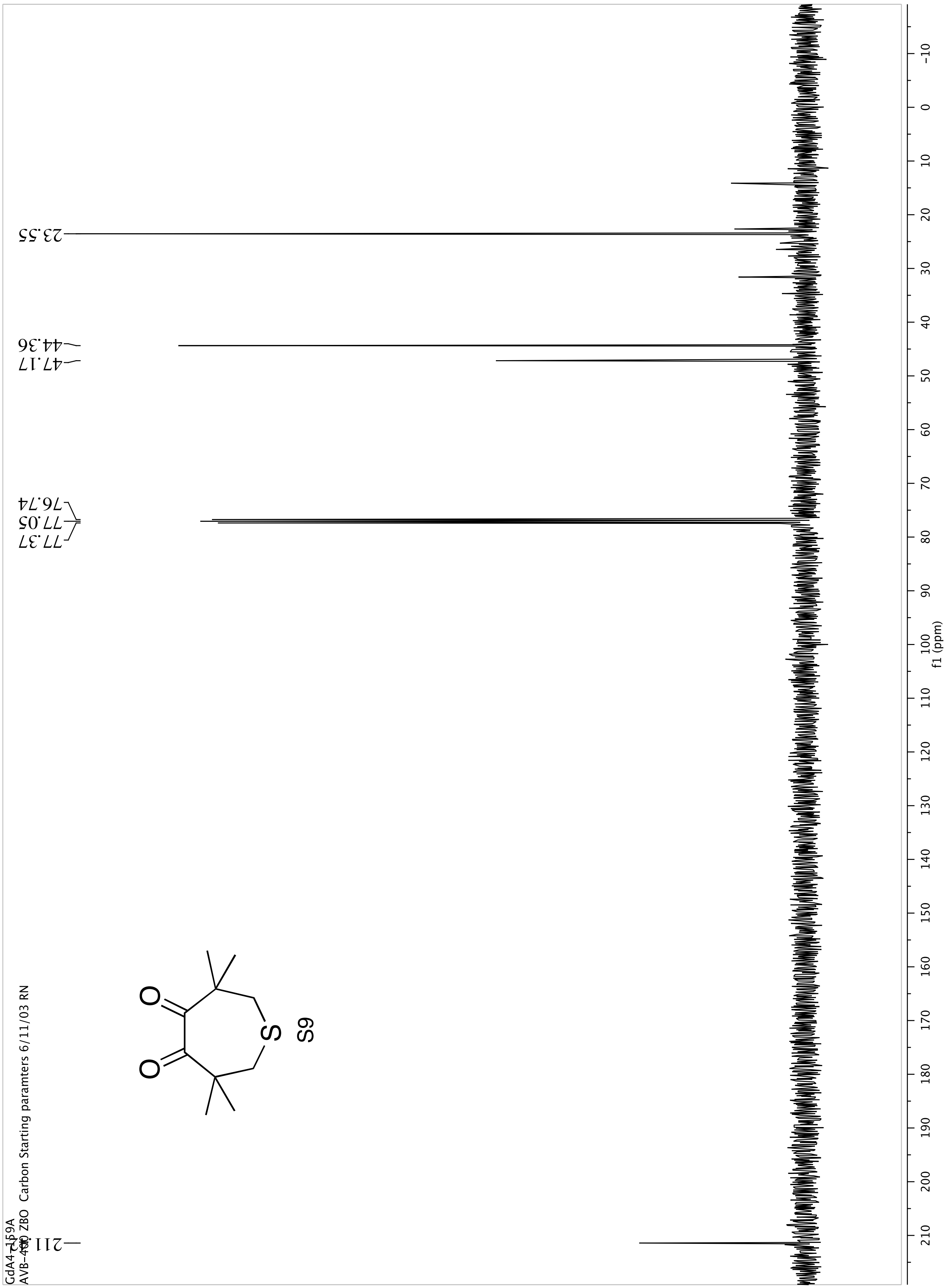
GdA4-159A
AVB-400 ZBO Carbon Starting paramters 6/11/03 RN
211.
77.37
77.05
76.74
47.17
44.36
23.55
S9
f1 (ppm)

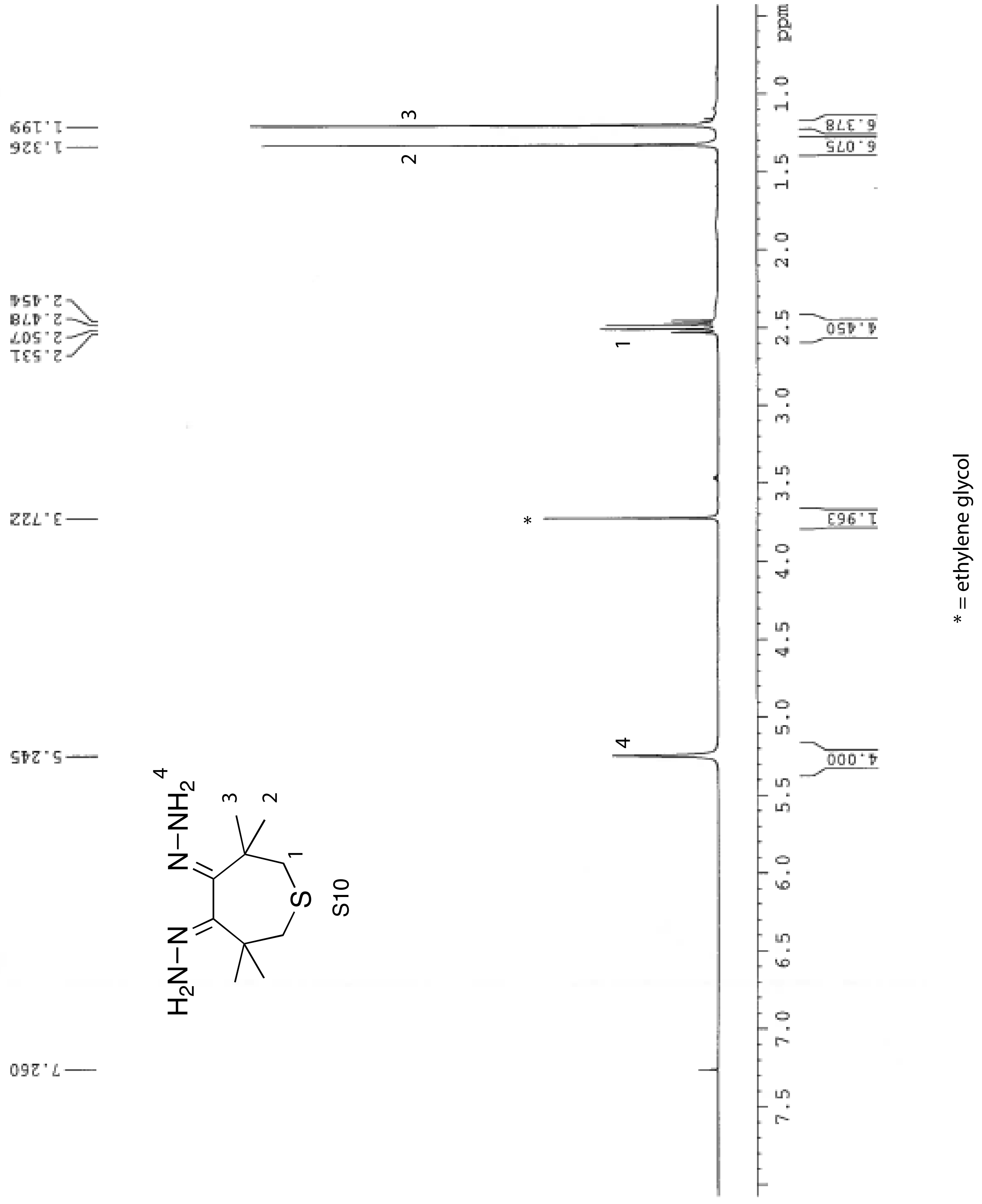
* = ethylene glycol
S10
ppm

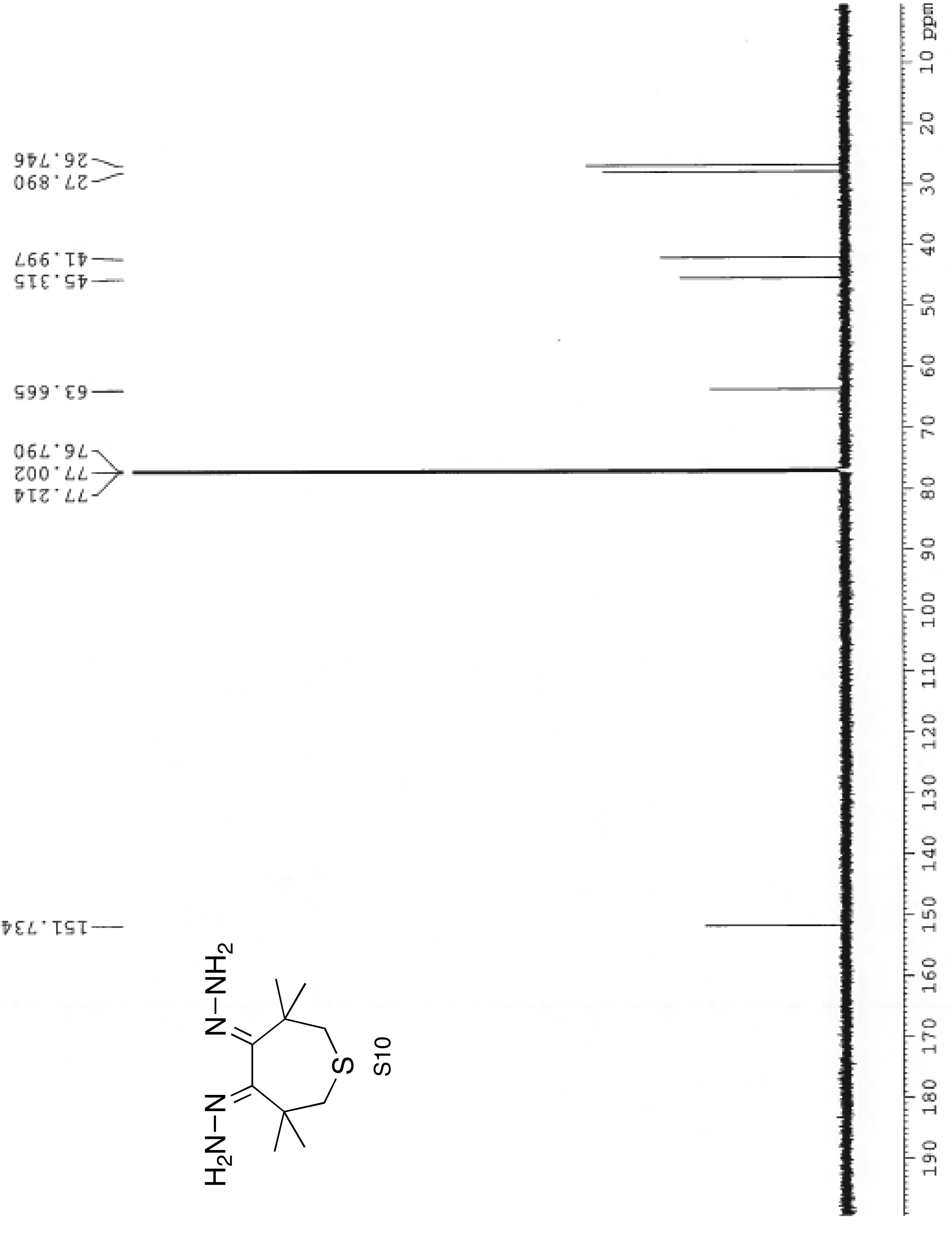
151.734
77.214
77.002
76.790
63.665
45.315
41.997
27.890
26.746
ppm
H2N–N
N–NH2
S
S10

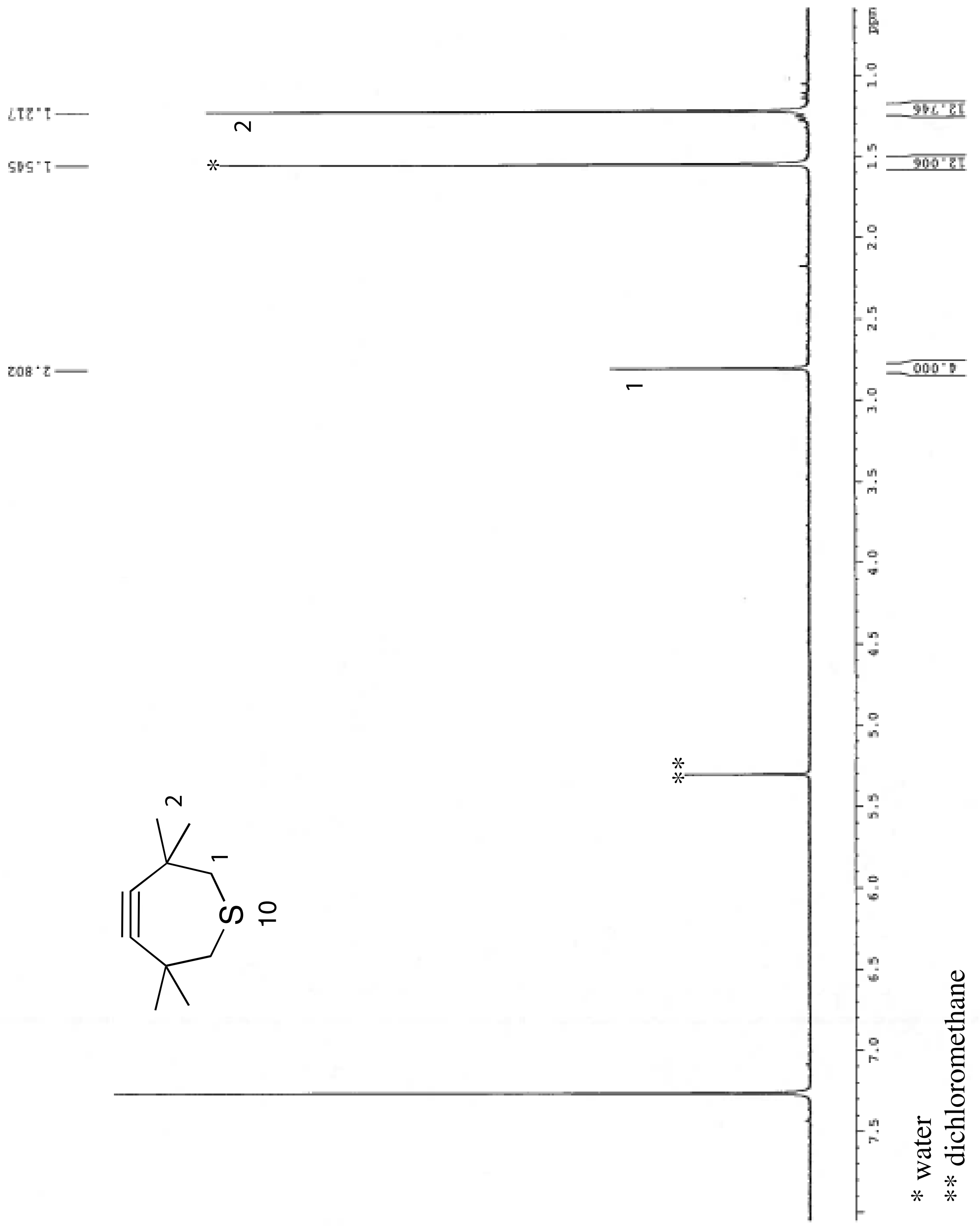
1.217
1.585
2.802
12.746
12.006
4.000
ppm
2
1
S
10
*
**
* water
** dichloromethane

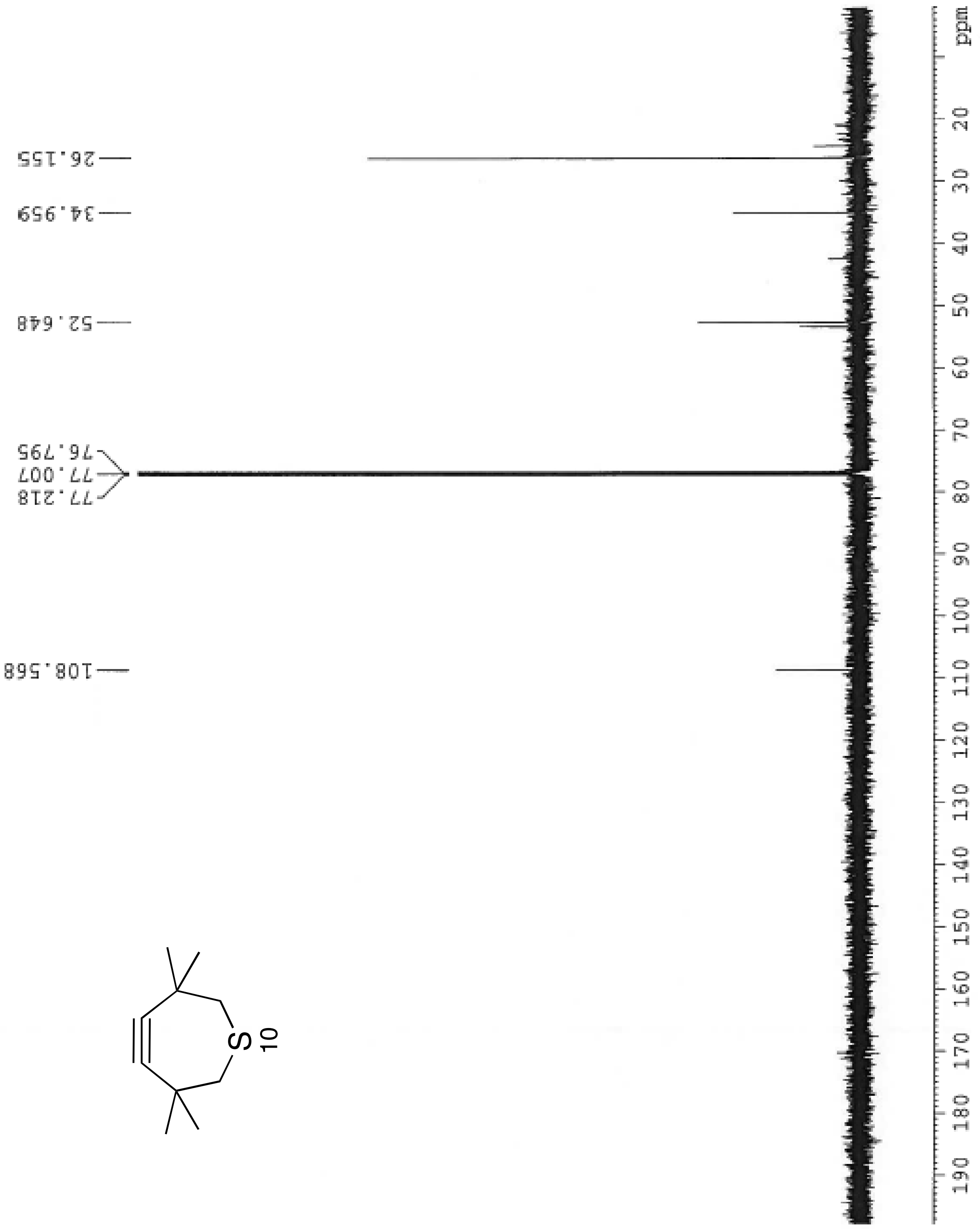
26.155
34.959
52.648
76.795
77.007
77.218
108.568
ppm
20 30 40 50 60 70 80 90 100 110 120 130 140 150 160 170 180 190
S
10

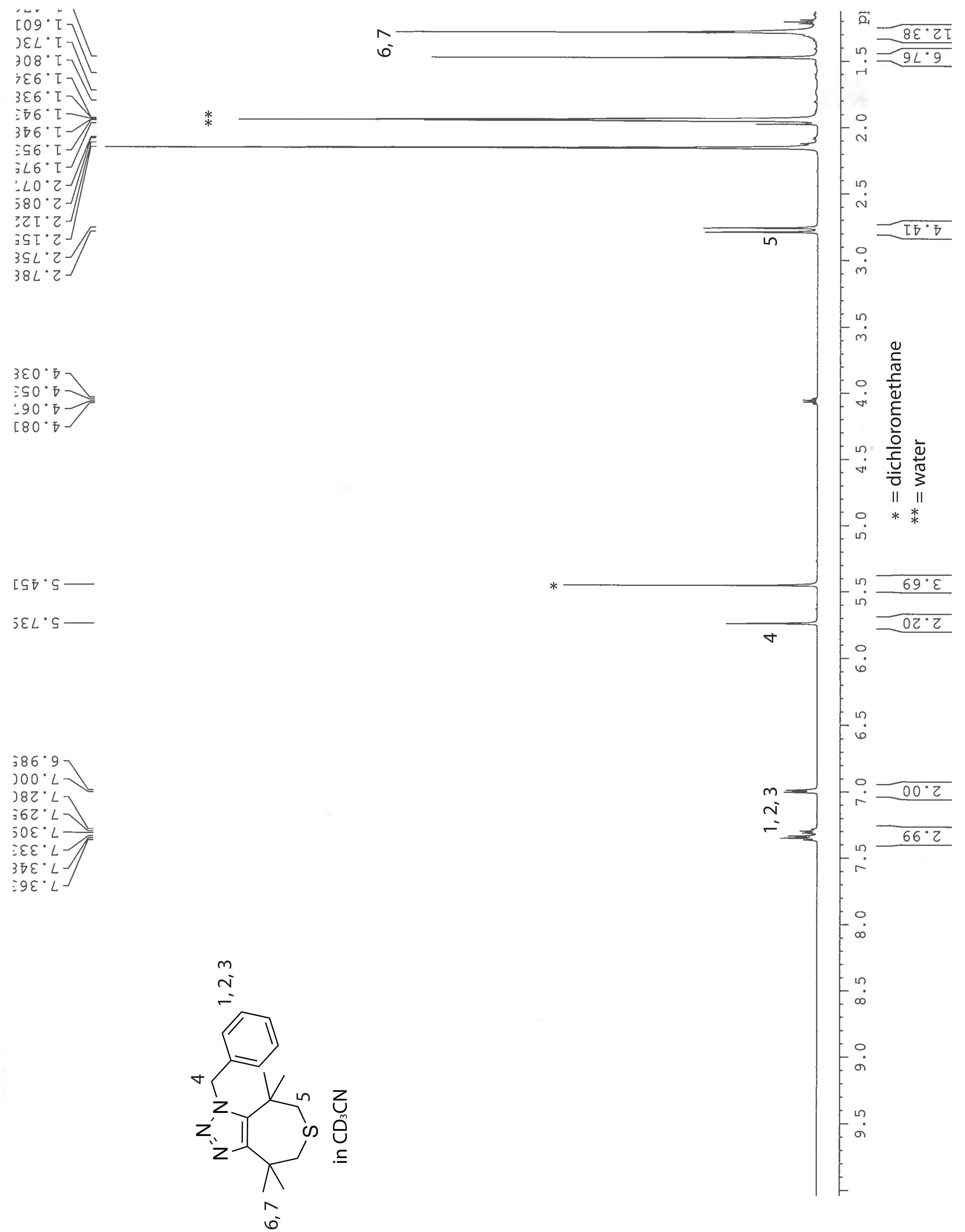
6, 7
**
5
*
4
1, 2, 3
* = dichloromethane
** = water
in CD3CN
12.38
6.76
4.41
3.69
2.20
2.00
2.99

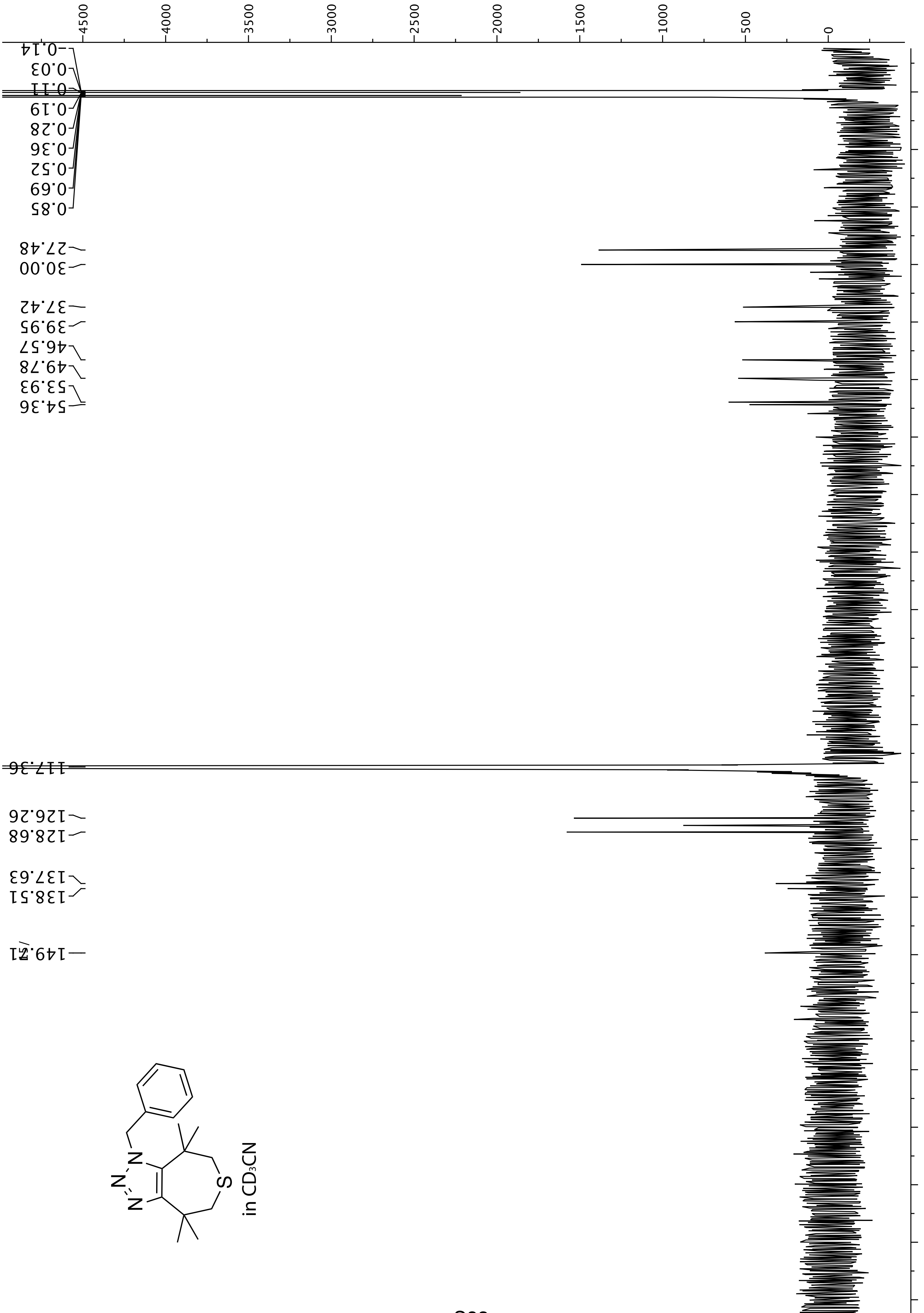
-0.14
0.03
0.11
0.19
0.28
0.36
0.52
0.69
0.85
27.48
30.00
37.42
39.95
46.57
49.78
53.93
54.36
117.36
126.26
128.68
137.63
138.51
f1 (ppm)
in CD3CN
N
S

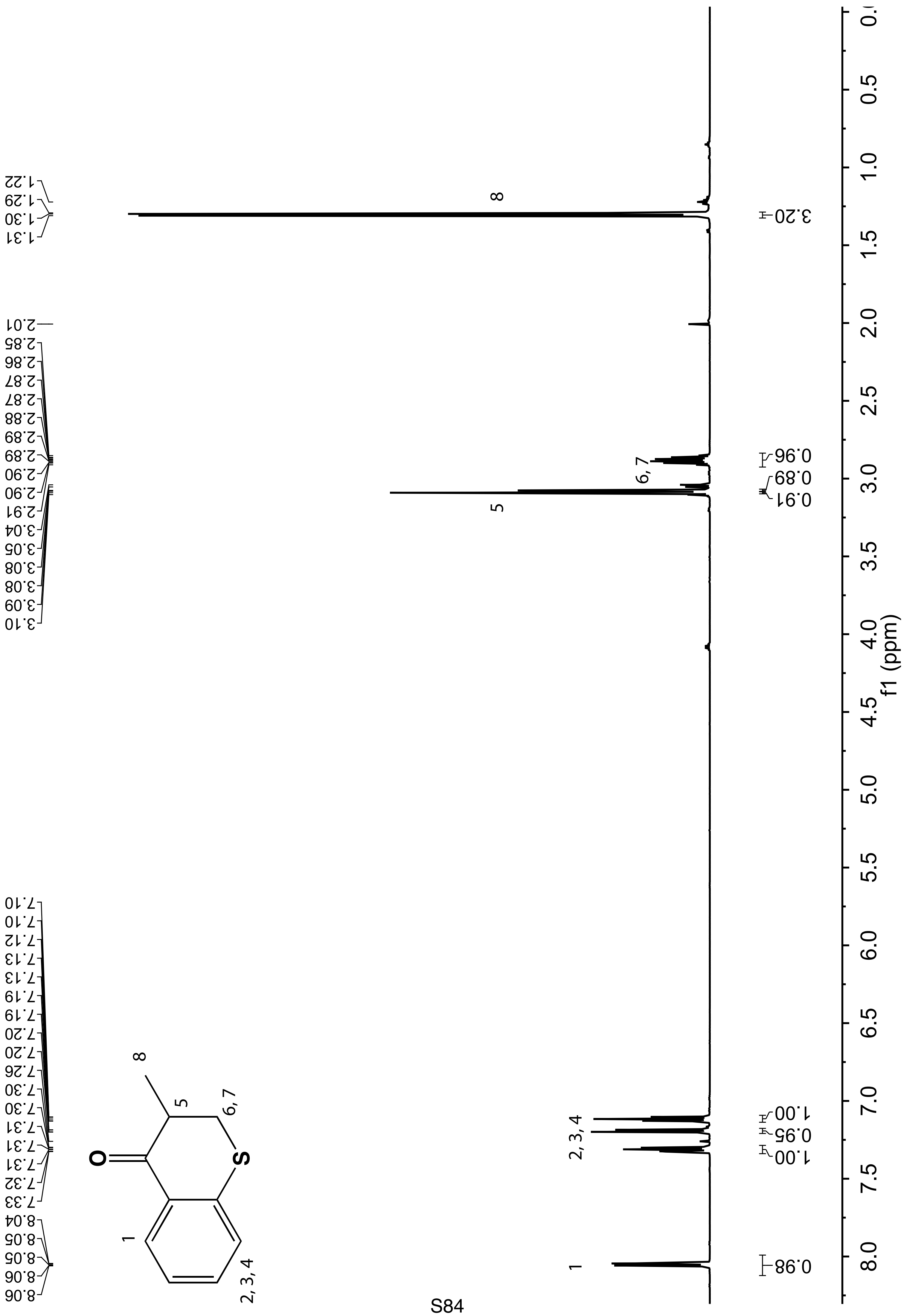

8.06
8.06
8.05
8.05
8.04
7.33
7.32
7.31
7.31
7.31
7.30
7.30
7.26
7.20
7.20
7.19
7.19
7.13
7.13
7.12
7.10
7.10
3.10
3.09
3.08
3.08
3.05
3.04
2.91
2.90
2.90
2.89
2.89
2.88
2.87
2.87
2.86
2.85
2.01
1.31
1.30
1.29
1.22
O
S
1
2, 3, 4
5
6, 7
8
0.98
1.00
0.95
1.00
0.91
0.89
0.96
3.20
f1 (ppm)

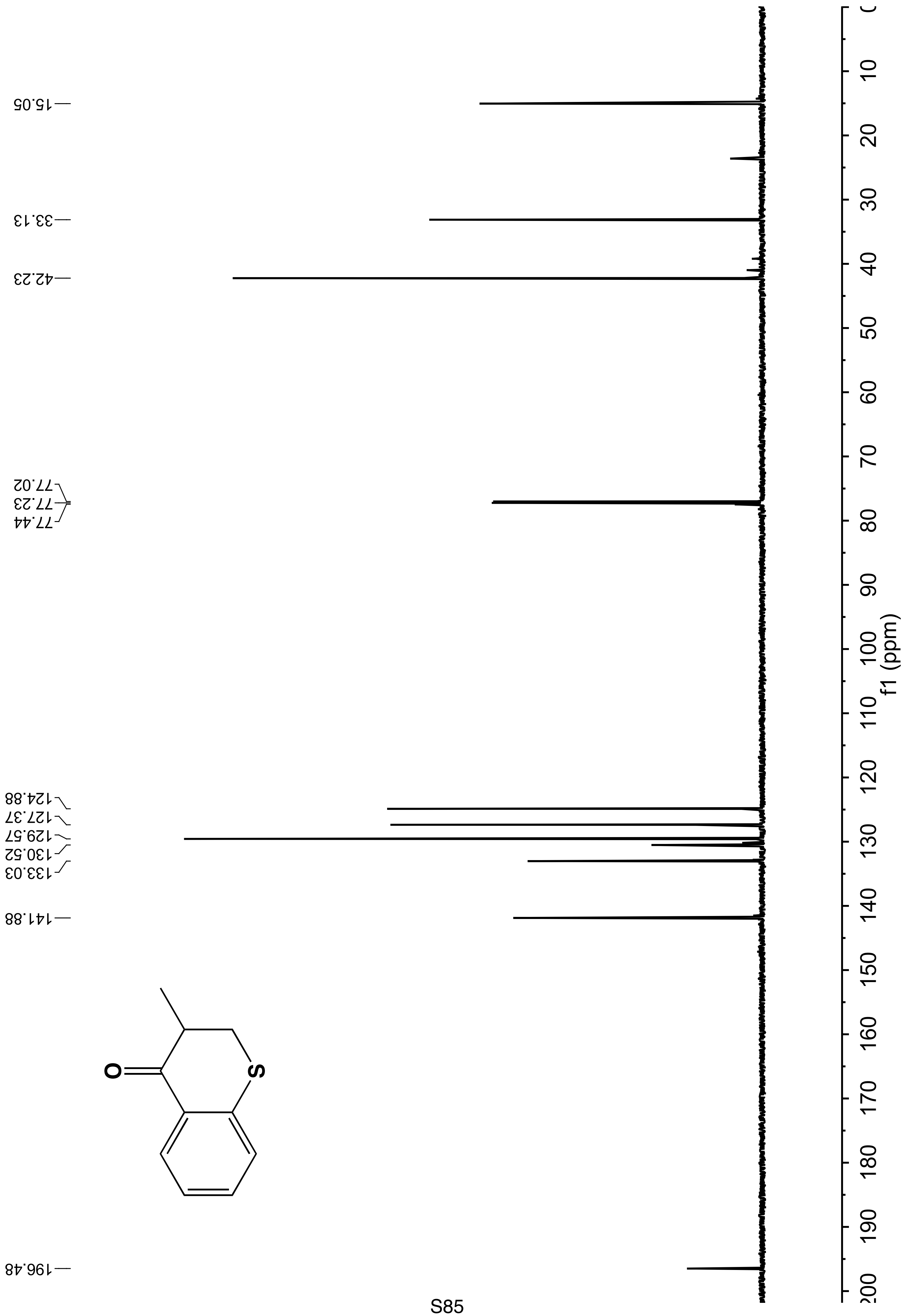
15.05
33.13
42.23
77.02
77.23
77.44
124.88
127.37
129.57
130.52
133.03
141.88
196.48
f1 (ppm)
S
O

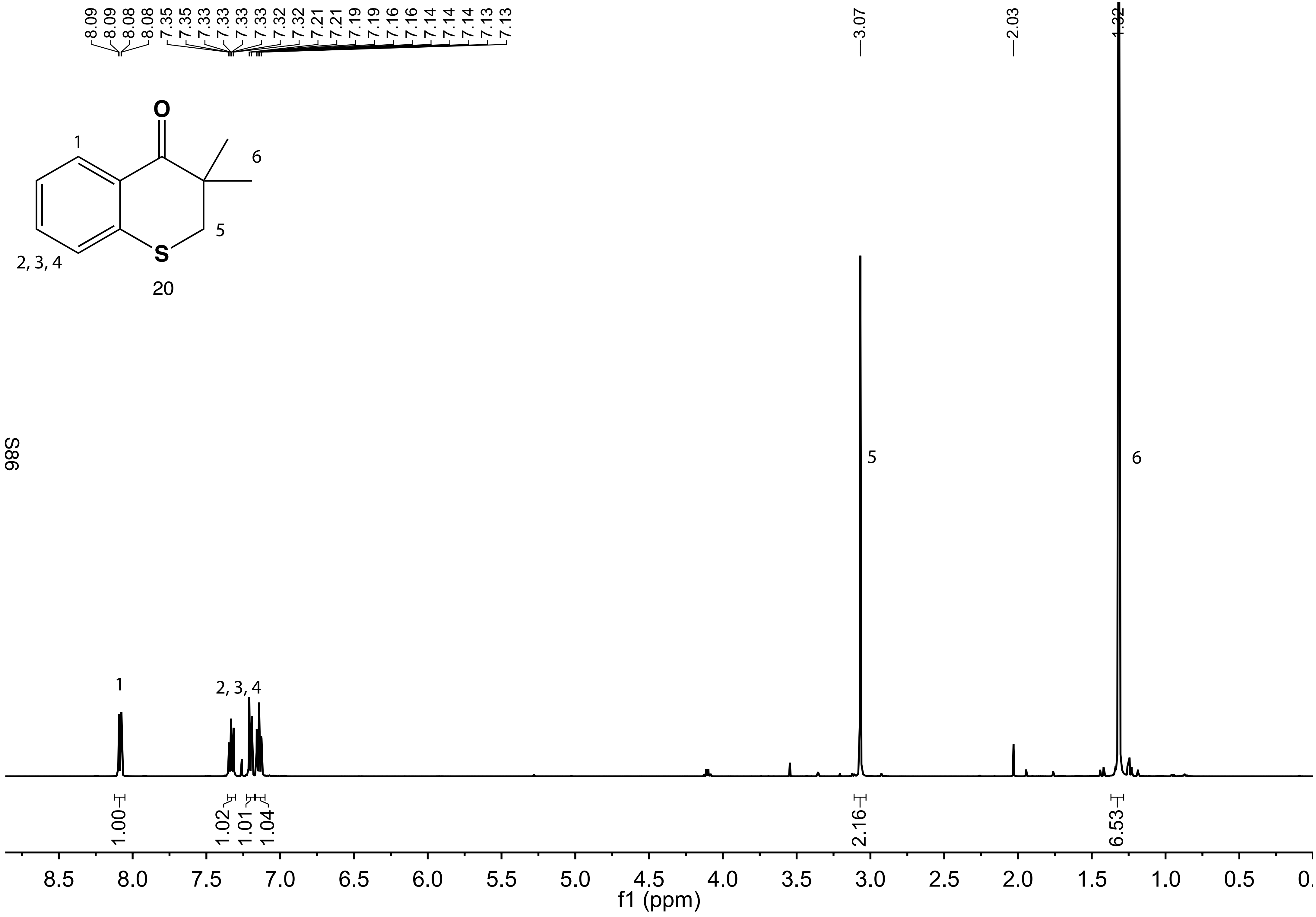
8.09 8.09 8.08 8.08 7.35 7.35 7.33 7.33 7.33 7.33 7.32 7.32 7.21 7.21 7.19 7.19 7.16 7.16 7.14 7.14 7.14 7.13 7.13
3.07
2.03
1.32
O
S
1
2, 3, 4
5
6
20
1.00
1.02
1.01
1.04
2.16
6.53
8.5 8.0 7.5 7.0 6.5 6.0 5.5 5.0 4.5 4.0 3.5 3.0 2.5 2.0 1.5 1.0 0.5
f1 (ppm)

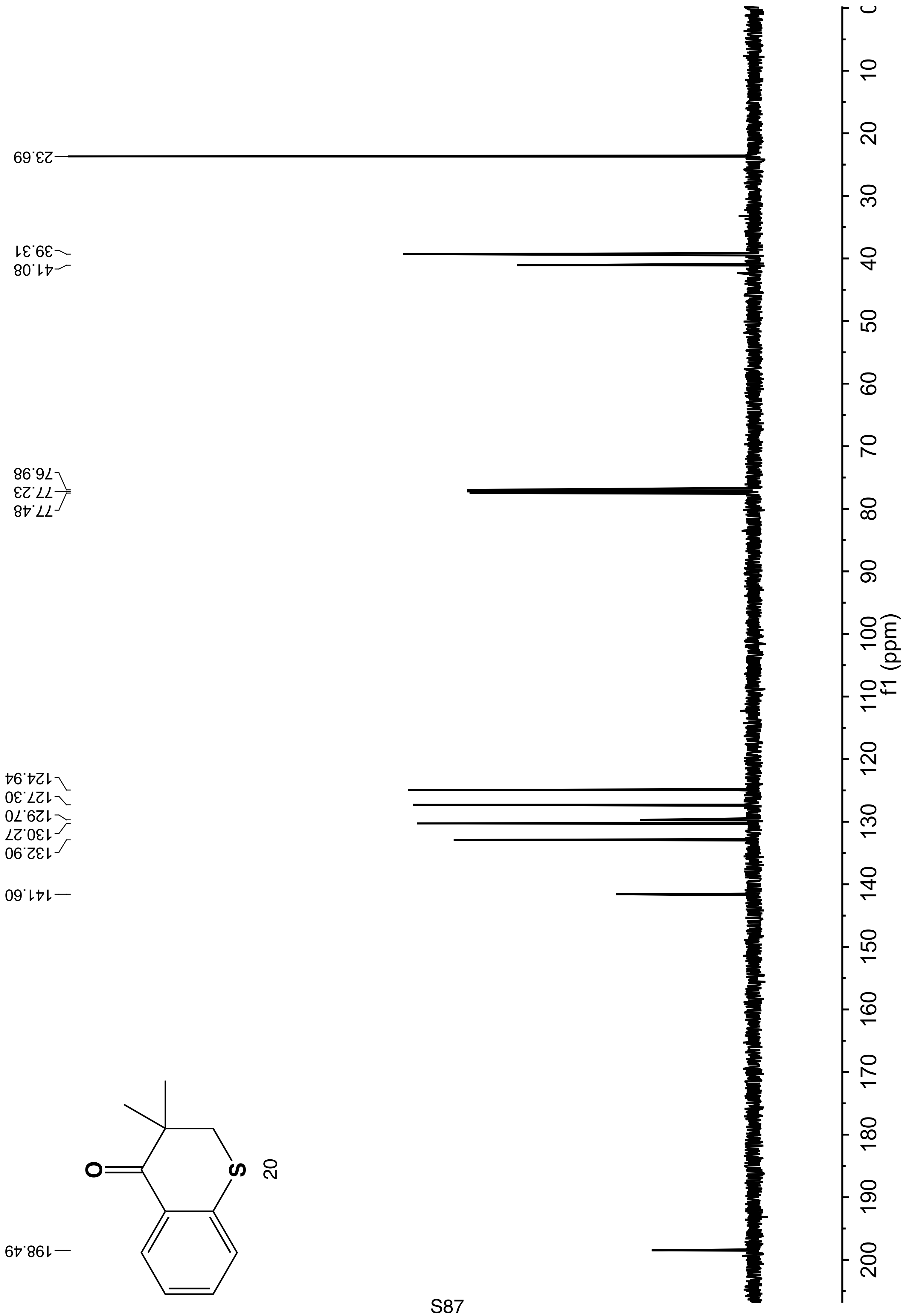
23.69
39.31
41.08
76.98
77.23
77.48
124.94
127.30
129.70
130.27
132.90
141.60
198.49
O
S
20
f1 (ppm)

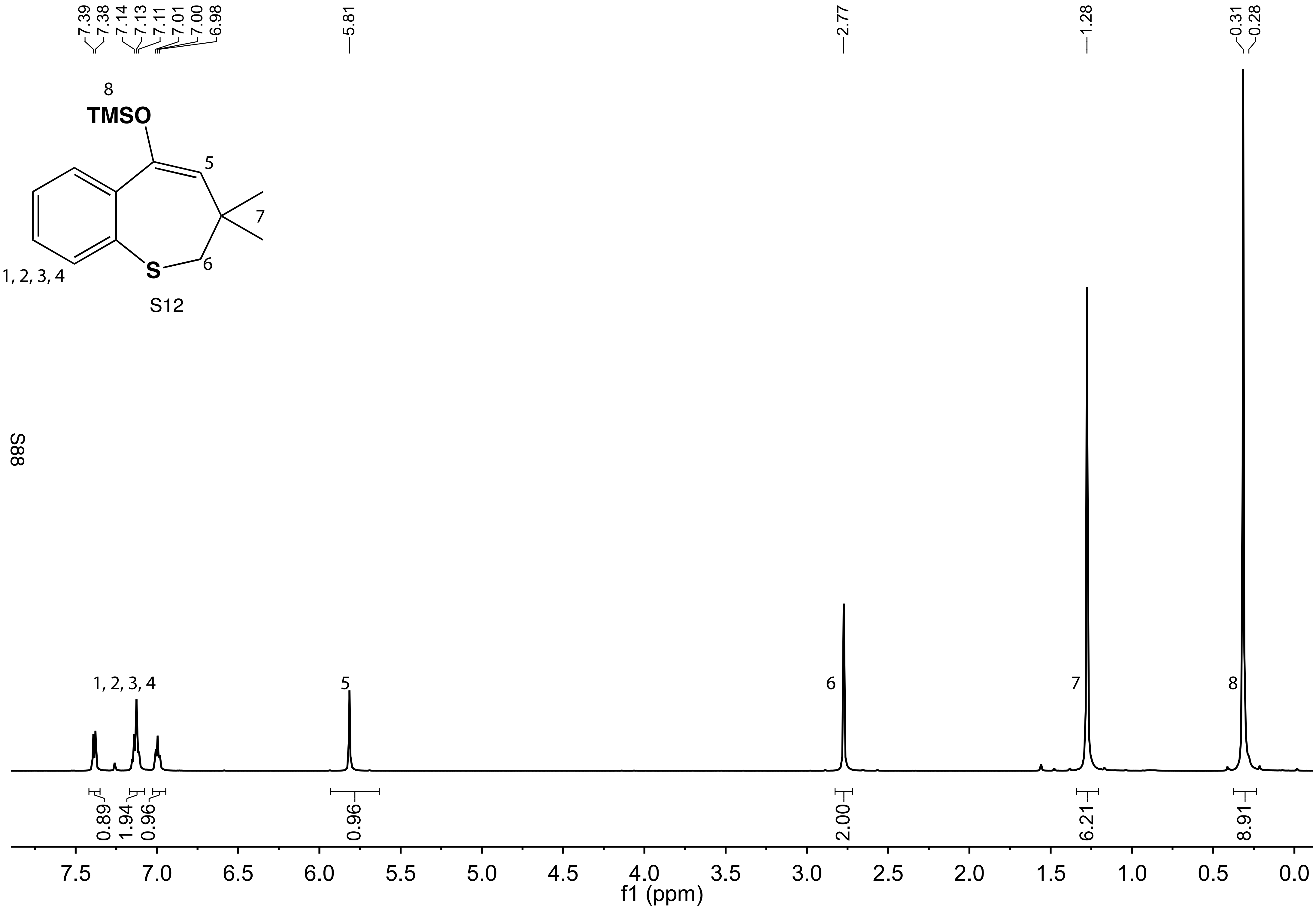
7.39
7.38
7.14
7.13
7.11
7.01
7.00
6.98
5.81
2.77
1.28
0.31
0.28
8
TMSO
5
7
6
S
1, 2, 3, 4
S12
1, 2, 3, 4
5
6
7
8
0.89
1.94
0.96
0.96
2.00
6.21
8.91
7.5
7.0
6.5
6.0
5.5
5.0
4.5
4.0
3.5
3.0
2.5
2.0
1.5
1.0
0.5
0.0
f1 (ppm)

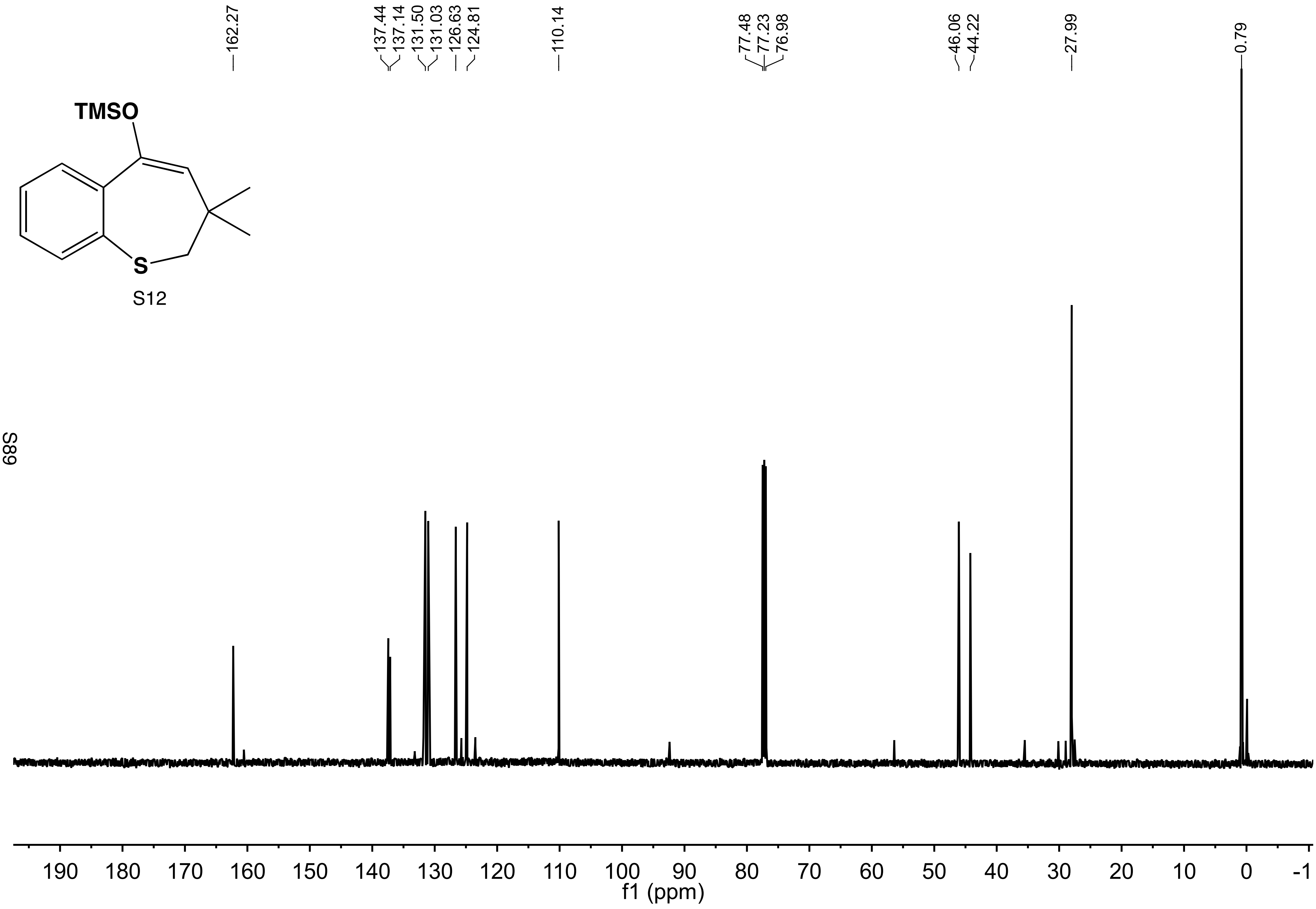
162.27
137.44
137.14
131.50
131.03
126.63
124.81
110.14
77.48
77.23
76.98
46.06
44.22
27.99
0.79
TMSO
S
S12
190 180 170 160 150 140 130 120 110 100 90 80 70 60 50 40 30 20 10 0 -1
f1 (ppm)

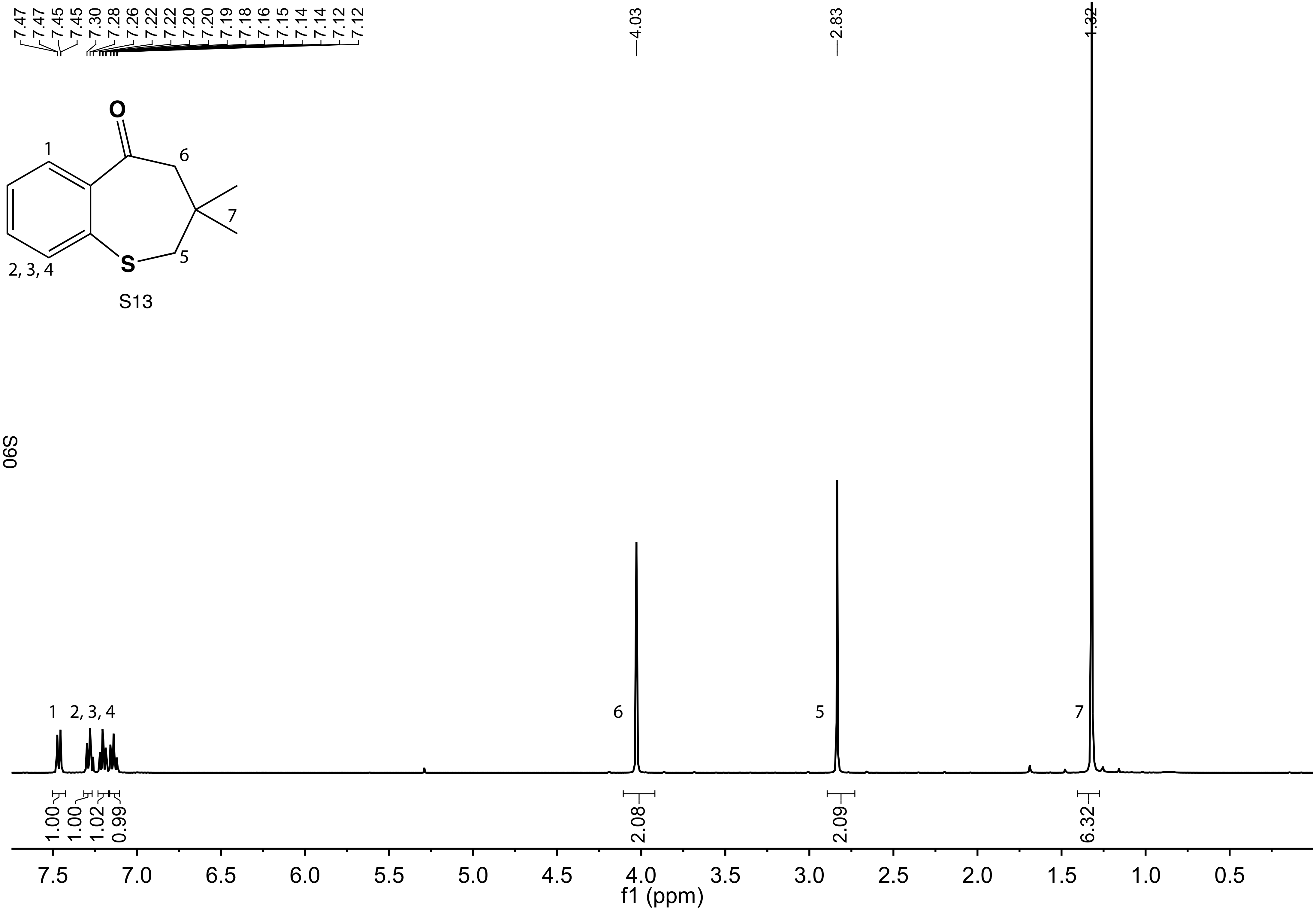
7.47
7.47
7.45
7.45
7.30
7.28
7.26
7.22
7.22
7.20
7.20
7.19
7.18
7.16
7.15
7.14
7.14
7.12
7.12
4.03
2.83
1.32
O
S
1
2, 3, 4
5
6
7
S13
1 2, 3, 4
6
5
7
1.00
1.00
1.02
0.99
2.08
2.09
6.32
7.5
7.0
6.5
6.0
5.5
5.0
4.5
4.0
3.5
3.0
2.5
2.0
1.5
1.0
0.5
f1 (ppm)

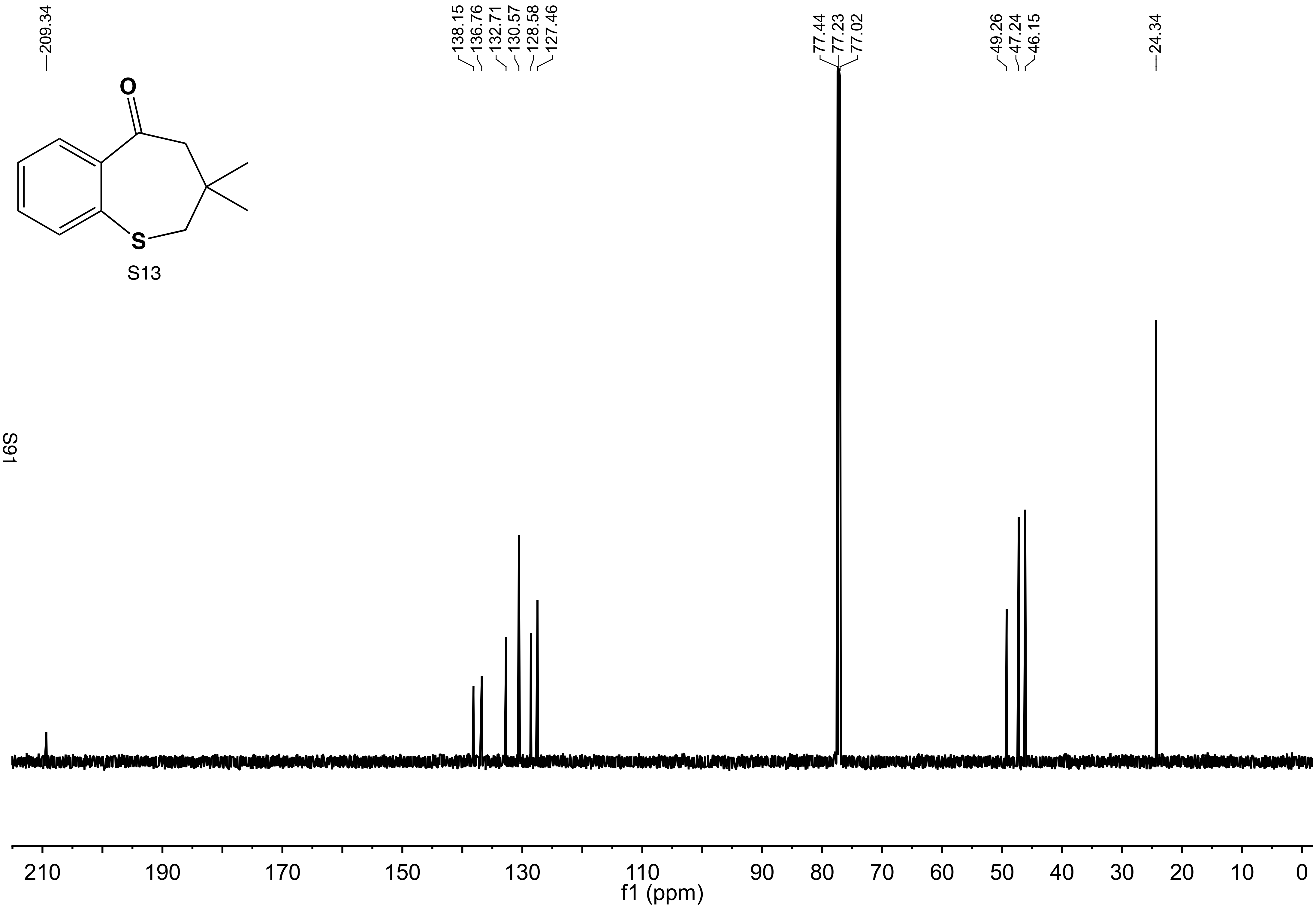
209.34
138.15
136.76
132.71
130.57
128.58
127.46
77.44
77.23
77.02
49.26
47.24
46.15
24.34
O
S
S13
210
190
170
150
130
110
90
80
70
60
50
40
30
20
10
0
f1 (ppm)

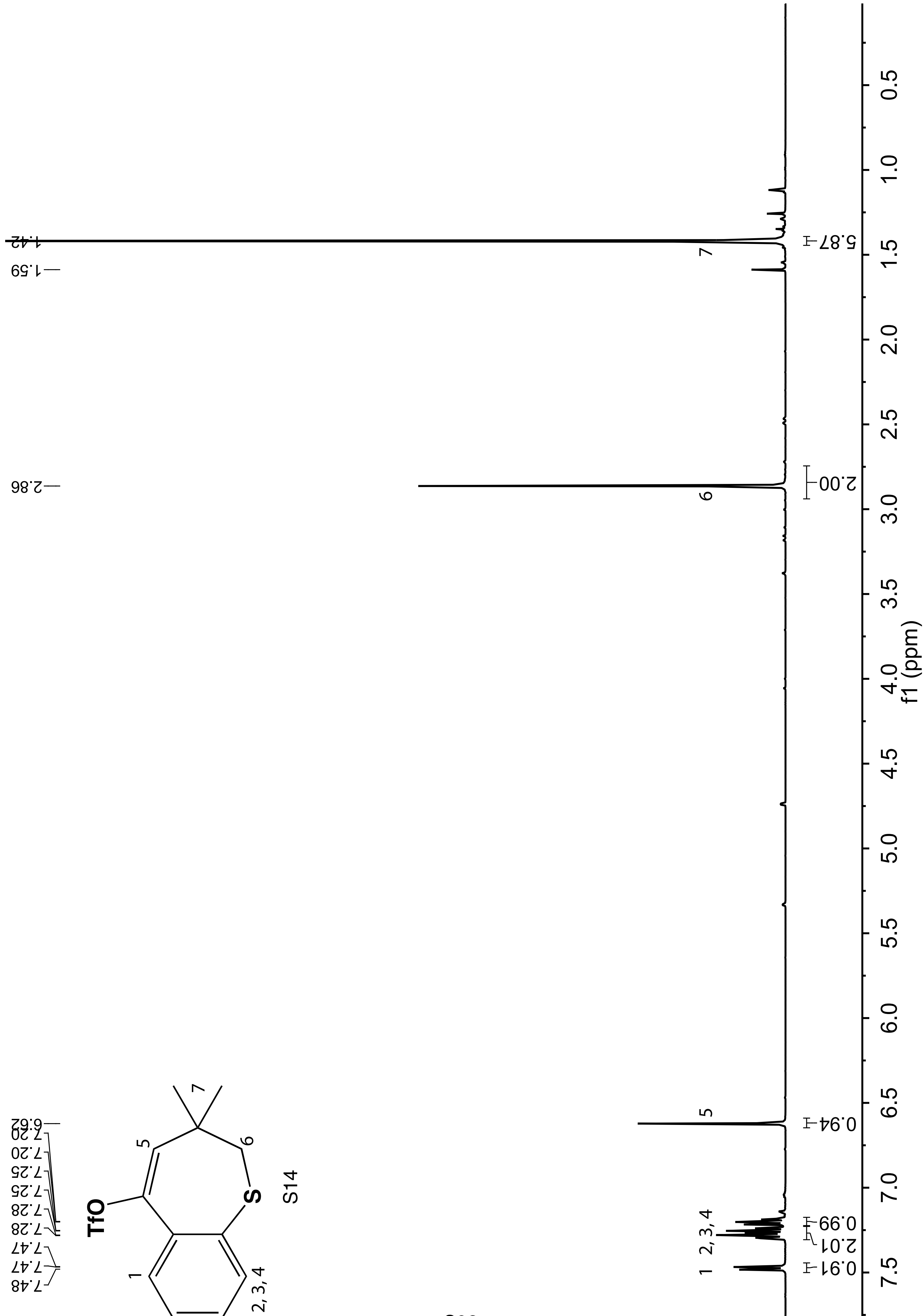
7.48
7.47
7.47
7.28
7.28
7.25
7.25
7.20
7.20
6.62
2.86
1.59
1.42
TfO
S
S14
1
2, 3, 4
5
6
7
0.91
2.01
0.99
0.94
2.00
5.87
f1 (ppm)

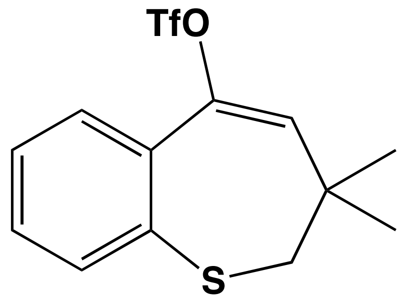

S14

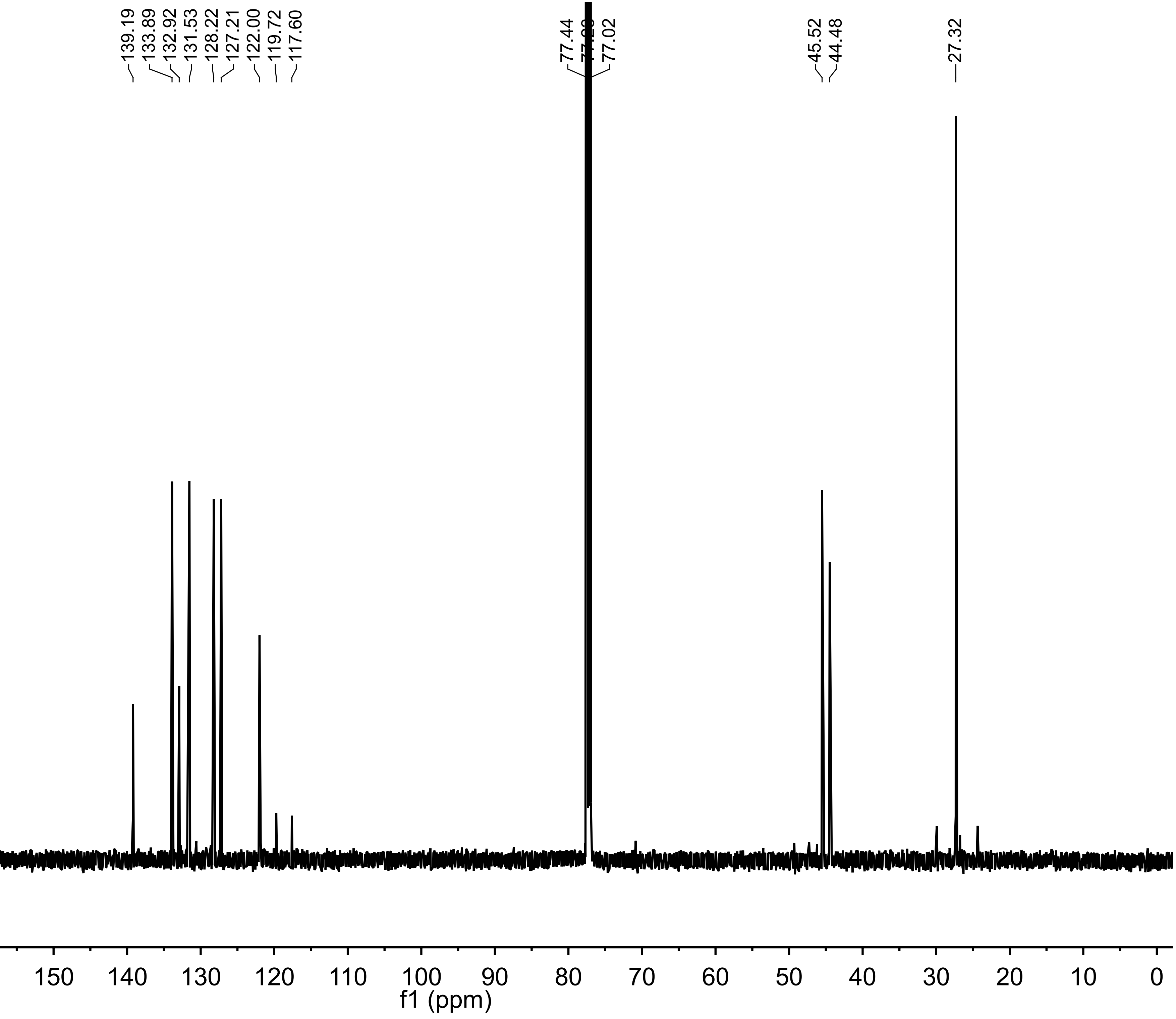
158.26
139.19
133.89
132.92
131.53
128.22
127.21
122.00
119.72
117.60
77.44
77.02
45.52
44.48
27.32
190
180
170
160
150
140
130
120
110
100
90
80
70
60
50
40
30
20
10
0
f1 (ppm)

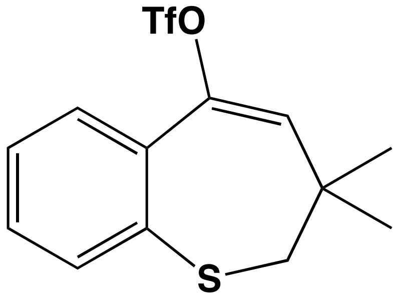

S14

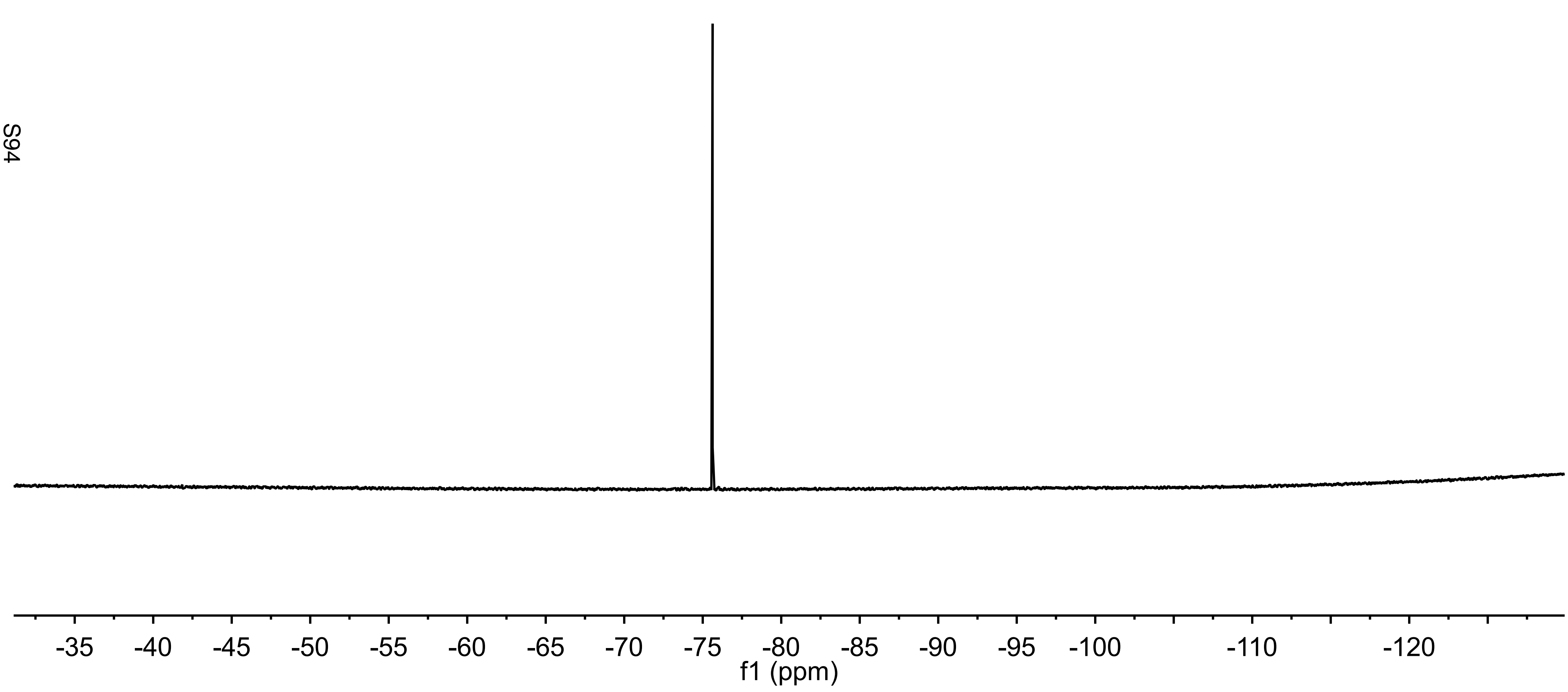

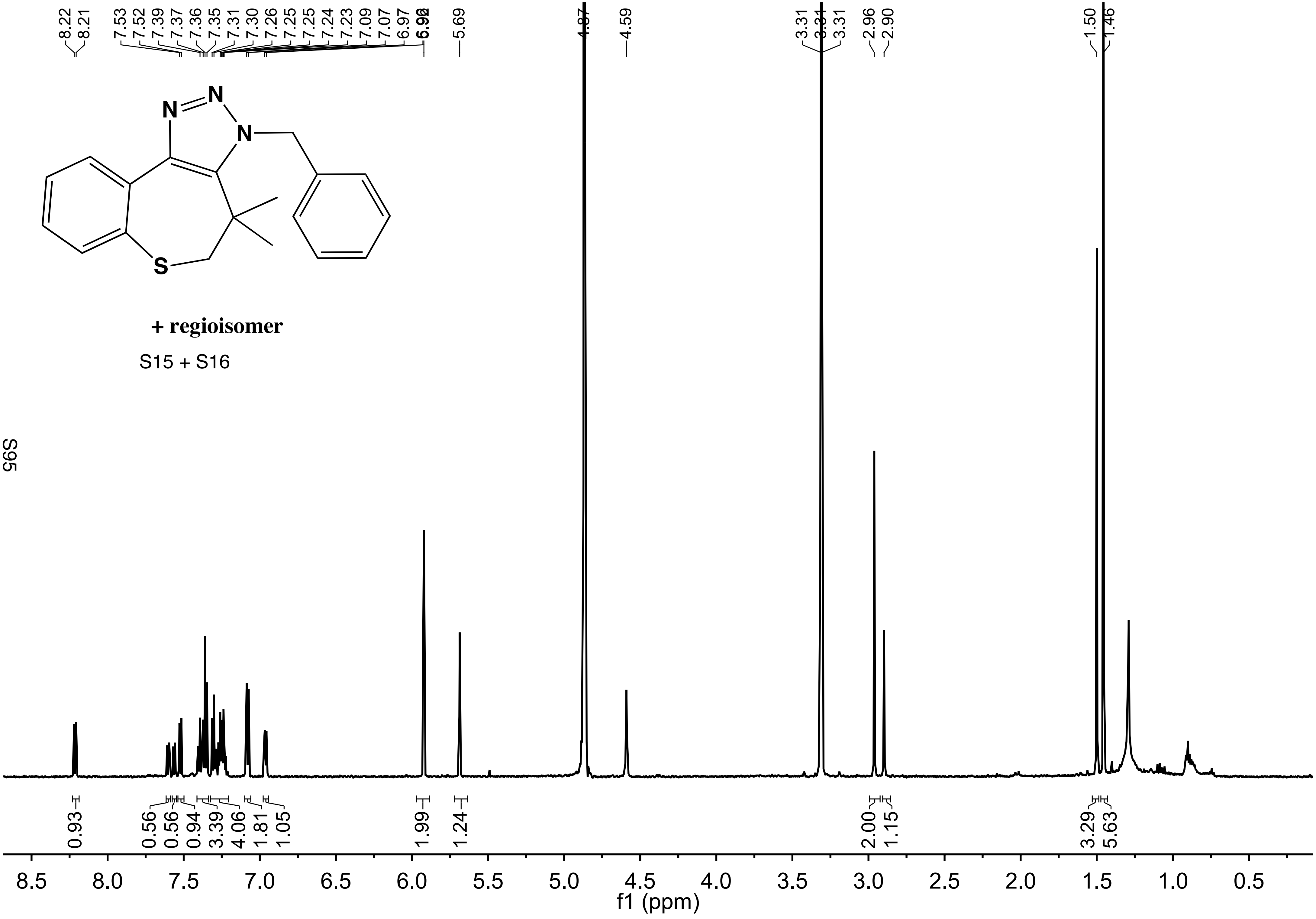
8.22
8.21
7.53
7.52
7.39
7.37
7.36
7.35
7.31
7.30
7.26
7.25
7.25
7.24
7.23
7.09
7.07
6.97
6.96
5.69
4.87
4.59
3.31
3.31
3.31
2.96
2.90
1.50
1.46
N
N
N
S
+ regioisomer
S15 + S16
0.93
0.56
0.56
0.94
3.39
4.06
1.81
1.05
1.99
1.24
2.00
1.15
3.29
5.63
8.5
8.0
7.5
7.0
6.5
6.0
5.5
5.0
4.5
4.0
3.5
3.0
2.5
2.0
1.5
1.0
0.5
f1 (ppm)

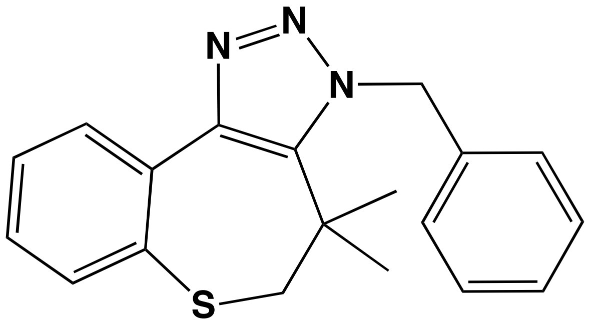

+ regioisomer

S15 + S16

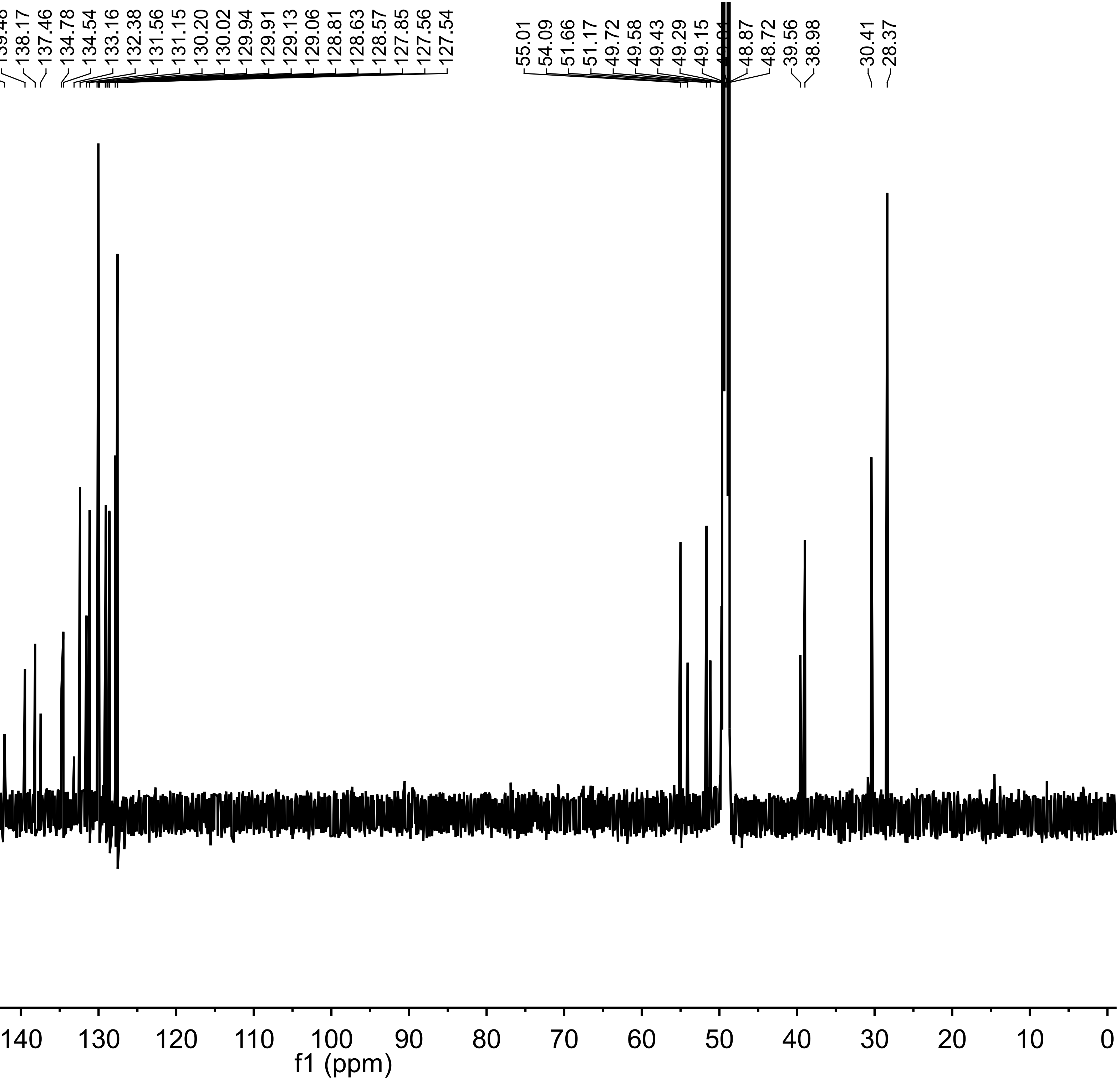

Supplement: Supplementary file 1 [file anie0051-2443-SD1.pdf]
